# *Asxl1* deficiency in embryonic fibroblasts leads to cellular senescence via impairment of the AKT-E2F pathway and Ezh2 inactivation

**DOI:** 10.1038/s41598-017-05564-x

**Published:** 2017-07-12

**Authors:** Hye Sook Youn, Tae-Yoon Kim, Ui-Hyun Park, Seung-Tae Moon, So-Jung An, Yong-Kyu Lee, Jin-Taek Hwang, Eun-Joo Kim, Soo-Jong Um

**Affiliations:** 1 Department of Bioscience and Biotechnology, BK21 Graduate Program, Sejong University, 209 Neungdong-ro, Gwangjin-gu, Seoul 143-747 Korea; 2 Korea Food Research Institute, Bundang-gu, Seongnam-si 463-746 Korea; 3 Department of Molecular Biology, Dankook University, Gyeonggi-do, 448-701 Korea

## Abstract

Although *ASXL1* mutations are frequently found in human diseases, including myeloid leukemia, the cell proliferation–associated function of ASXL1 is largely unknown. Here, we explored the molecular mechanism underlying the growth defect found in *Asxl1*-deficient mouse embryonic fibroblasts (MEFs). We found that Asxl1, through amino acids 371 to 655, interacts with the kinase domain of AKT1. In *Asxl1*-null MEFs, IGF-1 was unable to induce AKT1 phosphorylation and activation; p27Kip1, which forms a ternary complex with ASXL1 and AKT1, therefore remained unphosphorylated. Hypophosphorylated p27Kip1 is able to enter the nucleus, where it prevents the phosphorylation of Rb; this ultimately leads to the down-regulation of E2F target genes as confirmed by microarray analysis. We also found that senescence-associated (SA) genes were upregulated and that SA β-gal staining was increased in *Asxl1* ^−/−^ MEFs. Further, the treatment of an AKT inhibitor not only stimulated nuclear accumulation of p27Kip1 leading to E2F inactivation, but also promoted senescence. Finally, *Asxl1* disruption augmented the expression of *p16Ink4a* as result of the defect in Asxl1*-*Ezh2 cooperation. Overall, our study provides the first evidence that Asxl1 both activates the AKT-E2F pathway and cooperates with Ezh2 through direct interactions at early embryonic stages, reflecting that *Asxl1* disruption causes cellular senescence.

## Introduction

The additional sex comb (*Asx*) gene was originally identified in *Drosophila* as an enhancer of trithorax group (TrxG) and Polycomb group (PcG) proteins^1^. Three paralog Asx-like (*Asxl*) genes have been found in mammals, encoding ASXL1, ASXL2, and ASXL3^2–4^. We recently reported that ASXL1 is mainly located in the nucleus and acts as a dual-function regulator of nuclear receptors, either cooperating with SRC1 for activation or with HP1 for repression in the presence of a ligand^5, 6^. ASXL1 also seems to function as a tumor suppressor. Mutations of *ASXL1* are often found in human diseases and are mostly linked to acute myeloid leukemia^7^, myelodysplastic syndromes and chronic myelomonocytic leukemia^8^, and Bohring-Opitz syndrome^9^. Despite the somatic *ASXL1* mutations frequently reported in leukemia patients, the mechanisms by which *ASXL1* mutations cause cancer are not fully understood. Recent studies using leukemia cells from human patients with *ASXL1* mutations showed that ASXL1 interacts with histone methyltransferase EZH2, one of PRC2 members, to increase histone H3K27 tri-methylation (me3)^10^. In addition, Asxl1 deletion in mice was accompanied with reduction of H3K27me3. In contrast, loss of Bap1, one of Asxl1 binding partners, resulted in enhanced H3K27me3 level and EZH2-dependent transformation^11^, suggesting distinct, independent roles of Asxl1 and Bap1 in myeloid leukemogenesis.

AKT, also called protein kinase B, was identified as the cellular counterpart of a viral oncogene. Amplified AKT isoforms has been found in several types of human cancers^12–14^. Not only is AKT a key regulator of cell proliferation and survival^15^, but it also plays a role in the deregulation of cell cycle control by phosphorylating various target proteins^16^. Specific control of the cell cycle is critical for cell proliferation and growth during normal development and cancer progression. Cell cycle progression depends on cyclin-dependent kinases (CDKs), which are positively regulated by cyclins and negatively regulated by CDK inhibitors (CDKIs). The G1/S transition is checked by retinoblastoma protein (Rb). Hypophosphorylated Rb readily forms a complex with E2F1, a key transcription factor, which promotes the G1/S transition. When Rb is sequentially phosphorylated by CDK4/CDK6 and CDK2 in response to growth stimuli, E2F1 is released from the Rb/E2F complex and binds to the promoters of E2F target genes to induce their expression^17^. Inversely, CDKIs, such as p27Kip1, block Rb phosphorylation by binding to and inactivating the CDK4/6-cyclin D or CDK2-cyclin E complex, leading to E2F inactivation in the nucleus^18, 19^. Upon activation with various stimuli, AKT phosphorylates the nuclear localization signal of p27Kip1 and impairs its nuclear import. The consequent cytoplasmic accumulation of p27Kip1 results in Rb phosphorylation and thus E2F activation^20, 21^. Growth arrest is often associated with senescence, which has been proposed to be controlled by CDKIs including p16Ink4, p21Waf1, or p27Kip1^22, 23^. Inhibition of PI3K or AKT was recently implicated for the induction of senescence in some cell types, but the mechanisms by which this might occur remain unexplored^24, 25^. Therefore, specific regulation of the Rb-E2F-p27Kip1-AKT network could be critical for the control of cell proliferation and senescence.

In this study, we determined the molecular mechanism underlying the growth retardation of *Asxl1*-null embryos and derived mouse embryonic fibroblasts (MEFs). We found that ASXL1 interacts with the kinase domain of AKT1 and is required for AKT1 activity. The phosphorylation of p27Kip1 was consistently impaired in *Asxl1* ^−/−^ MEFs. This led to the translocation of p27Kip1 to the nucleus, where it blocked Rb phosphorylation and thereby inhibited E2F activation. In addition, the expression of *p16Ink4a* increased due to defective cooperation with Ezh2 in *Asxl1*-null MEFs. Overall, these data suggest that *Asxl1* plays a critical role in the proliferation of embryonic cells by cooperating with both the AKT-E2F axis and *Ezh2*, the disruption of which leads to senescence.

## Results

### Asxl1 deficiency leads to growth retardation

**Figure 1:**
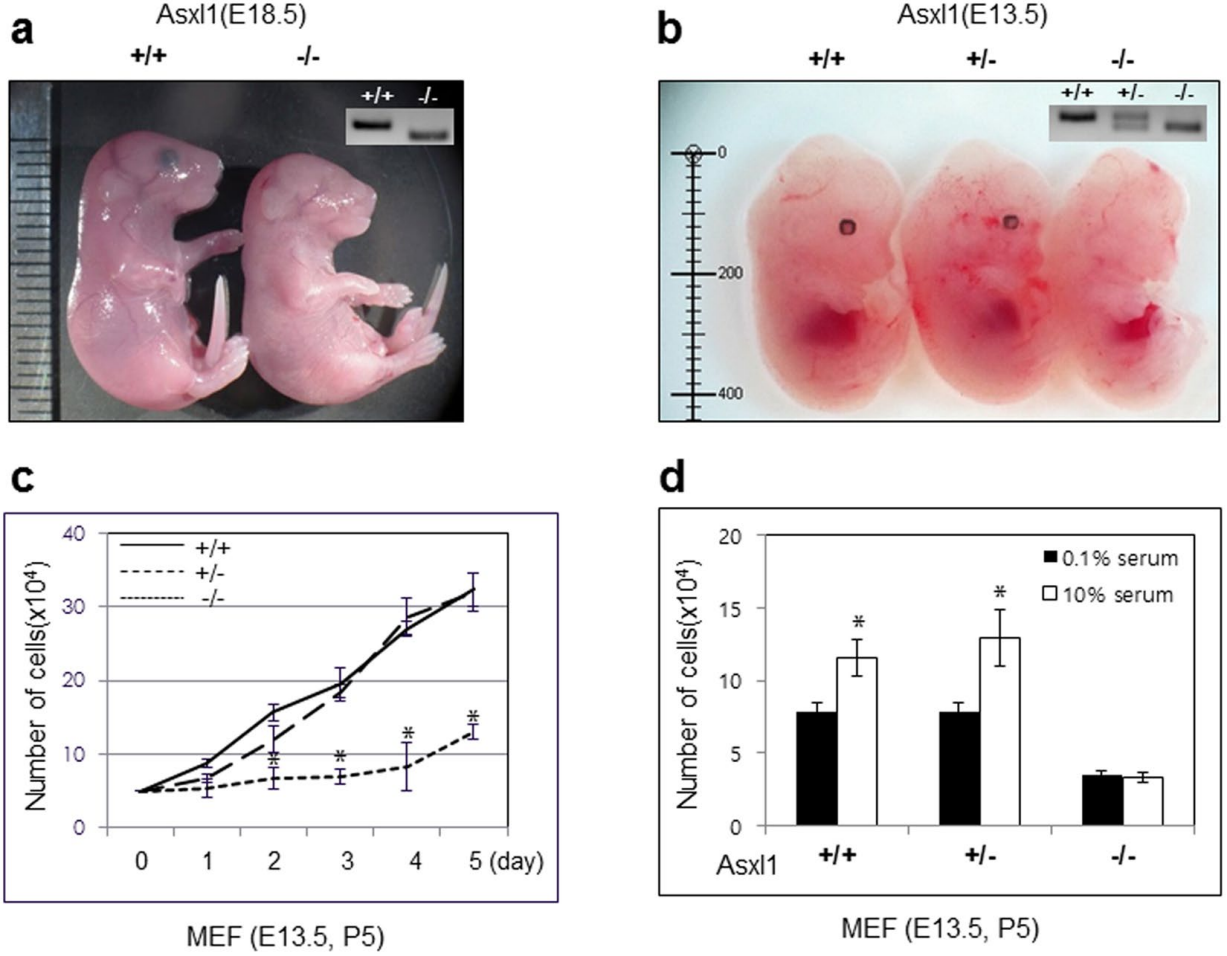
Phenotypic comparison of wild-type and homozygous-*Asxl1* null littermates. (**a**) Wild-type and homozygous-*Asxl1* null embryos at embryonic day E18.5. (**b**) Wild-type (*left*), heterozygous (*middle*), and homozygous-*Asxl1* null embryos (*right*) at embryonic day E13.5. The genotyping results are represented in the upper right corner. (**c**) Proliferation curves of *Asxl1* ^+/+^ (*solid line*), *Asxl1* ^+/−^ (*long dotted line*), and *Asxl1* ^−/−^ (*short dotted line*) MEFs at E13.5. (**d**) Growth curves of *Asxl1* ^−/−^, *Asxl1* ^+/−^, and wild-type MEFs (passage 5) in complete medium with 10.0% and 0.1% FBS. Each experiment was performed in triplicate. Data shown represent the mean ± SD of three independent experiments. Data were subjected to Student’s *t*-test; a significant difference vs. WT-MEFs is indicated as *p < 0.05.

To address the physiological relevance of ASXL1, we generated *Asxl1*-null mice. Homozygous *Asxl1*-deficient mice showed the typical eyeless phenotype, and homozygosity was fatal to all pups in the early postnatal period. Homozygous embryos at E18.5 were significantly smaller and had body weights that were about 80% of their heterozygous or wild-type (WT) littermates (Fig. 1a), suggesting that *Asxl1* disruption may cause developmental defects and growth retardation. To further investigate this finding, we isolated MEFs derived from E13.5 embryos of *Asxl1*-null mice (Fig. 1b). The numbers of MEFs at passage 5 (P5) were counted daily for 5 days after staining with 0.4% trypan blue solution. The number of MEFs from homozygote *Asxl1* ^−/−^ embryos was significantly lower than the number from WT or heterozygote embryos (Fig. 1c). At early passages (less than P3), no growth difference was observed (data not shown). To determine the effect of growth factors on the proliferation of MEFs, cells were cultured with 0.1% and 10.0% serum. The higher serum concentration significantly increased the proliferation of both WT and heterozygote MEFs, but no serum effect was observed in *Asxl1* ^−/−^ MEFs (Fig. 1d). Further FACS analysis indicated that the G0/G1 population was significantly higher in *Asxl1*-null MEFs (67.11%) than in WT (48.94%) and heterozygote (51.33%) MEFs (Supplementary Figure 1). The population of G2/M-phase cells was decreased in *Asxl1*-null MEFs. No induction of apoptosis in these cells was observed (data not shown). Overall, *Asxl1*-deficiency retards cell proliferation by inducing G1 arrest.

### Asxl1 interacts with and activates AKT1

**Figure 2:**
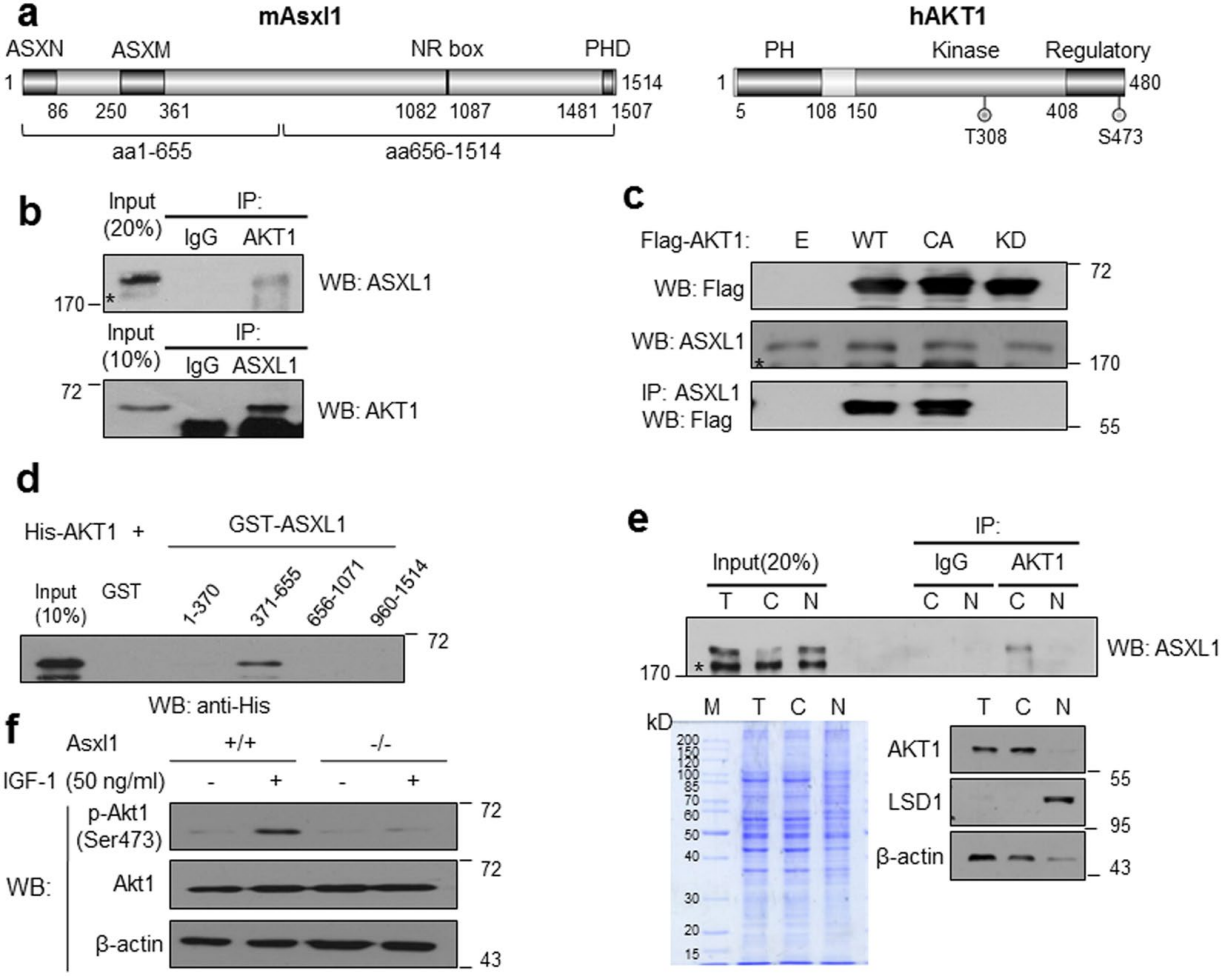
Interaction of ASXL1 with AKT1 and its role in AKT1 activation. (**a**) Schematic representation of mouse Asxl1 and human AKT1. Three conserved ASXN, ASXM, and PHD domains from the ASXL family and NR box (LRMLL) are indicated by closed boxes (*left*). The three conserved PH, kinase, and regulatory domains (closed boxes) among the AKT family and the phosphorylated site are shown (*right*). (**b**) Endogenous interaction between ASXL1 and AKT1. Cell lysates from H1299 cells were subjected to IP using anti-AKT1 or anti-ASXL1 antibody, and the precipitated proteins were visualized by WB with anti-ASXL1 or anti-AKT1 antibody. Star mark indicates non-specific band^42^. (**c**) Effects of kinase activity on the interaction. H1299 cells were transfected with Flag-AKT1, constitutively active (CA), or kinase-deficient (KD) mutant expression vectors, and cell lysates were prepared for IP and WB analysis. (**d**) Mapping of ASXL1 domain responsible for AKT1 binding. GST pull-down assays were performed using purified His-AKT1 and GST-ASXL1 fragments. (**e**) Cytoplasmic interaction between ASXL1 and AKT1. Either nuclear or cytoplasmic fractionation of H1299 cells was determined by WB using indicated antibodies (T, total; C, cytoplasmic; N, nuclear fraction). LSD1 and β-actin serve as controls for nuclear and cytosolic fractions, respectively. (**f**) Effect of *Asxl1* disruption on AKT1 phosphorylation. WB analysis was performed using MEFs from *Asxl1* ^+/+^ or *Asxl1* ^−/−^ mice in the absence or presence of IGF-1 for 30 min and anti-p-Akt1 antibody.

The growth defect of *Asxl1*-null MEFs prompted us to investigate the underlying molecular mechanisms by addressing whether ASXL1 is functionally associated with AKT, a serine/threonine-specific kinase that is known as a key regulator in cell proliferation and tumorigenesis^15, 16^. Mouse Asxl1 is a 1,514-amino-acid (aa) protein composed of four structural regions homologous to the mammalian ASXL family: ASXN, ASXM, NR box, and PHD finger (Fig. 2a, *left*). Human AKT1 comprises 480 aa and possesses 3 regions homologous to the AKT family: the N-terminal PH domain required for binding inositol phospholipids, the kinase domain, and the C-terminal regulatory domain (Fig. 2a, *right*). Immunoprecipitation (IP) with an anti-AKT1 antibody and subsequent western blotting (WB) with an anti-ASXL1 antibody showed endogenous interaction between ASXL1 and AKT1, which was confirmed by reciprocal IP and WB (Fig. 2b). It is well documented that AKT kinase is activated through phosphorylation at Thr308 and Ser473 in response to extracellular stimuli, including growth factors^26^. To further investigate whether the ASXL1-AKT interaction is affected by AKT phosphorylation, we performed IP using three different Flag-tagged AKTs: Flag-WT (wild-type), Flag-CA (constitutive active mutant: T308D/S473D), and Flag-KD (kinase-deficient mutant: T308A/S473A). As shown in Fig. 2c, we found that ASXL1 interacts with the WT and CA mutants, but not with the KD mutant, suggesting that AKT phosphorylation or activation is important for interaction with ASXL1. Subsequent domain mapping by IP assay indicated that the N-terminal half of ASXL1 is sufficient for AKT1 binding (Supplementary Figure 2a). GST pull-down assays using GST-fused ASXL1 and His-fused AKT fragments revealed that the regions of ASXL1 fragment (aa 371–655) and the kinase domain of AKT (aa 125–422) were minimally sufficient for the interaction between ASXL1 and AKT1 (Fig. 2d, Supplementary Figure 2b and c). The interaction with AKT1 was specific for ASXL1, but not for ASXL2 (data not shown). Further IP using cytoplasmic and nuclear fractions indicated that the interaction occurs at the cytoplasm (Fig. 2e). Overall, we determined the physical interaction between ASXL1 and AKT1 and mapped the minimal regions responsible for the interaction.

To address the functional significance of the interaction, we used *Asxl1*-null MEFs to determine the effect of *Asxl1* deletion on the phosphorylation of AKT1. AKT1 phosphorylation at Ser473 was elevated in response to IGF-1 treatment in normal MEFs (Supplementary Figure 2d). However, IGF-1-inducible AKT1 phosphorylation, but not AKT expression, was impaired in *Asxl1*-null MEFs (Fig. 2f). The requirement of AKT phosphorylation for ASXL1 binding was further determined by co-IP upon inducing or inhibiting AKT1 phosphorylation by IGF-1 or LY294002, a selective PI3K inhibitor. As shown in Supplementary Figure 2e, IGF-1 treatment increased the ASXL1 interaction with phosphorylated AKT1, while significant reduction of the interaction was observed by LY294002. This suggests that ASXL1 is critical for AKT1 phosphorylation and activation, likely through direct interaction. Given the role of AKT1 activation in cell proliferation, this finding may explain our early observation that *Asxl1*-null MEFs have defective growth.

### *Asxl1* disruption results in down-regulation of E2F target genes

**Figure 3:**
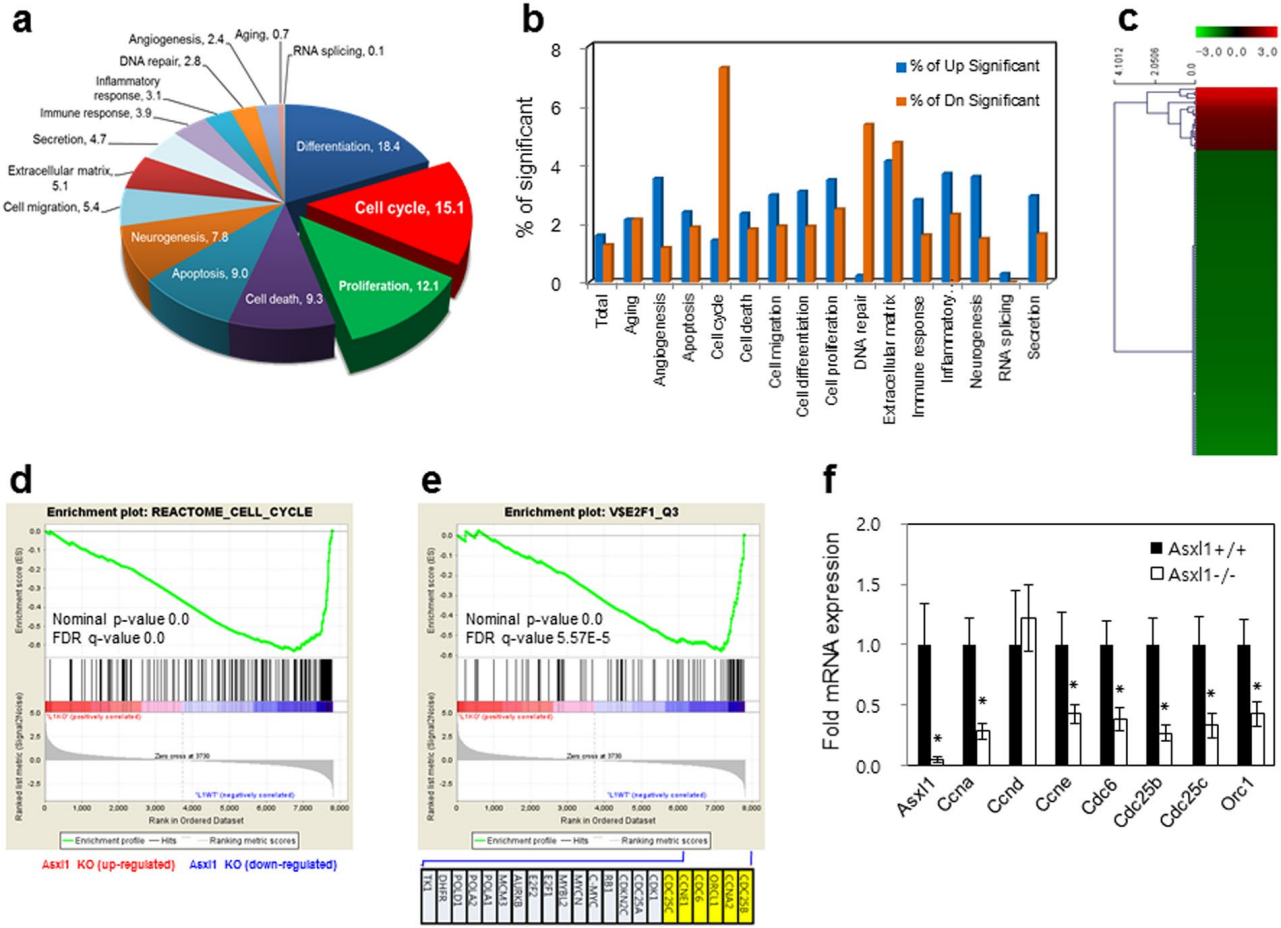
Genome-wide analysis in *Asxl1* ^−/−^ and *Asxl1* ^+/+^ primary MEFs (passage 5). (**a**,**b**) Distribution of functional classification of the 1,128 genes that were significantly higher (≥2-fold) in *Asxl1* ^−/−^ MEFs than in *Asxl1* ^+/+^ MEFs. Pie chart (**a**) and bar graph (**b**) representation of gene ontology for genes differentially expressed in microarray analysis according to biological process. (**c**) Clustering analysis of genes associated with cell cycle. In total, 129 genes were analyzed. (**d**,**e**) Gene set enrichment analysis (GSEA). GSEA was performed by comparing *Asxl1*-responsive genes with two different gene sets associated with the cell cycle process (**d**; REACTOME_CELL_CYCLE) and E2F1 signaling (**e**; V$E2F1_Q3). (**f**) Validation of mRNA expression. Total RNAs were purified and reverse-transcribed. cDNA was amplified by RT-qPCR using specific primer sets for known E2F target genes (Supplementary Table 5). mRNA levels were normalized to endogenous β-actin. Data are shown as mean ± SD of three independent experiments (*p < 0.05).

To investigate how *Asxl1* induces growth retardation when disrupted, we sought to identify genes that are differentially regulated by *Asxl1* disruption. For mRNA preparation, WT and *Asxl1*-null MEFs at passage 5 were cultured for 2 days and then treated with IGF-1 for 30 min. Subsequent microarray analysis identified 1,128 genes, including 628 up-regulated and 500 down-regulated genes, with a greater than 2-fold change in *Asxl1*-null MEFs (Supplementary Table 1). Gene ontology analysis of the regulated genes revealed that a large portion of genes were involved in the differentiation, cell cycle and proliferation (Fig. 3a). Among the 15 categories, cell cycle-related genes in particular were significantly down-regulated (7.3%) in *Asxl1*-null MEFs (Fig. 3b). Further clustering analysis of cell cycle-related genes showed that some of down-regulated genes were E2F target genes (Fig. 3c and Supplementary Table 2). The functional correlation between ASXL1 and E2F was further confirmed by gene set enrichment analysis (GSEA) using the msigdb.v4.0.symbols.gmt gene set file. We found that gene sets associated with cell cycle regulation (REACTOME_CELL_CYCLE) were significantly enriched in a ranked-list of *Asxl1*-regulated genes (Fig. 3d). We also prepared a gene set containing known E2F target genes (V$E2F1_Q3) and performed GSEA analysis (Fig. 3e). Other data sets were also included: CHANG_CYCLING_GENES, BENPORATH_ CYCLING_GENES, MITOTIC_ G1_G1_S_ PHASES, KONG_E2F3_TARGETS, MARSON_BOUND_BY_E2F4_ UNSTIMULATED, and ISHIDA_E2F_TARGETS (Supplementary Figure 3a–f). Together, our GSEA data support our previous results showing that *Asxl1* plays an important role in the expression of E2F target genes in MEFs. To substantiate the array and GSEA data, a subset of these genes was selected and analyzed by RT-qPCR. As shown in Fig. 3f, most of the E2F target genes were significantly down-regulated in *Asxl1*-null MEFs. Interestingly, no expression change of *Ccnd*, another target of E2F, was observed in either microarray or RT-qPCR analysis.

### *Asxl1* deletion induces Rb activation through the down-regulation of p27kip1 phosphorylation

**Figure 4:**
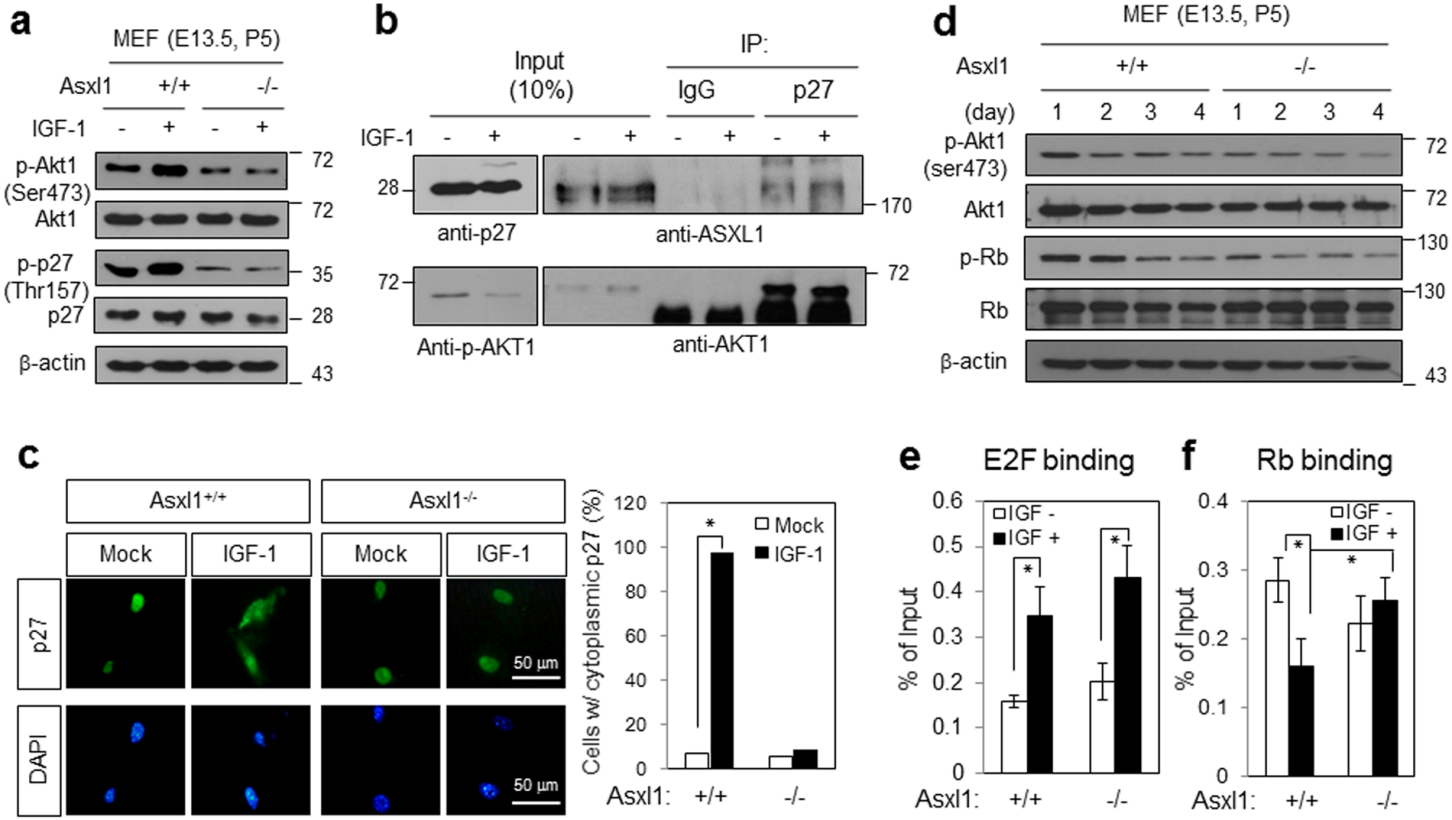
Effect of *Asxl1* disruption on p27Kip1 phosphorylation and Rb activation. (**a**) Hypophosphorylation of p27Kip1 by *Asxl1* disruption. Phosphorylation of p27Kip1 (Thr157) was monitored by WB using an anti-p-p27Kip1 antibody. (**b**) Ternary complex of ASXL1, AKT1, and p27Kip1. Lysates were prepared from HEK293 cells with or without IGF-1 treatment and subjected to IP using an anti-p27 antibody. The precipitated proteins were analyzed by WB using anti-ASXL1 and anti-AKT antibody. (**c**) Effect of *Asxl1* disruption on the subcellular localization of p27Kip1. Fluorescence microscopy was performed with MEFs using primary anti-p27 antibody and Alexa 488-conjugated secondary antibody. Hoechst staining was used to visualize chromosomal DNA. (**d**) Hypo-phosphorylation of Rb. The Rb phosphorylation (Ser807/811) of MEFs (passage 5) from *Asxl1* ^+/+^ or *Asxl1* ^−/−^ mice was monitored daily by WB using an anti-p-Rb antibody. (**e**,**f**) Increased Rb binding to the *Ccna2* promoter upon *Asxl1* deletion. ChIP assays with anti-E2F1 (**e**) or anti-Rb (**f**) antibody and primer set for mouse *Ccna2* promoter (Supplementary Table 6). *Asxl1* ^+/+^ and *Asxl1* ^−/−^ mice-derived MEFs were treated with IGF-1. Chromatin binding was represented as a percentage of input. Data are the mean ± SD of three independent experiments (*p < 0.05).

It has been reported that AKT-mediated p27Kip1 phosphorylation leads to cytoplasmic relocalization of p27Kip1 from the nucleus, thus causing no longer inhibiting CDK2 or CDK4, promoting Rb dephosphorylation and E2F inactivation, and inducing cell cycle arrest at G1^18–21^. In this regard, we investigated whether *Asxl1* deficiency affects the expression and phosphorylation of p27Kip1. Upon IGF-1 treatment, Akt1 phosphorylation was enhanced in WT MEFs, and this was accompanied by a slight elevation in the phosphorylation of p27Kip1 without affecting the levels of Akt1 or p27Kip1. However, a significant decrease in p27Kip1 phosphorylation was observed in *Asxl1*-null MEFs, which was likely due to Akt inactivation (Fig. 4a). This finding prompted us to address whether ASXL1 forms a ternary complex with AKT1 and p27Kip1. As shown by IP assays using HEK293 cells treated with IGF-1, we demonstrated that p27Kip1 interacts with both ASXL1 and AKT1 regardless of IGF-1–induced AKT phosphorylation (Fig. 4b). These experiments were followed by immunofluorescence microscopy to determine the subcellular localization of hypophosphorylated p27Kip1 in *Asxl1*-null MEFs. Consistent with reports shown above, IGF-1 treatment induced the cytoplasmic export of p27Kip1 in normal MEFs, whereas the IGF-1 effect was abolished in *Asxl1*-null MEFs; p27Kip1 was thus retained in the nucleus (Fig. 4c). Consequently, we observed a gradual down-regulation of Rb phosphorylation, but no change in the Rb level, during culture of *Asxl1*-null MEFs (Fig. 4d). Finally, we performed a chromatin IP (ChIP) assay to measure the effect of *Asxl1* deficiency on E2F occupancy on the target promoter. In response to IGF-1, no effect of *Asxl1* was observed on E2F1 binding to the *Ccna2* promoter (Fig. 4e), while the IGF-1–repressed Rb binding was recovered in *Asxl1*-null MEFs after Rb activation (Fig. 4f). Rb activation in *Asxl1*-deficient MEFs was further supported by GSEA analysis of our microarray data using public data sets: MARKEY_RB1_CHRONIC_LOF_UP and _DOWN, CHICAS_RB1_ TARGETS_GROWING, and CHICAS_RB1_TARGETS_SENESCENT (Supplementary Figure 4a–d). Overall, our data suggest that the growth retardation in *Asxl1*-null MEFs may be a result of the sequential events of AKT inactivation, p27Kip1 dephosphorylation, nuclear accumulation of p27Kip1, Rb dephosphorylation and activation, E2F inactivation, and ultimately G1 arrest.

### Cellular senescence was induced in *Asxl1*-null MEFs

**Figure 5:**
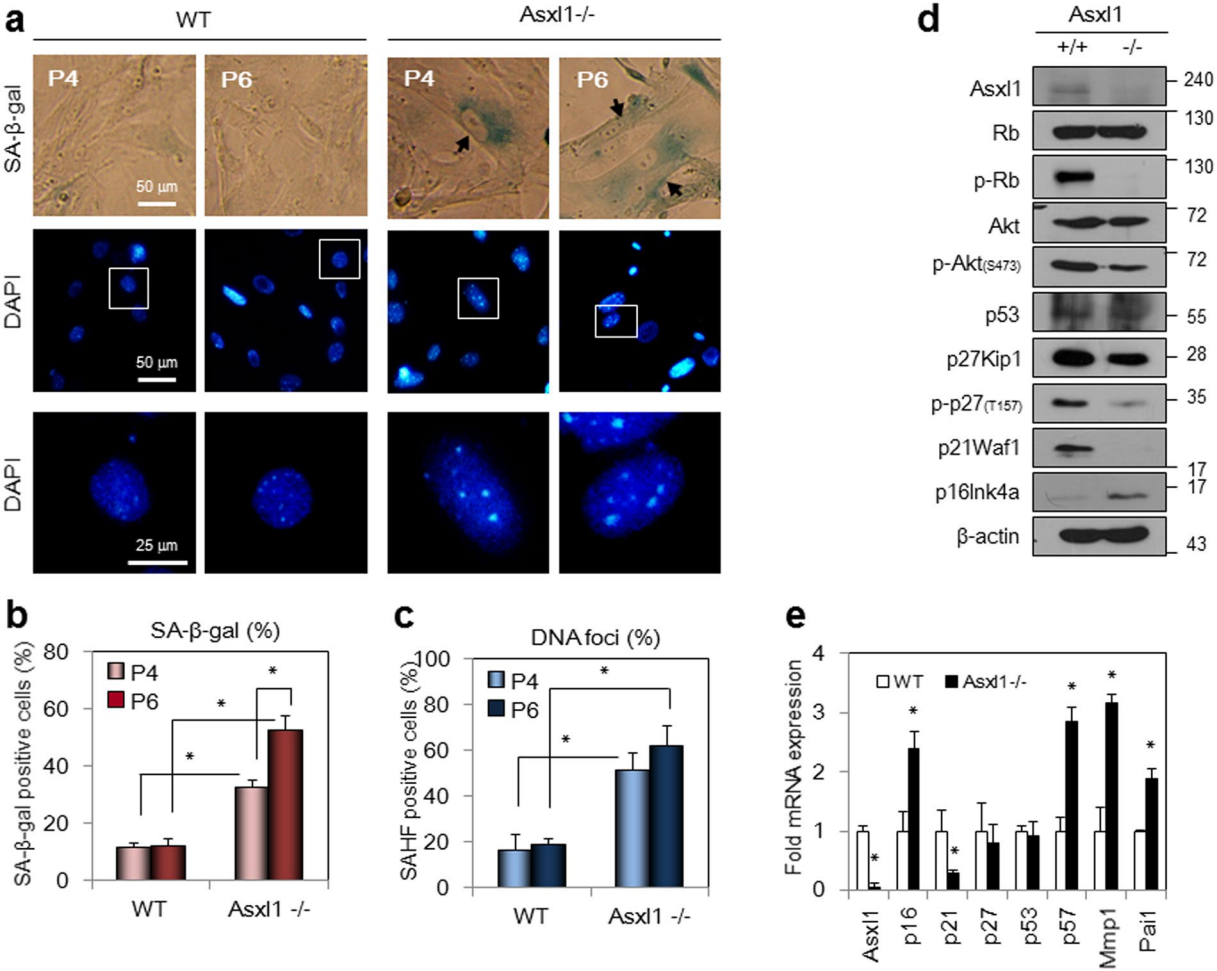
Cellular senescence in *Asxl1*-null MEFs. (**a**) Senescence phenotype determined by SA-β-gal staining and SAHF formation. Wild-type (WT) and *Asxl1*-null MEFs at two different passages (P4 and P6) were stained with SA-β-gal. SAHF formation was determined by DAPI staining. The third row is enlarged representation of the boxes in the second row. (**b**,**c**) Quantitation of SA-β-gal staining and SAHF formation. The numbers of β-gal stained cells (**b**) or cells with heterochromatin foci (**c**) were counted. (**d**) Down-regulation of Rb phosphorylation in *Asxl1*-null MEFs (passage 6). The expression of cell cycle regulators was monitored by WB analysis using the indicated antibodies. (**e**) Up-regulation of *p16Ink4a* in *Asxl1*-null MEFs. Total RNAs were reverse-transcribed and subjected to RT-qPCR using the specific primer sets of indicated genes (Supplementary Table 5). Data are the mean ± SD of three independent experiments (*p < 0.05).

Given that *Asxl1*-null MEFs are halted in G1, we next endeavored to determine the consequences of this phenomenon. Microarray analysis determined that the expression of *p16Ink4a* and *p57Kip2* was increased two-fold in *Asxl1*-deleted MEFs. Because these two CDK inhibitors are known to induce cellular senescence^27, 28^, we measured the effect of *Asxl1* disruption on the induction of senescence using assays for senescence-associated β-galactosidase (SA-β-gal) activity^29^ and formation of senescence-associated heterochromatic foci (SAHF)^30^. SA-β-gal staining was significantly greater in two different passages of *Asxl1*-null MEFs than in WT MEFs (Fig. 5a, *top*). Consistently, more SAHF were formed in *Asxl1*-null MEFs as determined by DAPI staining (Fig. 5a, *middle* and *bottom*). These findings were quantitatively confirmed by counting the number of cells stained with SA-β-gal (Fig. 5b) and DAPI for SAHF formation (Fig. 5c). More senescence was induced at passage 6 of *Asxl1*-null MEFs because of growth retardation in the later stages. In addition, *Asxl1*-deleted MEFs displayed an enlarged and flattened shape (data not shown). The expression and phosphorylation of cell cycle regulators was measured (Fig. 5d). Consistent with the data above (Fig. 4a,d), Rb phosphorylation was significantly decreased accompanying the hypophosphorylation of Akt1 and p27Kip1, while p53 was unaffected in *Asxl1*-null MEFs. Notably, the up-regulation of another Cdk inhibitor, *p16Ink4a* (a hallmark of cellular senescence), was observed in *Asxl1*-null cells, whereas p21Waf1 was down-regulated at the protein and RNA levels (Fig. 5d,e). In addition to *p16Ink4a*, other senescence-associated genes such as *p57Kip2*, *Mmp1*, and *Pai1* were also up-regulated as shown by microarray analysis (Supplementary Table 1) and RT-qPCR (Fig. 5e). Supporting the SAHF data, the expression of heterochromatin-associated markers including HP1γ and tri-methylated histone H3 at lysine 9 (H3K9me3) was evident in *Asxl1*-deleted cells (Supplementary Figure 5). Overall, these findings reveal that *Asxl1* disruption in embryonic fibroblasts induces a senescent phenotype, likely through the activation of Rb, which is caused by hypophosphorylated p27Kip1 and elevated p16Ink4a and p57Kip2.

### AKT inhibitor mimics *Asxl1* deficiency in inducing senescence and nuclear accumulation of p27Kip1

**Figure 6:**
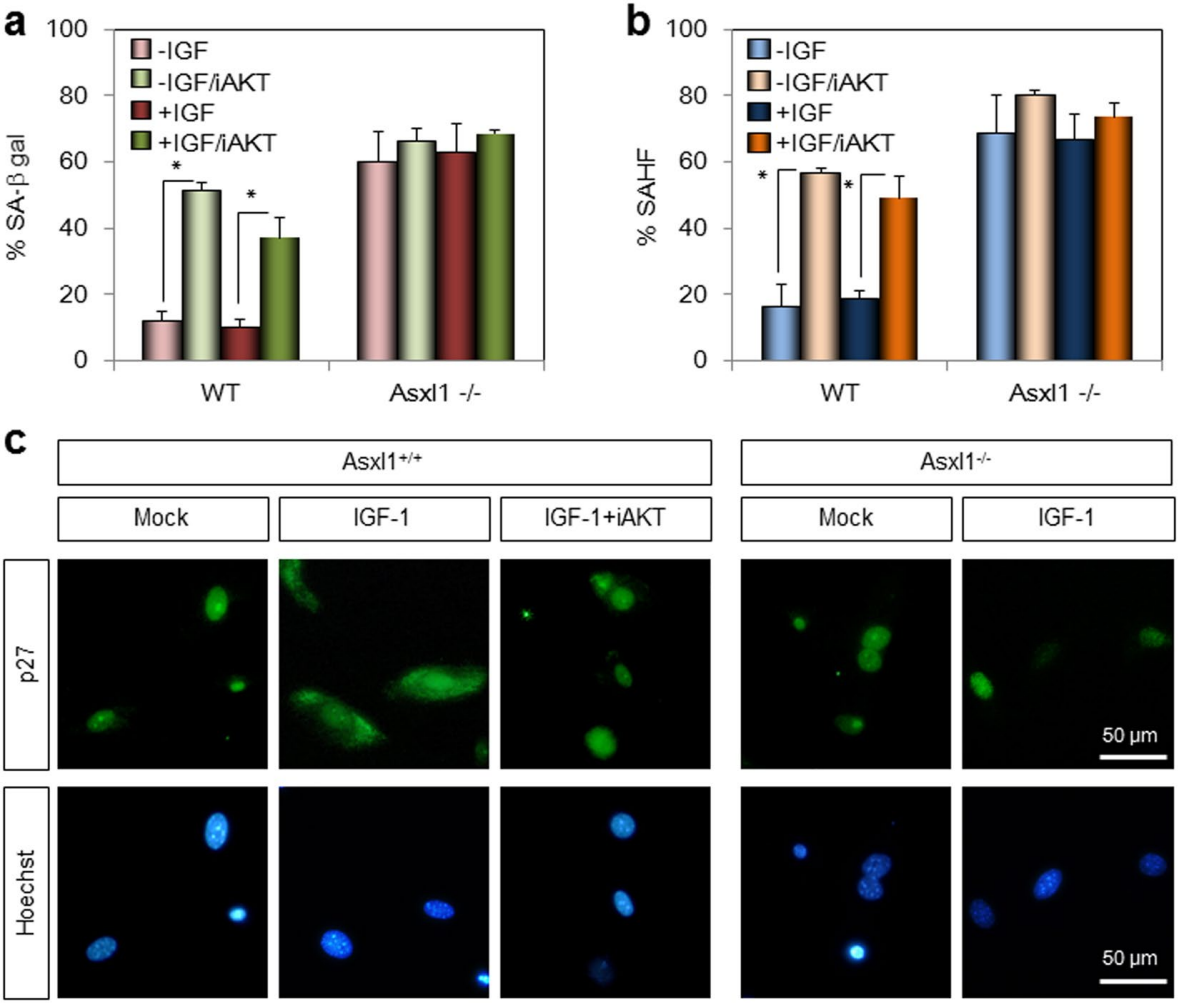
Functional correlation between *Asxl1* disruption and AKT1 inhibition. (**a**,**b**) The induction of senescence by an AKT1 inhibitor. Wild-type (WT) and *Asxl1*-null MEFs were treated with IGF-1 alone or IGF-1 plus AKT inhibitor IV (iAKT) and subjected to senescence assays by SA-β-gal staining and SAHF formation. The numbers of β-gal stained cells (**a**) or cells with heterochromatin foci (**b**) were counted. Data are the mean ± SD of three independent experiments (*p < 0.05). (**c**) The effect of the AKT1 inhibitor on the subcellular localization of p27Kip1. MEFs were treated with IGF-1 alone or IGF-1 plus iAKT and subjected to immunofluorescence microscopy using anti-p27 antibody and Alexa 488-conjugated secondary antibody.

To determine whether the *Asxl1* deletion-induced cellular senescence results from AKT inactivation, we treated normal MEFs with AKT inhibitor IV (iAKT) (Santa Cruz Biotechnology) in the presence of IGF-1 and measured senescence levels. Upon iAKT treatment, the number of SA-β-gal–positive cells was 4-fold higher than untreated normal cells, while no effect of iAKT was observed in *Asxl1*-null MEFs (Fig. 6a and Supplementary Figure 6a). Similar enhancement of SAHF-positive cells was exhibited by iAKT treatment (Fig. 6b). Together with the higher senescent potential of *Asxl1*-deficient cells, these data suggest that AKT inhibitors mimic *Asxl1* deficiency in the induction of senescence. Immunofluorescence microscopy was used further demonstrate that iAKT treatment is correlated with Asxl1 deficiency in the subcellular localization of p27Kip1. As shown in Fig. 6c, the IGF-1-induced cytoplasmic sequestration of p27Kip1 was impaired by iAKT treatment in normal MEFs, which is consistent with the effect of *Asxl1* disruption. The expression and phosphorylation patterns of cell cycle regulators in iAKT-treated cells were also similar to those in *Asxl1*-deleted cells, with the exception of p21Waf1 (Fig. 5d, Supplementary Figure 6b and c). Further comparison by RT-qPCR indicated similar expression patterns of other senescence-associated genes (Supplementary Figure 6c) and various E2F target genes, although *Orc1* was unaffected by iAKT treatment (Supplementary Figure 6d). We also observed senescence and similar expression pattern of genes upon treatment with LY294002, an inhibitor of PI3K (data not shown). Overall, our data support the idea that the effect of *Asxl1* deletion in MEFs on the induction of senescence and nuclear localization of p27Kip1 is caused by AKT inactivation.

### *Asxl1* cooperates with Ezh2 in repressing *p16Ink4a*

**Figure 7:**
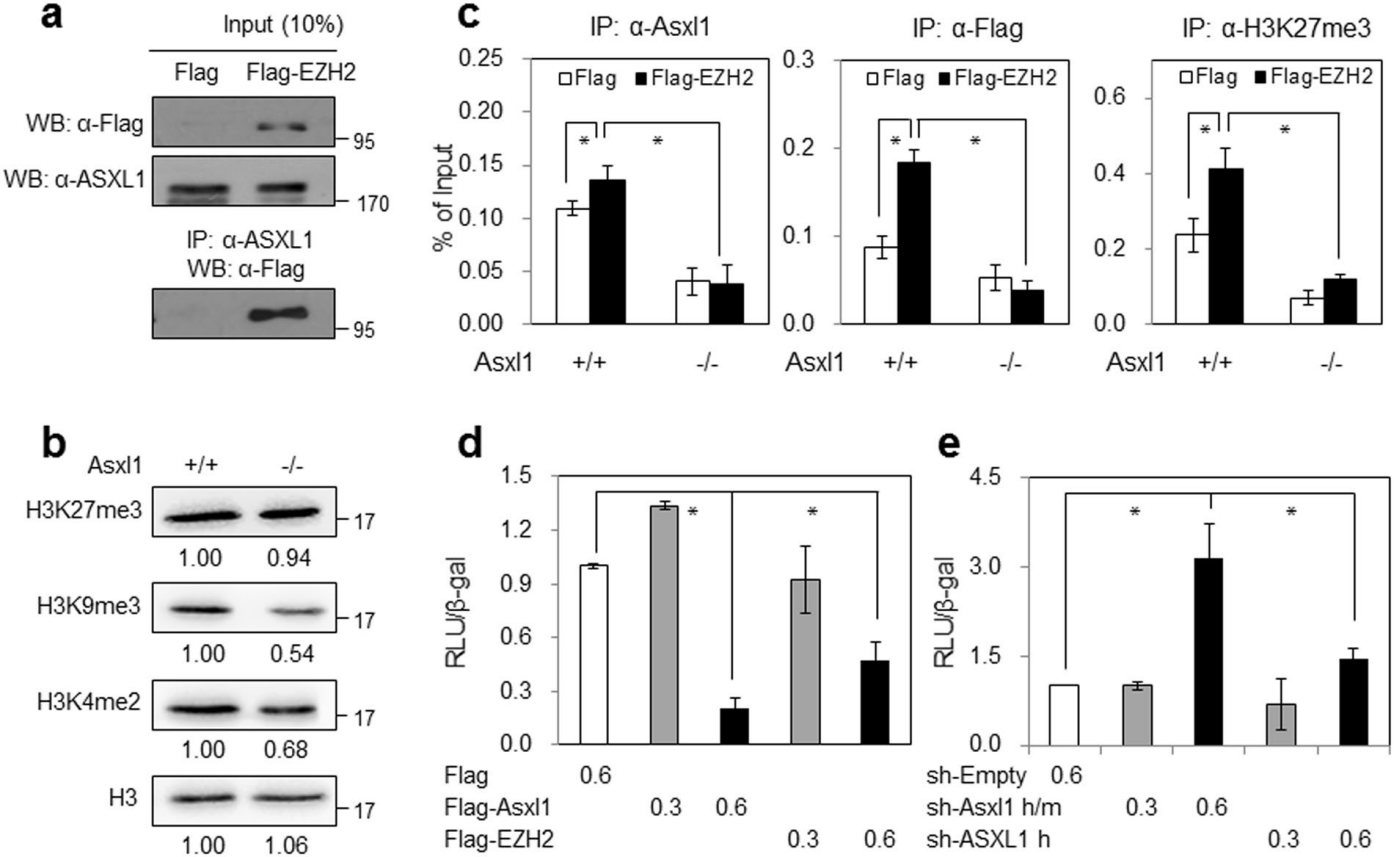
ASXL1 is required for EZH2-mediated *p16Ink4a* repression. (**a**) Physical interaction between ASXL1 and EZH2. HEK293 cells were transfected with Flag-EZH2. Lysates were subjected to IP using an anti-ASXL1 antibody and WB using an anti-Flag antibody. (**b**) Effect of *Asxl1* disruption on the levels of histone H3 methylation determined by WB analysis. (**c**) Requirement of ASXL1 for EZH2 binding to the *p16Ink4a* promoter and its activity therein. ChIP assays were performed using MEFs (either *Asxl1* ^+/+^ or ^−/−^) transfected with Flag-EZH2 and antibodies against ASXL1, Flag, and H3K27me3, and primer set for mouse *p16Ink4a* promoter (Supplementary Table 6). Data are the mean ± SD of three independent experiments (*p < 0.05). (**d**) Effect of *Asxl1* (and EZH2) overexpression on the *p16Ink4a* promoter-driven luciferase (LUC) activity. HEK293 cells were transfected with Flag-ASXL1 or Flag-EZH2 (each 0.3 and 0.6 μg) and *p16Ink4a* promoter-driven luciferase reporter gene. Lysates were subjected to luciferase assays. (**e**) The effect of *Asxl1* knockdown on the *p16Ink4a* promoter activity. For *Asxl1* depletion, HEK293 cells were transfected with two different sets of shRNA (*Asxl1* h/m, common for both human and mouse ASXL1; *Asxl1* h, specific for human ASXL1). Luciferase assays were followed as described above.

To understand why the expression of *p16Ink4a* was elevated in *Asxl1*-null MEFs, we examined a recent report on the physical association of ASXL1 with enhancer of zeste homolog 2 (EZH2)^10^. EZH2 is a component of polycomb repressive complex 2 (PRC2) and functions as a lysine methyltransferase for the tri-methylation of histone H3 at lysine 27 (H3K27me3). In addition, EZH2 is known to repress the expression of *p16INK4a* and *p57KIP2* ^31, 32^. Through microarray analysis and RT-qPCR, we demonstrated that these two genes are up-regulated in *Asxl1*-null MEFs. To explore the link between ASXL1 and EZH2, we first measured the physical interaction by co-IP analysis. As reported previously, Flag-EZH2 interacts with endogenous ASXL1 (Fig. 7a). The link was further supported by GSEA using two public data sets: NUYTTEN_EZH2_TARGETS_ DN (Supplementary Figure 7a) and KONDO_EZH2_TARGETS (Supplementary Figure 7b). No overall change in the endogenous level of H3K27me3 was observed in *Asxl1*-null MEFs compared with the levels of H3K9me3 and H3K4me2 (Fig. 7b). The H3K27me3 level was slightly different in ASXL1-depleted human fibroblast WI-38 cells (Supplementary Figure 7c,d); however, ChIP assays using MEFs (either *Asxl1* ^+/+^ or ^−/−^), transfected with Flag-EZH2, showed that overexpression of Flag-EZH2 resulted in increased recruitment of either ASXL1 or Flag-EZH2 to the *p16Ink4a* promoter, and this recruitment was abolished in *Asxl1*-null MEFs. These results were similar for H3K27me3 (Fig. 7c). Of note, no effect of *Asxl1* disruption on the level of H2AK119ub and its enrichment on the *p16Ink4a* promoter was observed under our experimental conditions (Supplementary Figure 7e). These ChIP data suggest that ASXL1 is required for EZH2 binding to the *p16Ink4a* promoter, where it mediates the tri-methylation of H3K27 and represses the expression of *p16Ink4a*. Subsequent luciferase assays using the mouse p16Ink4a*-*luciferase (LUC) reporter indicated that like EZH2, overexpressed *Asxl1* strongly inhibited luciferase expression (Fig. 7d), while luciferase activity was significantly enhanced upon depleting ASXL1 using two different sets of shRNA (Fig. 7e). The ASXL1 depletion in WI-38 cells also induced a three-fold increase in the mRNA level of *p16INK4A* (Supplementary Figure 7f). Lower levels of senescence were observed in cells overexpressing *Asxl1*, whereas more senescence was induced upon *Asxl1* depletion in WI-38 cells (Supplementary Figure 7g). Together, these data suggest that ASXL1 recruits EZH2 to the *p16INK4a* locus to act as a repressor, which leads to normal cell cycle progression. Reciprocally, ASXL1 defects induce a senescence phenotype through the up-regulation of *p16INK4a* coupled with EZH2 inactivation.

## Discussion

**Figure 8:**
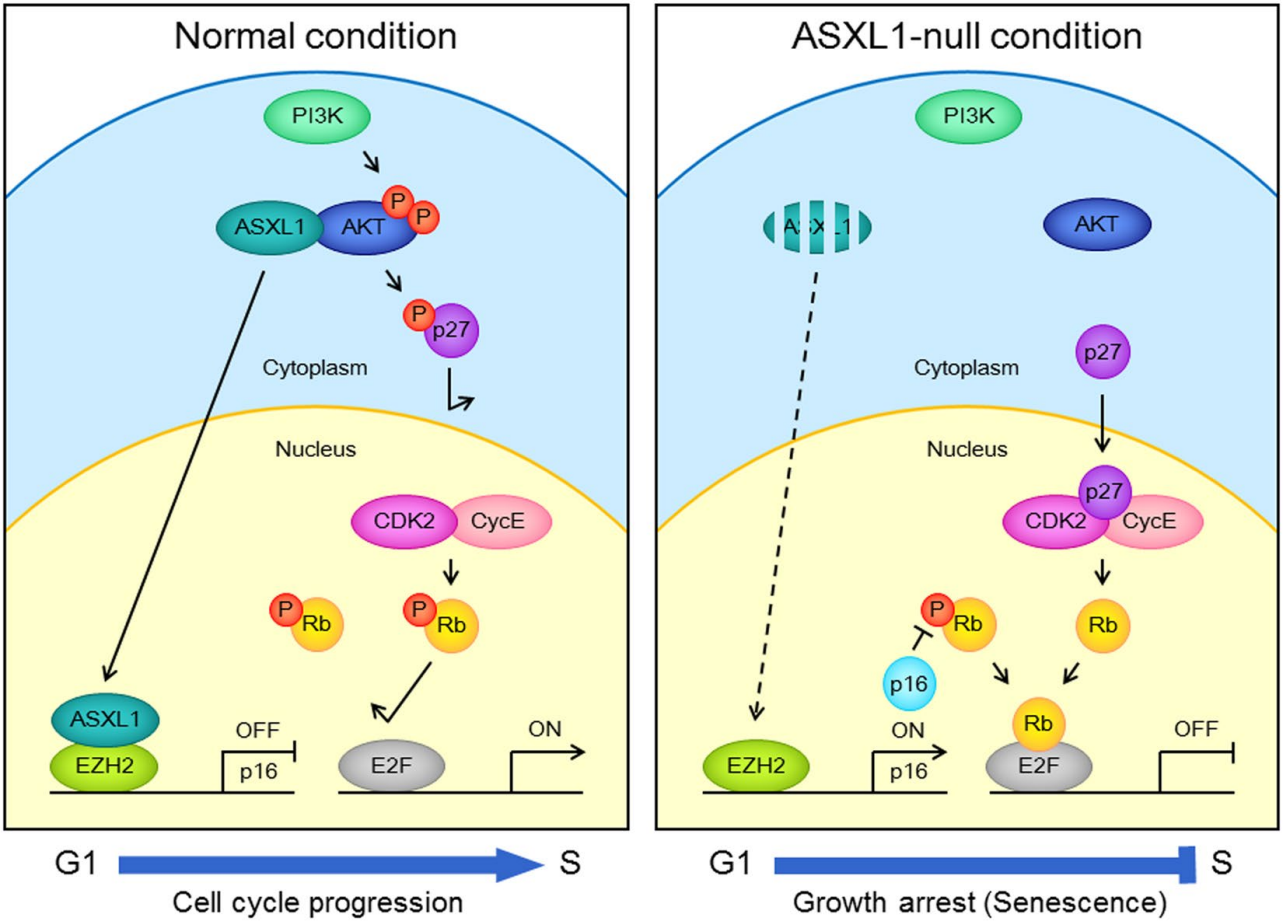
Hypothetical model for the role of ASXL1 in cell cycle progression through the cooperation with AKT1 and EZH2. For details, see the Discussion section.

Mutations of *ASXL1* have been reported in various types of leukemia^7, 8^. Although ASXL1 is considered to be a tumor suppressor, the molecular mechanism underlying tumor suppression is poorly understood. To address the functional role of ASXL1 *in vivo*, we generated *Asxl1*-null mice. Interestingly, homozygous *Asxl1*-null mice were smaller than WT mice, and *Asxl1*-deficient MEFs showed significant growth retardation due to the arrest of cell cycle progression in G1. *Asxl1*-null MEFs did not respond to serum, and this prompted us to investigate the underlying mechanism. We observed that ASXL1 directly interacts with AKT1 kinase and is required for AKT1 activation. This interaction has been suggested from the yeast two-hybrid screening using AKT1 as bait, but the biological significance of the interaction has not been explored^33^. As summarized in Fig. 8, the growth retardation in *Asxl1*-null MEFs partially resulted from the sequential events of AKT1 inactivation, p27Kip1 dephosphorylation, nuclear accumulation of p27Kip1, Rb dephosphorylation, E2F inactivation, down-regulation of E2F target genes, and G1 arrest causing cellular senescence. In addition to AKT1, we also determined that ASXL1 binds to EZH2, a component of the PRC2 complex serving as a histone H3K27 methyltransferase, and cooperates with EZH2 to repress the expression of *p16Ink4a*, a known mediator of cellular senescence. Thus, *Asxl1* disruption additionally leads to EZH2 inactivation, elevated *p16Ink4a* expression and Rb activation, E2F repression, and growth arrest associated with cellular senescence.

The phosphorylation of AKT1 at Ser473 and Thr308 is critical for the activation of AKT1, which is a key regulator of cell proliferation, and is associated with tumorigenesis^15, 16^. In *Asxl1*-null MEFs, AKT1 phosphorylation at Ser473 was significantly impaired even in the presence of the growth factor IGF-1. Furthermore, the growth arrest phenotype and microarray data of *Asxl1*-null MEFs indicated a link between AKT1 and transcription factor E2F. We then speculated that the mediator of the linkage could be p27Kip1, which is a substrate of AKT1^20, 21^. Because AKT1 is a kinase for various substrates including p21Waf1, p27Kip1, MDM2, GSK3β, BAD, and FOXO, we considered how AKT1 could be specific for p27Kip1. This specificity may come from a ternary complex formed by ASXL1, AKT1, and p27Kip1 (Fig. 4b), although the role of ASXL1 for the specificity remains to be determined. Supporting this possibility, our results demonstrated that ASXL1 is required for AKT1 phosphorylation (or activation) and p27Kip1 phosphorylation at Thr157 (Fig. 4a,c). At present, the reason for the slight down-regulation of p27Kip1 at the protein level in *Asxl1*-deleted cells is not known. Subsequently, Rb phosphorylation at Ser807/811 was abolished in these cells, leading to Rb activation. Notably, p21Waf1 is significantly down-regulated at both the RNA and protein levels, suggesting that p27Kip1, not p21Waf1, may mediate Rb activation in *Asxl1*-null cells. Both Rb and p53 are known to promote senescence through p16Ink4a and p14Arf (p19Arf in mice), respectively^22, 23, 34^. Together with the hypophosphorylation of p27Kip1, the up-regulation of *p16Ink4a* and unchanged p53 levels suggest that Rb activation is dominant in mediating senescence in *Asxl1*-deficient MEFs. Consistent with a previous report^30^, we also observed elevated SAHF formation and silencing of E2F target genes in our senescent cells. These findings were repeated after treatment of MEFs with AKT1-specific inhibitor IV (Supplementary Figure 6) and LY294002, an inhibitor of PI3K, an upstream kinase of AKT1 (data not shown), supporting that ASXL1 plays a role in AKT1 activation. However, one exception was the up-regulation of p21Waf1 in drug-treated cells. Although p21Waf1 may contribute to Rb activation, the mechanism of up-regulation during senescence remains to be elucidated.

We previously reported that ASXL1 is mainly located in the nucleus and functions as a transcriptional regulator^5, 6^. In this study, we found that in the cytoplasm, ASXL1 regulates the kinase activity of AKT1 and embryonic stage cell proliferation. We also observed a significant impairment of EZH2 activity for *p16Ink4a* repression due to a defect in ASXL1 cooperation with EZH2 when ASXL1 was absent in embryonic cells. These findings add complexity to the known *ASXL1* functions. In contrast to the positive role of *ASXL1* in the cell cycle progression during early embryogenesis, *ASXL1* has been speculated to be a potential tumor suppressor because of frequent mutations in various myeloid leukemia^7, 8^. Intriguingly, overexpression of *EZH2* has been found in various types of human cancers, including prostate and breast cancer^35, 36^. Similar to *ASXL1*, an inactivating *EZH2* mutation has been reported in poor-prognosis leukemia^37^, whereas a gain-of-function mutation of *EZH2* at Tyr641 in lymphoma is distinct from an *ASXL1* loss-of-function mutation. The mutation-based dual role suggests that *EZH2* may serve as either an oncogene or tumor-suppressor gene depending on the cellular context. Considering this, we propose that *ASXL1* also plays a dual role in regulating cell proliferation and promoting and suppressing cell cycle progression during the embryonic and adult stages, respectively. More studies are required to elucidate the switching mechanism during development. The physical interaction between ASXL1 and EZH2 has been previously reported without addressing its biological significance^10^. Here, we demonstrated the functional link by showing the role of ASXL1 in EZH2-mediated *p16Ink4a* repression and thereby proliferation of embryonic fibroblasts. The molecular and cellular defects identified here will guide future studies of ASXL1 function, particularly in the context of pathological AKT1 activation leading to E2F activation and the functional association with EZH2 during early embryogenesis. Furthermore, the role of *ASXL1* in tumorigenesis will be of interest, and the resulting information may be used for therapeutic interventions of tumors associated with *ASXL1* mutations.

## Methods

We confirm that all methods were performed in accordance with appropriate guidelines and regulations provided by Sejong University. All animal experiments were approved by the Sejong Animal Care Committee.

### Plasmids and cDNA constructions

All cDNA was produced according to standard methods and verified by sequencing. Most of the ASXL1 constructs have been described previously^5, 6^. The desired AKT1 variants were created by PCR amplification using AKT1 cDNA (Sino Biological, North Wales, PA) and subcloned into suitable vectors (Flag (2×)-tagged and Myc-tagged pcDNA3 vectors) for overexpression in mammalian cells. For GST-fused and His-tagged proteins, the pGEX4T-1 (GE Healthcare, Piscataway, NJ) and pET15b (Novagen, Madison, WI) vectors were used. The *p16Ink4a* promoter-driven luciferase reporter was created by inserting the *p16Ink4a* promoter (3,000 bp), amplified by PCR using genomic DNA of mouse tails, into the pGL2 basic vector (Promega, Madison, WI). Details of plasmid constructs are available upon request. For the construction of adenoviruses expressing mAsxl1, the 2xFlag-mAsxl1 region was amplified by PCR and subcloned into the pAdTrack-CMV vector (Addgene, Cambridge, MA) and recombined with the pAdEasy-1 vector (Addgene) in *E*. *coli* BJ5183. The recombinant plasmid was used for rescuing recombinant adenovirus by transfection of QBI-293A cells (MP Biomedicals, Santa Ana, CA).

### Culture of *Asxl1*-null MEFs and other cell lines

Homozygous *Asxl1*-null mice used in these studies were generated by interbreeding heterozygous *Asxl1*-disrupted mice (EM:03996) purchased from the European Mouse Mutant Archive (EMMA, Munich, Germany). Offspring genotypes were determined by PCR analysis using genomic DNA from adult tail tips and embryonic yolk sacs. All animal experiments were performed in accordance with the regulations established by the Korean Council on Animal Care, and all protocols were reviewed by the Sejong Animal Care Committee. MEFs were prepared at embryonic day 13.5 (E13.5) and cultured according to the modified 3T3 protocol^38^. Briefly, MEFs were isolated and cultured in Dulbecco’s modified Eagle’s medium (DMEM) supplemented with 10% fetal bovine serum (FBS), 100 μg/ml penicillin, and 100 μg/ml streptomycin at 37 °C and 5% CO_2_. Prior to IGF-1 treatment (50 ng/ml, 30 min), MEFs were cultured in serum-free low-glucose (1 g/L) DMEM for 24 h. All culture reagents were purchased from Life Technologies (Carlsbad, CA, USA). IGF-1 was purchased from Sigma-Aldrich (St. Louis, MO, USA). In some experiments, WT MEFs were treated with 10 μM AKT inhibitor IV (iAKT) (Santa Cruz Biotech., Santa Cruz, CA) for 1 h. HEK293 and WI-38 cells were grown in DMEM and RPMI 1640 medium, respectively, and supplemented with 10% heat-inactivated FBS and an antibiotic-antimycotic mixture (all from Life Technologies) in a 5% CO_2_ atmosphere at 37 °C.

### Cell proliferation and cell cycle analysis

Cell growth was determined by direct counting of trypan blue-excluded cells. Either WT (Asxl1^+/+^) or mutant (*Asxl1* ^−/−^) MEFs, isolated from E13.5 (passage 5), were seeded in 60-mm culture dishes at a density of 5 × 10^4^ cells per well and cultured for 5 days in media containing 10.0% or 0.1% FBS. Cells were stained with 0.4% trypan blue solution (Merck Millipore, Darmstadt, Germanyand counted daily under a light microscope. The cell cycle profile was assessed by flow cytometry using 1 × 10^6^ cells. Briefly, cells were fixed in 80% ethanol, stained with propidium iodide (PI) solution (Sigma-Aldrich), and analyzed for cell cycle progression using the FACSCalibur Flow cytometer and CellQuest software (BD Biosciences, San Jose, CA).

### RNA interference

The synthetic oligonucleotides used for the depletion of ASXL1 using small hairpin RNA (sh RNA) are shown in Supplementary Table 3. Each duplex was formed and digested with *HindIII* and *BamHI* and ligated with the digested pSilencer 2.1-U6 hygro (Ambion). pSilencer hygro luciferase was used as a control (shLuc). For the knockdown in WI-38 cells, recombinant adenovirus expressing shRNA was prepared. The duplex DNA effective for both human and mouse ASXL1 was digested with *NotI*, and subcloned into the digested PBS/U6 vector (Addgene). The U6 promoter-driven shASXL1 was cut out and ligated with pAdtrack vector (Addgene). The pAdTrack-U6 shASXL1 obtained was recombined with pAdEasy-1 by transformation in *E*. *coli* BJ5183. Recombinant adenovirus was produced by transfecting the recombinant plasmid into QBI-293A cells. Infection and knockdown efficiency was monitored by GFP fluorescence and RT-qPCR, respectively.

### Immunoprecipitation (IP) and Western blotting (WB)

IP and WB were performed as previously reported^6^. MEFs or other cells were lysed in TEN-modified buffer (50 mM Tris-Cl, pH 7.5, 150 mM NaCl, 0.1% Nonidet P-40, 5 mM EDTA, and 1 mM PMSF) supplemented with protease inhibitors (Roche Molecular Biochemicals, Mannheim, Germany). In total, 500 μg and 20 to 50 μg of whole cell lysate was used for IP and WB, respectively. For IP, the lysates were incubated overnight at 4 °C with the indicated antibodies (1:200 dilution). After 2 h of incubation at 4 °C with A/G-agarose beads (Santa Cruz Biotechnology), the beads were washed three times with RIPA buffer. The immune complexes were released from the beads by boiling and analyzed by WB using the indicated antibodies. For WB, lysates or proteins were separated by electrophoresis on 6% to 12% SDS-polyacrylamide gels, transferred to nitrocellulose, and incubated with the primary antibodies as shown in Supplementary Table 4. Concerning ASXL1, our purified anti-ASXL1 antibody was used for IP and commercial anti-ASXL1 antibody (Genetex Inc) was used for WB. The blots were then incubated with peroxidase-conjugated mouse or rabbit IgG secondary antibodies (Santa Cruz Biotechnology). The protein bands were detected using an enhanced chemiluminescence system (GE Healthcare). Protein density was normalized to the β-actin loading control.

### Glutathione S-transferase (GST) pull-down assays

GST-fused ASXL1 and His-tagged AKT1 were expressed in *E*. *coli* and purified on glutathione-Sepharose beads (GE Healthcare) and a HiTrap^TM^ chelating HP column (GE Healthcare), respectively. An approximately equal amount of GST or GST-ASXL1 fragment was mixed with His-tagged AKT1 (and fragments). Bound proteins were detected using WB using anti-His antibody.

### Microarray analysis

Total RNA was extracted from both WT and *Asxl1*-null MEFs cultured for 2 days. Qualified RNA samples, with an RNA integrity number of >9, were used for further analysis in a two-color microarray experiment using mouse 44k 4plex arrays (Agilent, Santa Clara, CA) according to the manufacturer’s instructions. Equal amounts of total RNA were amplified, labeled, hybridized, washed, and scanned. The locally weighted linear regression curve fit and dye-swap normalization methods were applied to the ratio (Cy5/Cy3) of the signal intensities generated in the microarrays. Results were filtered, and the cutoff was set at *p* < 0.05. Genes exhibiting significant differences in expression were classified into gene ontology-based functional categories (http://www.geneontology.org) using the KEGG (http://www.genome.jp/kegg/) and DAVID (http://david.abcc.ncifcrf.gov/) bioinformatics resources.

### Gene set enrichment analysis (GSEA)

GSEA was performed using Java GSEA software v2.0.13 (http://www.broadinstitute.org/gsea). Normalized gene expression profiles were ranked by a signal-to-noise metric, and enrichment scores (ES) were calculated with random gene set permutation 1000. Gene sets were created with genes identified as target genes of the cell cycle, E2F, Rb, senescence, or EZH2. Created gene sets were added to gene set file msigdb.v4.0.symbols.gmt for GSEA. Significance was considered at a nominal p-value (Nom p-value) of <0.05 and a false discovery rate (FDR) of 0.25.

### Reverse transcription and quantitative polymerase chain reaction (RT-qPCR)

Total RNA was extracted from MEFs or WI-38 cells using Isol-RNA lysis reagent (5 PRIME, Gaithersburg, MD) according to the manufacturer’s instructions. One μg of RNA was reverse-transcribed using M-MLV reverse transcriptase and random oligo(dT) primers (Invitrogen). For quantification, real-time PCR reactions were performed using SYBR Green Realtime Master Mix (TOYOBO, Osaka, Japan) and the cycler CFX96 Real-Time PCR detection system (Bio-Rad, Hercules, CA). Cycle threshold (Ct) values were determined in 20 to 35 cycles with primer sets for each gene. All gene expression levels were normalized to *GAPDH* (common for both human and mouse) as an internal standard in each well. Primers used for PCR are shown in Supplementary Table 5. Fold expression was defined as the fold increase relative to controls.

### Chromatin immunoprecipitation (ChIP)

ChIP assays were performed as described previously using indicated antibodies in MEFs^6^. DNA pellets were recovered and analyzed by qPCR using primer pairs (Supplementary Table 6) that encompass E2F1 binding region (−135/+38) on the *Ccna2* promoter^39^ and EZH2 binding site on the *Cdkn2a* (*p16Ink4a*) promoter^40^. Rabbit IgG was used as a negative control. Ratios of fold enrichment from each antibody were calculated from Ct values normalized against Ct of IgG. Percentages of input were calculated and displayed.

### Senescence-associated β-galactosidase (SA-β-gal) assay

The SA-β-gal assay was performed as described elsewhere^41^. Briefly, cells were fixed with 2% formaldehyde and 0.2% glutaraldehyde for 10 min, then incubated with SA-β-gal staining solution (Cell Signaling) at 37 °C for 16 h. The cells were rinsed twice with PBS and methanol before microscopic examination.

### SAHF formation assay

Cells were fixed with ethanol for 1 h, stained with 1 μg/ml of 4′,6-diamidino-2-phenylindole (DAPI; Sigma) at room temperature for 5 min, and observed for SAHF using a fluorescence microscope.

### Immunofluorescence microscopy

WT and *Asxl1*-null MEFs were seeded on cover slides and treated with vehicle (Mock) or 50 ng/ml of IGF-1 for 30 min and/or 10 μM of AKT inhibitor IV for 1 h. MEFs were then washed with PBS, fixed with 3.7% formaldehyde in PBS for 10 min, and permeabilized for 1 h at room temperature in PBS containing 0.5% Triton X-100 and 3% BSA. After washing, the cells were incubated with the indicated primary antibodies in blocking buffer (3% BSA in PBS) for 4 h and incubated with AlexaFluor^®^ 488 goat anti-rabbit IgG or AlexaFluor^®^ 568 goat anti-mouse IgG (Invitrogen). After washing, the cells were visualized under a fluorescence microscope (model DM2500; Leica Microsystems, Wetzlar, Germany). Hoechst (Sigma) staining was used to visualize the chromosomal DNA.

### Transient transfection and luciferase assays

HEK293 cells were seeded on 12-well plates and transfected using Lipofectamine reagent (Invitrogen) with various expression vectors and *p16Iink4a* promoter-driven luciferase reporter as indicated. The CMV-driven-β-galactosidase (gal) expression vector was used as an internal control. After overnight transfection, cells were harvested and subjected to luciferase assay as previously described^6^.

### Statistical analysis

Data are presented as the mean ± standard deviation of at least three independent experiments. Comparisons between multiple groups are presented using paired t-tests. A p value of <0.05 (*) or <0.01 (**) were considered statistically significant.

## Electronic supplementary material

Supplementary data
