# Supplementary material for: Asxl1 deficiency in embryonic fibroblasts leads to cellular senescence via impairment of the AKT-E2F pathway and Ezh2 inactivation
Source: Sci Rep. 2017 Jul 12;7:5198. doi: 10.1038/s41598-017-05564-x (PMC5507898; doi:10.1038/s41598-017-05564-x)

Supplementary information

Asxl1 deficiency in embryonic fibroblasts leads to cellular senescence via impairment of the AKT-E2F pathway and Ezh2 inactivation

HyeSook Youn[1,¶], Tae-Yoon Kim[1,¶], Ui-Hyun Park[1], Seung-Tae Moon[1], So-Jung An[1], Yong-Kyu Lee[1], Jin-Taek Hwang[2], Eun-Joo Kim[3], and Soo-Jong Um[1,*]

*Correspondence: E-mail, umsj@sejong.ac.kr

Supplementary information includes Supplementary figures and legends (S1-S7), Supplementary tables (S1-S6), and original uncropped immunoblots.

Supplementary Figures and legends

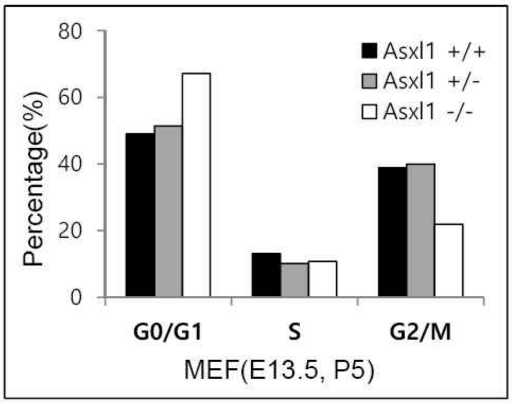

	G0/G1	S	G2/M
**Asxl1 +/+**	48.94	13.02	38.82
**Asxl1 +/-**	51.33	10.1	39.91
**Asxl1 -/-**	67.11	10.77	21.9

Supplementary Figure 1. FACS analysis of *Asxl1*$^{+/+}$, *Asxl1*$^{+/-}$, and *Asxl1*$^{-/-}$ primary MEFs (passage 5).

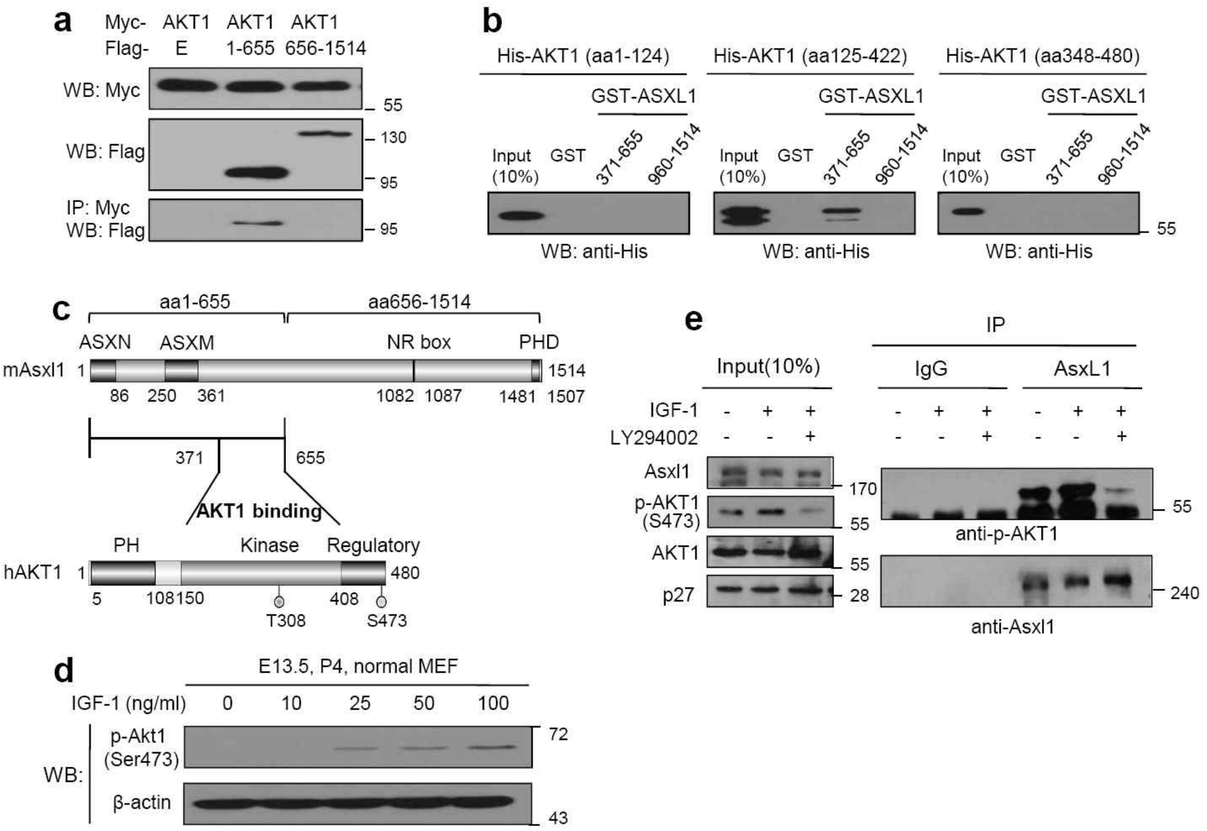

Supplementary Figure 2. Phosphorylation-dependent AKT1 interaction with ASXL1. (**a**) Mapping of the *Asxl1* domain responsible for AKT1 binding. H1299 cells were transfected with Myc-tagged ASXL1 and flag-tagged AKT1 fragments. IP using an anti-Myc antibody was followed by WB using an anti-Flag antibody. (**b**) Further mapping by GST pull-down assays. Direct interaction was verified using purified His-AKT1 fragments and GST-Asxl1 fragments. The interaction was finally visualized by WB using an anti-His antibody. (**c**) Schematic representation of the minimal region required for the interaction between mouse Asxl1 and human AKT1. (**d**) AKT1 phosphorylation at Ser473 in response to IGF-1 in MEFs. Indicated amounts of IGF-1 were treated for 30 min. (**e**) Requirement of AKT1 phosphorylation for ASXL1 binding. H1299 cells were treated with either IGF-1 (50 ng/ml) or PI3K inhibitor LY294002 (50 μM, Cell Signaling). IP using anti-ASXL1 antibody was followed by WB using anti-p-AKT1 and anti-ASXL1 antibodies.

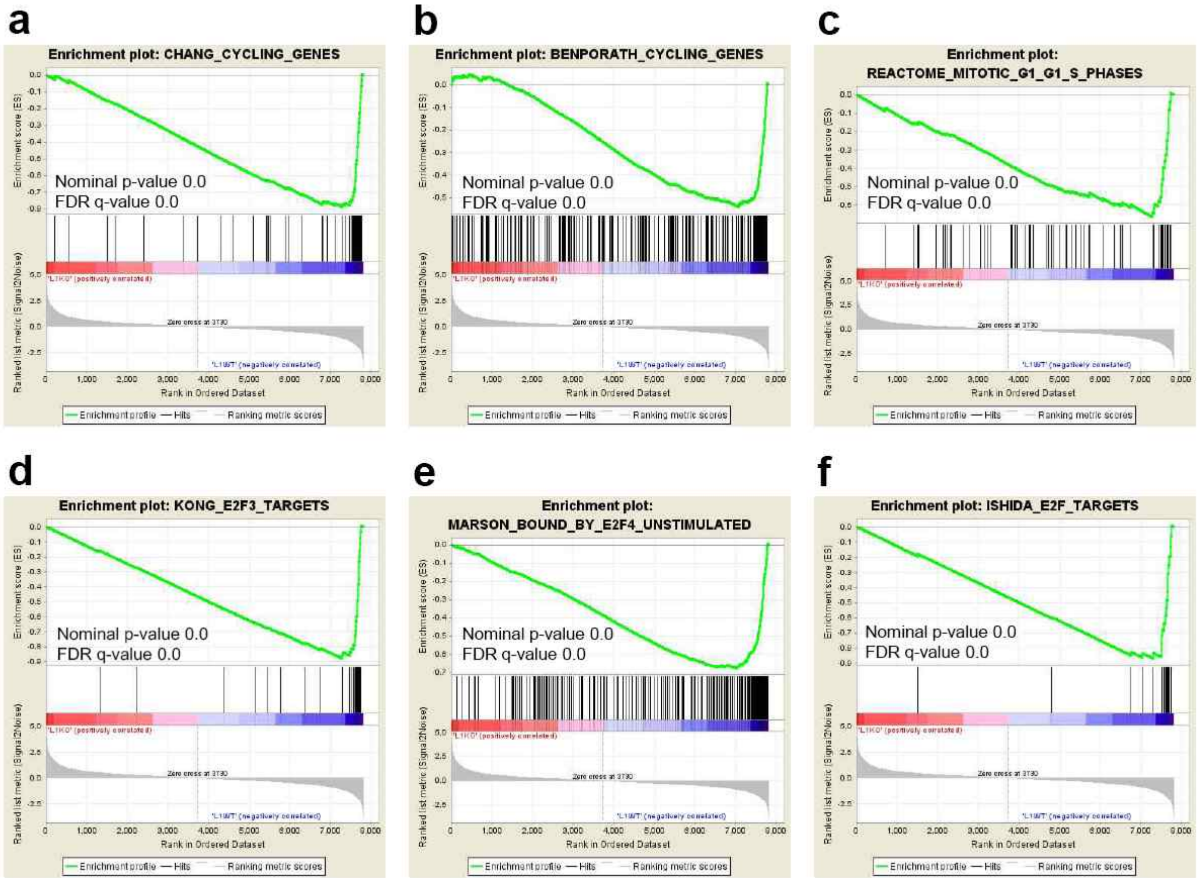

Supplementary Figure 3. Gene set enrichment analysis (GSEA). GSEA was performed by comparing *Asxl1*-responsive genes with six different gene sets associated with the cell cycle process: (**a**) CHANG_CYCLING_GENES, (**b**) BENPORATH_ CYCLING_GENES, (**c**) MITOTIC_ G1_G1_S_ PHASES, (**d**) KONG_E2F3_TARGETS, (**e**) MARSON_BOUND_ BY_E2F4_UNSTIMULATED, and (**f**) ISHIDA_E2F_TARGETS. Both p- and q-values were zero. Red bar indicates genes up-regulated by Asxl1 disruption.

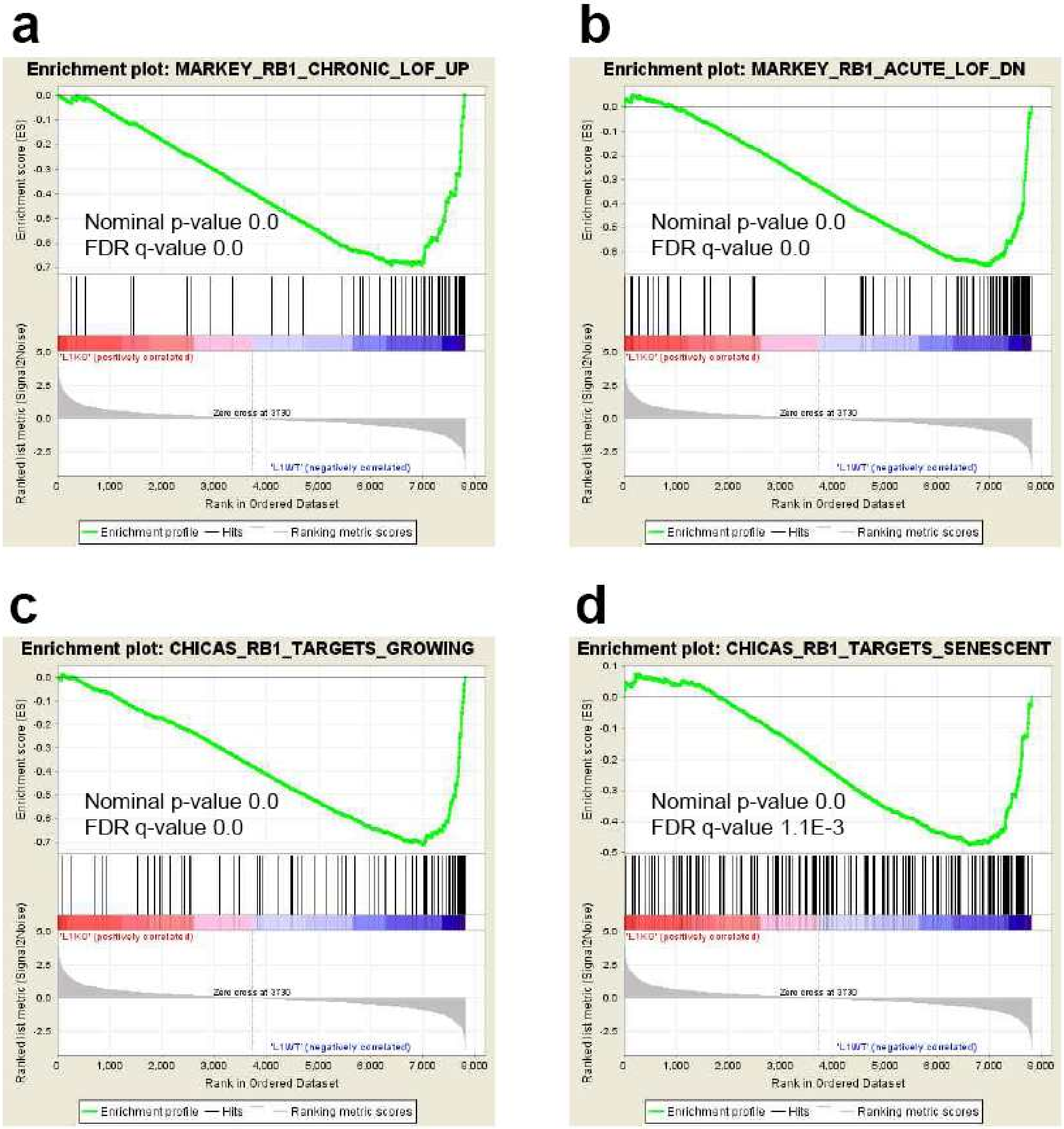

Supplementary Figure 4. Gene set enrichment analysis (GSEA). GSEA was performed by comparing *Asxl1*-responsive genes with four different gene sets associated with Rb signaling: MARKEY_RB1_CHRONIC_LOF_UP (**a**) and _DOWN (**b**), CHICAS_RB1_ TARGETS_ GROWING (**c**), and CHICAS_RB1_TARGETS_SENESCENT (**d**).

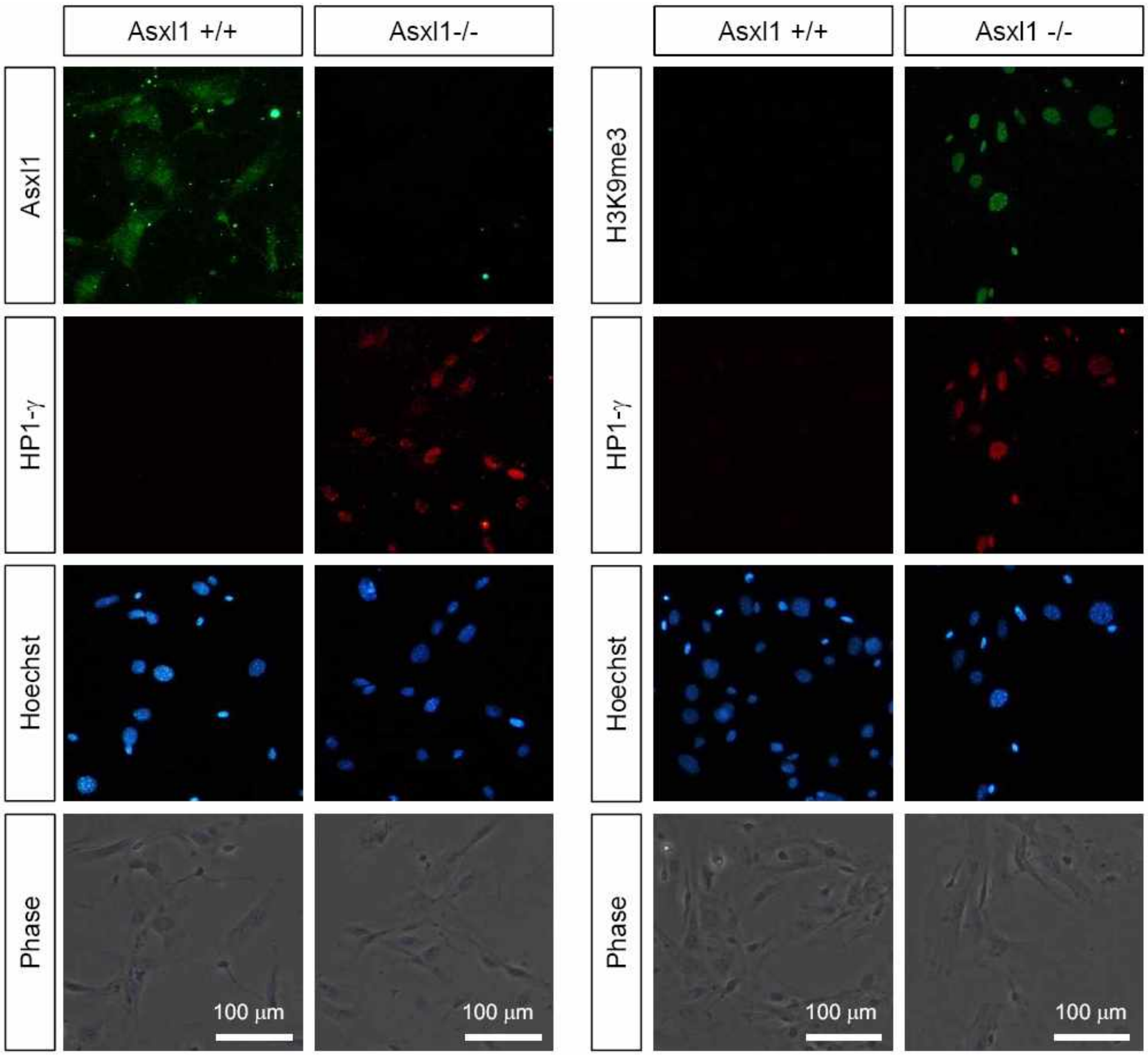

Supplementary Figure 5. Cellular senescence in *Asxl1*-null MEFs. Senescence was monitored by determining the expression of heterochromatin-associated markers including HP1γ and H3K9me3. Immunofluorescence microscopy was performed using antibodies against Asxl1, HP1γ, and H3K9me3. Hoechst staining was used to visualize the nucleus.

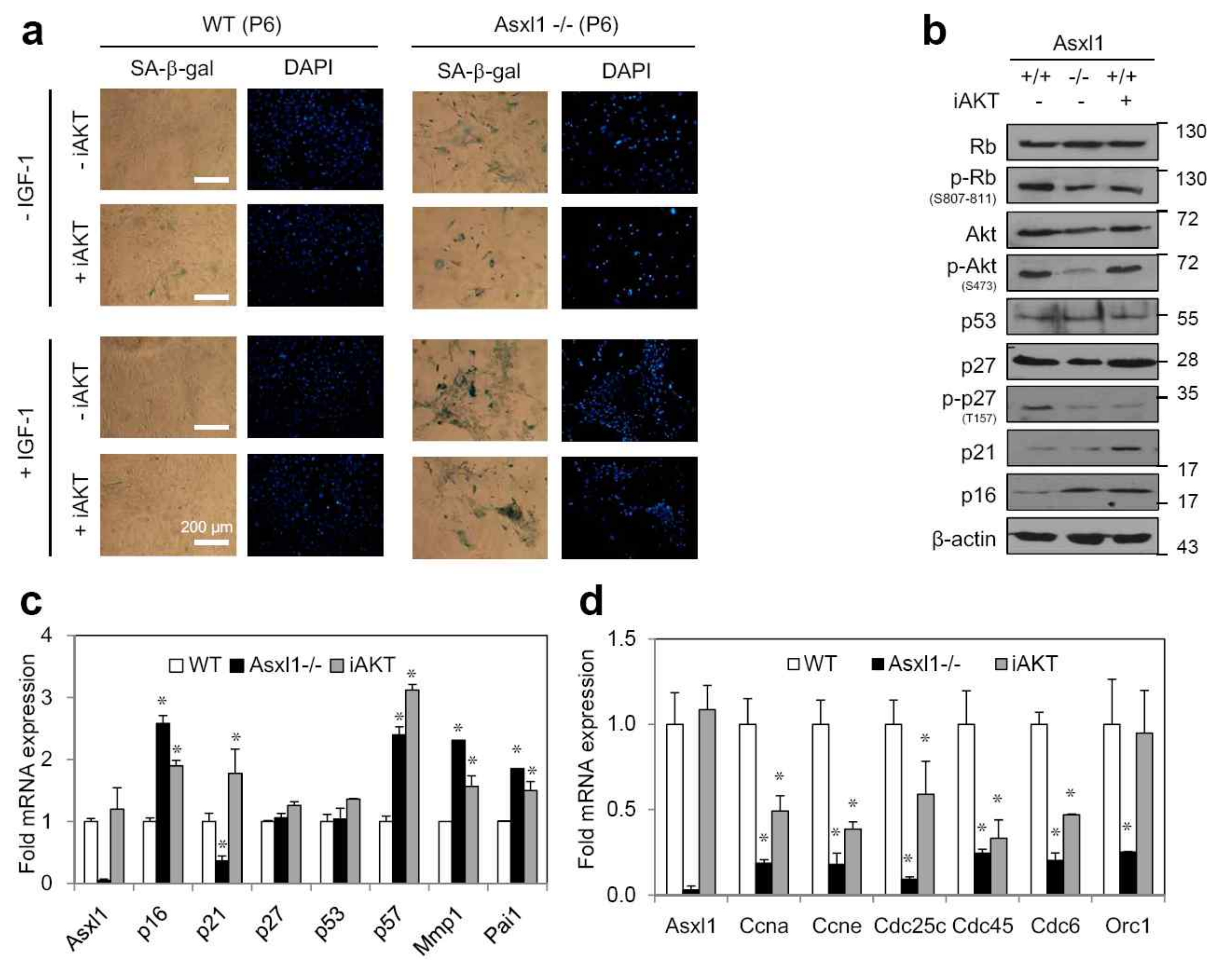

Supplementary Figure 6. Functional correlation between *Asxl1* disruption and AKT1 inhibition. (**a**) The effect of AKT1 inhibitor on senescence. Wild-type (WT) and *Asxl1*-null MEFs were treated with IGF-1 alone or IGF-1 plus AKT inhibitor IV (iAKT) and subjected to SA-β-gal staining. (**b**) Similar expression patterns of cell cycle regulators were found in both *Asxl1*-null MEFs and iAKT-treated MEFs. The expression of cell cycle regulators was monitored by WB analysis using the indicated antibodies. (**c**) The effect of iAKT on the mRNA expression of genes associated with cell cycle and senescence. Total RNAs were reverse-transcribed and subjected to RT-qPCR using the specific primer sets of indicated genes (Table S3). Data are the mean ± SD of three independent experiments (*$p < 0.05$). (**d**) Effect of iAKT on the mRNA expression of known E2F target genes.

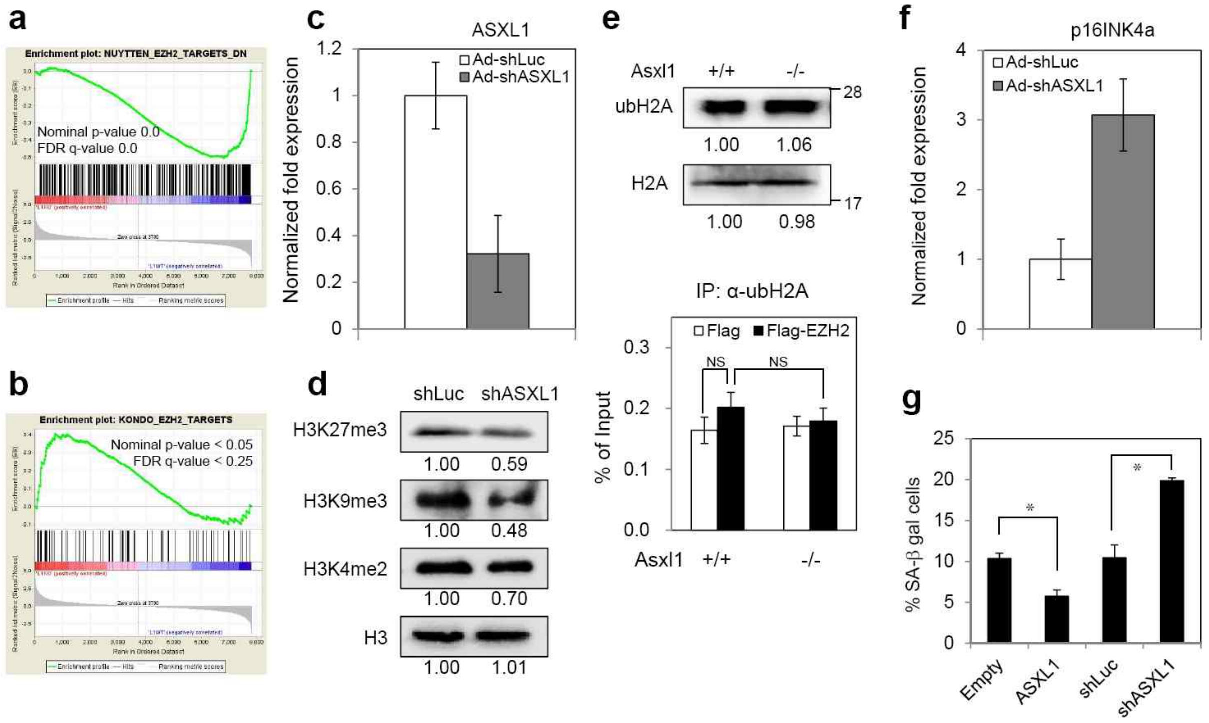

Supplementary Figure 7. ASXL1 requirement for EZH2 function. (**a**, **b**) Functional link between ASXLI1 and EZH2. GESA was performed using our *Asxl1*-regulated gene set and two public data sets: NUYTTEN_EZH2_TARGETS_DN (**a**) and KONDO_EZH2_TARGETS (**b**). (**c**, **d**) Effect of ASXL1 knockdown on the levels of histone H3 methylation. Human fibroblast WI-38 cells were infected with adenovirus (Ad)-driven shASXL1. The mRNA expression of ASXL1 was determined by RT-qPCR (**c**). Tri- or di-methylation of histone H3 at lysine 27, 9, and 4 were compared by WB analysis (**d**). (**e**) Effect of *Asxl1* disruption on histone H2A ubiquitination (upper panel) and enrichment of ubiquitinated H2A at the promoter of *p16INK4a* (bottom panel). Data are the mean ± SD of three independent experiments (NS; not significant). (**f**) Effect of *ASXL1* knockdown on *p16INK4a* expression determined by RT-qPCR in WI-38 cells. (**g**) The effect of *ASXLI1* overexpression and knockdown on inducing senescence in WI-38 cells. Cells were infected with adenovirus-driven ASXL1 or shASXL1 expression vector and subjected to SA-β-gal staining. Stained cells were counted. Data are the mean ± SD of three independent experiments (*p < 0.05).

Supplementary Table 1. List of genes regulated more than 2-fold in *Asxl1*-null MEFs

GeneSymbol	Normalized	Flags	Raw	Control	Genbank	GeneName
Ltb	25.781002	P	242.211	9.394953	NM_008518	lymphotoxin B
5031425F14Rik	25.566288	P	257.403	10.06805	AK147988	RIKEN cDNA 5031425F14 gene
Gpr35	23.520988	P	203.737	8.661923	NM_022320	G protein-coupled receptor 35
LOC100048347	23.046307	P	196.216	8.513976	XM_001480052	similar to TWEAK
Airn	22.008076	P	283.385	12.87639	CB233867	antisense Igf2r RNA
Gabra3	21.940706	P	496.993	22.65164	NM_008067	gamma-aminobutyric acid (GABA) A receptor, subunit alpha 3
Ipw	17.994825	P	284.859	15.83007	NR_015351	imprinted gene in the Prader-Willi syndrome region
Lag3	16.179829	P	314.483	19.43671	NM_008479	lymphocyte-activation gene 3
Olfr1174-ps	15.145614	P	137.802	9.098463	XM_621554	olfactory receptor 1174, pseudogene
Dmgdh	15.023265	P	179.731	11.96354	NM_028772	dimethylglycine dehydrogenase precursor
Gast	13.451111	P	168.865	12.55397	NM_010257	gastrin
Olfr1208	13.101384	P	120.8	9.220392	NM_146778	olfactory receptor 1208
Xlr4b	12.602744	P	226.902	18.0042	NM_021365	X-linked lymphocyte-regulated 4B
Esm1	12.316711	P	757.84	61.52943	NM_023612	endothelial cell-specific molecule 1
Krt6b	12.286055	P	139.673	11.36845	NM_010669	keratin 6B
	12.152805	P	354.827	29.19713	AK135041	
Prl2c5	12.115246	P	3306.91	272.9542	NM_181852	prolactin family 2, subfamily c, member 5
Vmn2r78	12.013048	P	410.148	34.14188	NM_001105189	vomeronasal 2, receptor 78
Acoxl	11.999416	P	145.761	12.14734	NM_028765	acyl-Coenzyme A oxidase-like
Scn1a	11.126107	P	103.607	9.312053	NM_018733	sodium channel, voltage-gated, type I, alpha
Prl2c5	10.839179	P	2230.23	205.7563	NM_181852	prolactin family 2, subfamily c, member 5
EG622110	10.629274	P	240.024	22.58139	XM_922665	predicted gene, EG622110
Pira2	10.549839	P	204.074	19.34382	NM_011089	paired-Ig-like receptor A2
1700121C10Rik	10.09392	P	131.596	13.03718	XM_001477748	RIKEN cDNA 1700121C10 gene
B230319C09Rik	9.446559	P	108.948	11.53307	NR_028382	RIKEN cDNA B230319C09 gene
Gm2789	8.639644	P	165.595	19.16687	XM_001474724	predicted gene 2789

Upk1b	8.255477	P	265.592	32.17161	NM_178924	uroplakin 1B
Dsp	8.048129	P	203.015	25.22508	NM_023842	desmoplakin
AW987390	7.9844456	P	76.8214	9.621385	BQ938751	expressed sequence AW987390
Perp	7.9542994	P	8045.56	1011.473	NM_022032	PERP, TP53 apoptosis effector
Gm3414	7.86965	P	68.6396	8.722066	NR_027993	predicted gene 3414
Arg1	7.857842	P	241.802	30.77211	NM_007482	arginase, liver
	7.790387	P	63.301	8.12552	XM_136914	
Syne1	7.733745	P	67.1743	8.685865	NM_153399	synaptic nuclear envelope 1
Xlr4a	7.630518	P	385.385	50.50571	NM_001081642	X-linked lymphocyte-regulated 4A
Serpinb6b	7.524929	P	1071.36	142.3752	NM_011454	serine (or cysteine) peptidase inhibitor, clade B, member 6b
Sly	7.4117513	P	105.686	14.25927	NM_201530	Sycp3 like Y-linked
Emcn	7.398815	P	194.93	26.34605	NM_001163522	endomucin
Wt1	7.3548427	P	184.257	25.05244	NM_144783	Wilms tumor 1 homolog
LOC100048240	7.1235704	P	70.1691	9.850266	XM_001479834	hypothetical protein LOC100048240
Aldh1a7	7.0823874	P	1426.66	201.4379	NM_011921	aldehyde dehydrogenase family 1, subfamily A7
Rims3	6.9886675	P	64.8117	9.273827	NM_182929	regulating synaptic membrane exocytosis 3
Olfr485	6.900747	P	53.525	7.756403	NM_001011810	olfactory receptor 485
2810006K23Rik	6.8761744	P	139.185	20.24163	NM_028310	RIKEN cDNA 2810006K23 gene
	6.8192563	P	102.615	15.04781	AK013707	
Zfp423	6.726258	P	58.4141	8.68449	NM_033327	zinc finger protein 423
Gm10639	6.70602	P	180.177	26.86801	NM_001122660	predicted gene 10639
Samd11	6.589624	P	70.3239	10.67191	NM_001110516	sterile alpha motif domain containing 11
	6.581202	P	128.72	19.5588	AK154175	
Gm8103	6.5791397	P	201.6	30.64231	XM_983830	predicted gene 8103
Il17a	6.5607953	P	62.5805	9.53856	NM_010552	interleukin 17A
Xlr3b	6.43332	P	911.159	141.6313	NM_001081643	X-linked lymphocyte-regulated 3B
	6.170437	P	96.8574	15.697	AK016064	
Sin3b	6.144359	P	93.1009	15.15225	NM_009188	transcriptional regulator, SIN3B (yeast)
Grm6	6.136856	P	98.5727	16.06241	NM_173372	glutamate receptor, metabotropic 6

3830417A13Rik	6.081677	P	632.677	104.03	NM_027512	RIKEN cDNA 3830417A13 gene
Stmn2	6.0309877	P	2056.56	340.9987	NM_025285	stathmin-like 2
Gm6687	5.8421035	P	51.8582	8.87663	XM_992478	predicted gene 6687
Gm5066	5.7687254	P	191.51	33.19796	XR_034763	predicted gene 5066
Pgk2	5.648807	P	203.028	35.94178	NM_031190	phosphoglycerate kinase 2
	5.630248	P	89.0634	15.81874	AK135819	
Prss47	5.605862	P	79.0629	14.10361	XM_905472	protease, serine, 47
Nkx2-4	5.5797396	P	77.4206	13.87531	NM_023504	NK2 transcription factor related, locus 4 (Drosophila)
Zfp318	5.5763025	P	68.6109	12.30402	NM_021346	zinc finger protein 318
Spaca1	5.56877	P	110.314	19.80931	NM_026293	sperm acrosome associated 1
Efemp1	5.48408	P	18587.4	3389.34	NM_146015	epidermal growth factor-containing fibulin-like extracellular matrix protein 1
AY074887	5.474023	P	203.522	37.17966	NM_145229	cDNA sequence AY074887
Gm1693	5.4545927	P	127.012	23.28531	XM_358673	predicted gene 1693
Gsta2	5.4527698	P	96.544	17.7055	NM_008182	glutathione S-transferase, alpha 2 (Yc2)
Xlr3b	5.441615	P	371.717	68.31009	NM_001081643	X-linked lymphocyte-regulated 3B
Gm16430	5.4399505	P	119.032	21.88108	NM_001166601	predicted gene 16430
Insl5	5.436879	P	92.8943	17.08596	NM_011831	insulin-like 5
Col10a1	5.377234	P	1019.15	189.5306	NM_009925	collagen, type X, alpha 1
Gm16386	5.2929587	P	43.3461	8.189389	NR_030709	predicted gene 16386
Vip	5.280291	P	80.3935	15.2252	NM_011702	vasoactive intestinal polypeptide
2210011G09Rik	5.202567	P	54.1299	10.40446	BG970498	RIKEN cDNA 2210011G09 gene
Synpo2	5.183971	P	102.333	19.74027	NM_080451	synaptopodin 2
Aqp4	5.139131	P	182.55	35.52159	NM_009700	aquaporin 4
Foxa1	5.0940166	P	41.8755	8.220532	NM_008259	forkhead box A1
Dcaf12l1	5.051385	P	1225.06	242.5189	NM_178739	DDB1 and CUL4 associated factor 12-like 1
Adamtsl2	5.0035734	P	145.857	29.15047	NM_029981	ADAMTS-like 2
Uts2d	4.997962	P	595.904	119.2293	NM_198166	urotensin 2 domain containing
Bcl2l15	4.976175	P	374.685	75.29575	NM_001142959	BCLl2-like 15
Slc45a1	4.9072857	P	82.6458	16.84145	NM_173774	solute carrier family 45, member 1

	4.831133	P	74.7215	15.46666	AK156355	
Kcne3	4.821381	P	36.6643	7.604524	NM_020574	potassium voltage-gated channel, Isk-related subfamily, gene 3
Tifab	4.765879	P	106.633	22.37419	NM_145976	TRAF-interacting protein with forkhead-associated domain, family member B
Xlr4a	4.750074	P	50.4481	10.62049	NM_001081642	X-linked lymphocyte-regulated 4A
Gda	4.745912	P	470.259	99.08709	NM_010266	guanine deaminase
6530409C15Rik	4.7276616	P	59.052	12.49074	XM_987873	RIKEN cDNA 6530409C15 gene
Fgf12	4.7055764	P	49.2066	10.45708	NM_183064	fibroblast growth factor 12
Olfr1040	4.700427	P	95.3193	20.27887	NM_207561	olfactory receptor 1040
Foxd2	4.6929693	P	143.001	30.4713	NM_008593	forkhead box D2
Gm2095	4.6672835	P	139.328	29.85214	XM_001472437	predicted gene 2095
A830035A12Rik	4.651613	P	55.5731	11.94705	XM_986896	RIKEN cDNA A830035A12 gene
Il6	4.649089	P	718.84	154.6195	NM_031168	interleukin 6
Olfr424	4.643803	P	46.1384	9.935473	NM_146721	olfactory receptor 424
Akap5	4.640099	P	80.5108	17.3511	NM_001101471	A kinase (PRKA) anchor protein 5
Rarb	4.5240917	P	199.29	44.05085	NM_011243	retinoic acid receptor, beta
	4.4859533	P	127.868	28.50404	AK137609	
Olfr5	4.475047	P	106.727	23.84934	NM_146914	olfactory receptor 5
Olfr1179	4.4728346	P	117.375	26.24177	NM_146917	olfactory receptor 1179
Gsta1	4.455269	P	173.729	38.9941	NM_008181	glutathione S-transferase, alpha 1 (Ya)
Gm16430	4.435502	P	124.444	28.05637	NM_001166601	predicted gene 16430
Prl7b1	4.431864	P	45.1474	10.18701	NM_029355	prolactin family 7, subfamily b, member 1
Gdf6	4.431465	P	194.014	43.78089	NM_013526	growth differentiation factor 6
Zfp551	4.422733	P	58.0804	13.13224	NM_001033820	zinc fingr protein 551
Nkain4	4.376124	P	1468.53	335.5778	NM_021426	Na+/K+ transporting ATPase interacting 4
Gm2899	4.3373895	P	76.4578	17.62761	XM_001475058	predicted gene 2899
3830403N18Rik	4.3360877	P	75.2966	17.36511	NM_027510	RIKEN cDNA 3830403N18 gene
Oprl1	4.3192253	P	41.9482	9.711982	NM_011012	opioid receptor-like 1
Il7	4.2915187	P	86.2693	20.10227	NM_008371	interleukin 7
Sprr2g	4.289707	P	158.784	37.01506	NR_003548	small proline-rich protein 2G

LOC620306	4.26159	P	78.7193	18.47182	AK041010	similar to Glyceraldehyde-3-phosphate dehydrogenase (GAPDH)
Slc5a7	4.256947	P	1462.8	343.6275	NM_022025	solute carrier family 5 (choline transporter), member 7
Clec2h	4.207599	P	93.6982	22.2688	NM_053165	C-type lectin domain family 2, member h
Cpb1	4.2016163	P	55.7069	13.25845	NM_029706	carboxypeptidase B1 (tissue)
Gm11567	4.1954017	P	126.333	30.11225	NM_001101613	predicted gene 11567
Npy1r	4.187831	P	588.11	140.4331	NM_010934	neuropeptide Y receptor Y1
Iws1	4.163533	P	171.46	41.18147	NM_173441	IWS1 homolog (S. cerevisiae)
Trfr2	4.1531997	P	113.635	27.36081	NM_015799	transferrin receptor 2
Iqcd	4.1360793	P	65.2881	15.78502	NM_029408	IQ motif containing D
Olfr1382	4.1328273	P	220.807	53.42757	NM_001011790	olfactory receptor 1382
	4.1291227	P	47.8928	11.59878	AK136394	
Pdk4	4.112628	P	593.993	144.4315	NM_013743	pyruvate dehydrogenase kinase, isoenzyme 4
Prlr	4.097759	P	42.7799	10.43983	NM_011169	prolactin receptor
Mfap5	4.095022	P	3794.26	926.5546	NM_015776	microfibrillar associated protein 5
4930566D17Rik	4.0481973	P	78.5235	19.39716	AK016233	RIKEN cDNA 4930566D17 gene
Prl3d2	4.0149603	P	304.106	75.74326	NM_172155	prolactin family 3, subfamily d, member 1
Vps4b	3.977567	P	187.248	47.07599	AK087331	vacuolar protein sorting 4b (yeast)
Samd12	3.9756215	P	95.6432	24.05743	NM_177225	sterile alpha motif domain containing 12
Adig	3.971278	P	160.225	40.34596	NM_145635	adipogenin
Gm2419	3.9688826	P	83.2042	20.96413	XM_001473482	predicted gene 2419
Cdk7	3.9255407	P	100.867	25.69509	NM_009874	cyclin-dependent kinase 7
Vnn1	3.918478	P	125.295	31.97542	NM_011704	vanin 1
Aldh1a1	3.9148269	P	727.417	185.8109	NM_013467	aldehyde dehydrogenase family 1, subfamily A1
Slitrk5	3.9099145	P	278.957	71.34606	NM_198865	SLIT and NTRK-like family, member 5
Thsd7a	3.8909323	P	58.0611	14.92216	NM_001164805	thrombospondin, type I, domain containing 7A
Sytl1	3.8847816	P	867.57	223.3253	NM_031393	synaptotagmin-like 1
	3.8821132	P	58.8554	15.16067	AK144145	
Thsd7a	3.8811526	P	1131.83	291.6219	NM_001164805	thrombospondin, type I, domain containing 7A
Gm3459	3.8803601	P	953.46	245.7144	XM_001476778	predicted gene 3459

	3.8758614	P	37.2309	9.605825	AK136613	
Angpt1	3.8736188	P	37.4672	9.672392	NM_009640	angiopoietin 1
Camk4	3.861839	P	62.4411	16.16873	NM_009793	calcium/calmodulin-dependent protein kinase IV
C130030J05	3.8582475	P	142.015	36.80811	BC098190	hypothetical protein C130030J05
LOC100046616	3.8550155	P	183.4	47.57436	XM_001476512	similar to aquaporin 5
Vsig10	3.800599	P	75.4033	19.83985	NM_001033311	V-set and immunoglobulin domain containing 10
Thsd7a	3.7911534	P	528.335	139.3599	NM_001164805	thrombospondin, type I, domain containing 7A
Slc5a7	3.7861412	P	493.595	130.3689	NM_022025	solute carrier family 5 (choline transporter), member 7
Ivl	3.7787442	P	173.252	45.84909	NM_008412	involucrin
Prl8a9	3.760617	P	95.3498	25.35483	NM_023332	prolactin family8, subfamily a, member 9
Gm9112	3.75335	P	166.28	44.30174	NM_001177365	predicted gene 9112
Stat1	3.751886	P	40.5144	10.7984	NM_009283	signal transducer and activator of transcription 1
Gm6541	3.712557	P	28.497	7.675841	XM_889508	predicted gene 6541
Arhgap36	3.7008185	P	38.2237	10.32844	NM_001081123	Rho GTPase activating protein 36
Olr1	3.6736085	P	271.305	73.85253	NM_138648	oxidized low density lipoprotein (lectin-like) receptor 1
Bmp6	3.670817	P	126.518	34.46598	NM_007556	bone morphogenetic protein 6
Slc24a3	3.6392903	P	742.845	204.1181	NM_053195	solute carrier family 24 (sodium/potassium/calcium exchanger), member 3
Med4	3.6346517	P	269.47	74.13921	NM_026119	mediator of RNA polymerase II transcription, subunit 4 homolog (yeast)
AU018794	3.6188376	P	55.9995	15.47443	AU018794	expressed sequence AU018794
Asb4	3.612403	P	185.036	51.22244	NM_023048	ankyrin repeat and SOCS box-containing 4
U46068	3.6043239	P	61.8538	17.16101	NM_001012392	cDNA sequence U46068
	3.5895104	P	47.1491	13.13524	AK167780	
Ebf2	3.5808456	P	94.4923	26.38826	NM_010095	early B-cell factor 2
Uchl1	3.57437	P	2668.46	746.5544	NM_011670	ubiquitin carboxy-terminal hydrolase L1
Macrod2	3.5548909	P	77.5843	21.82467	BC076574	MACRO domain containing 2
LOC100041081	3.5505872	P	76.54	21.55699	XM_001475751	similar to RIKEN cDNA 5830484A20 gene
Gm10896	3.5420926	P	245.287	69.24904	XM_001479584	predicted gene 10896
	3.516753	P	160.727	45.70321	XM_001479712	
Car8	3.4967966	P	44.5394	12.73721	NM_007592	carbonic anhydrase 8

Masp2	3.488019	P	54.277	15.56098	NM_010767	mannan-binding lectin serine peptidase 2
	3.466114	P	35.0162	10.10244	AK166446	
	3.4457703	P	94.0928	27.30676	BC062114	
LOC100046827	3.4397113	P	29.9301	8.701343	XM_001476858	similar to CG13990-PA
Ddc	3.4275684	P	543.818	158.6601	NM_016672	dopa decarboxylase
Abca8b	3.4246657	P	57.3737	16.75306	NM_013851	ATP-binding cassette, sub-family A (ABC1), member 8b
Penk	3.4167857	P	473.352	138.5371	NM_001002927	preproenkephalin
Sfrp4	3.4147263	P	54.5495	15.97477	NM_016687	secreted frizzled-related protein 4
	3.4060671	P	32.4525	9.527845	AK047768	
Olfr1251	3.3963997	P	70.2093	20.67168	NM_001011529	olfactory receptor 1251
Hoxa5	3.391915	P	439.403	129.5443	NM_010453	homeobox A5
Olfr770	3.3849025	P	40.8304	12.06251	NM_146863	olfactory receptor 770
Atp10b	3.3813407	P	35.9386	10.62851	NM_176999	ATPase, class V, type 10B
Syt15	3.3679695	P	92.0206	27.32228	NM_181529	synaptotagmin XV
Dtnb	3.3601096	P	185.398	55.17621	NM_007886	dystrobrevin, beta
Cdhr1	3.32831	P	133.659	40.15806	NM_130878	cadherin-related family member 1
1700012L04Rik	3.3172357	P	44.486	13.41055	NM_029588	RIKEN cDNA 1700012L04 gene
Des	3.3166814	P	204.65	61.70323	NM_010043	desmin
Npr3	3.311024	P	619.374	187.0642	NM_008728	natriuretic peptide receptor 3
Ivl	3.2968864	P	98.9412	30.01051	NM_008412	involucrin
Crabp2	3.2857423	P	6555.93	1995.267	NM_007759	cellular retinoic acid binding protein II
Gm15698	3.2799327	P	242.666	73.98509	NR_003564	transcription elongation factor B (SIII), polypeptide 2 pseudogene
Igsf11	3.279912	P	39.6932	12.10191	NM_170599	immunoglobulin superfamily, member 11
Nrcam	3.2583284	P	240.322	73.75613	NM_176930	neuron-glia-CAM-related cell adhesion molecule
LOC634594	3.2463782	P	35.4593	10.92271	XM_909362	similar to Ig heavy chain V region 108A precursor
A2bp1	3.2348082	P	281.268	86.95041	NM_021477	ataxin 2 binding protein 1
Sprr3	3.2280495	P	139.501	43.21532	NM_011478	small proline-rich protein 3
Tnip3	3.2050362	P	173.016	53.98254	NM_001001495	TNFAIP3 interacting protein 3
Npy6r	3.1988401	P	45.075	14.09106	NM_010935	neuropeptide Y receptor Y6

LOC100046676	3.1858974	P	79.4335	24.93284	XM_001476600	hypothetical protein LOC100046676
Cyp2g1	3.182897	P	36.408	11.43864	NM_013809	cytochrome P450, family 2, subfamily g, polypeptide 1
Gm2745	3.1734302	P	69.3642	21.85781	XM_001474423	predicted gene 2745
Lce1f	3.167633	P	4064.41	1283.106	NM_026394	late cornified envelope 1F
	3.1628978	P	22.683	7.171588	AK165756	
Plcb1	3.1580071	P	32.9308	10.4277	U85712	phospholipase C, beta 1
Gm2066	3.1559927	P	67.0599	21.24842	XM_001472346	predicted pseudogene 2066
Zc3h12b	3.150964	P	84.4055	26.78719	NM_001034907	zinc finger CCCH-type containing 12B
Plac8	3.1434383	P	1130.31	359.5779	NM_139198	placenta-specific 8
LOC100046559	3.1410623	P	65.1099	20.72863	XR_031095	similar to 37kDa oncofetal antigen
Rnf182	3.1183274	P	31.2495	10.02123	NM_183204	ring finger protein 182
Gm2496	3.1021147	P	37.0051	11.92899	XM_001473960	predicted gene 2496
Gm52	3.0832913	P	27.0133	8.76118	NM_001013751	predicted gene 52
Whrn	3.0789335	P	853.89	277.3331	NM_001008791	whirlin
Fabp7	3.0754814	P	113.811	37.00584	NM_021272	fatty acid binding protein 7, brain
Tchh	3.07288	P	157.325	51.19803	NM_001163098	trichohyalin
Rpl10l	3.0688875	P	58.4132	19.03398	NM_001162933	ribosomal protein L10-like
Clu	3.0664437	P	426.433	139.0643	NM_013492	clusterin
Serpinb2	3.0655828	P	173.802	56.6946	NM_011111	serine (or cysteine) peptidase inhibitor, clade B, member 2
A330049M08Rik	3.061432	P	56.1935	18.3553	NM_001168500	RIKEN cDNA A330049M08 gene
Nr2f1	3.0532002	P	277.03	90.73415	NM_010151	nuclear receptor subfamily 2, group F, member 1
Rbm24	3.0316336	P	79.5528	26.2409	NM_001081425	RNA binding motif protein 24
	3.0283055	P	63.0453	20.81868		
Tcf4	3.0281544	P	93.3295	30.82057	NM_013685	transcription factor 4
Ptprq	3.023454	P	31.6499	10.46814	NM_001081432	protein tyrosine phosphatase, receptor type, Q
Gm8028	3.0225797	P	30.8791	10.21615	XM_977531	predicted gene 8028
Armcx4	3.008025	P	32.3233	10.74568	BC128507	armadillo repeat containing, X-linked 4
Nrk	3.0069761	P	420.268	139.7643	NM_013724	Nik related kinase
Fam69c	2.992284	P	22.9827	7.680661	NM_173770	family with sequence similarity 69, member C

	2.9807642	P	745.073	249.9603	XM_001000891	
Prl3a1	2.976253	P	1053.47	353.9588	NM_025896	prolactin family 3, subfamily a, member 1
Olfr187	2.9695537	P	142.095	47.85073	NM_146322	olfactory receptor 187
Cdc42bpa	2.9594579	P	201.058	67.93741	NM_001033285	CDC42 binding protein kinase alpha
Klra16	2.9557967	P	630.556	213.3287	NM_013794	killer cell lectin-like receptor, subfamily A, member 16
Srgap3	2.952675	P	498.777	168.9238	NM_080448	SLIT-ROBO Rho GTPase activating protein 3
Cobl	2.9509706	P	2136.38	723.9597	NM_172496	cordon-bleu
Slitrk6	2.9497156	P	29.0017	9.832028	NM_175499	SLIT and NTRK-like family, member 6
Serpini1	2.937361	P	114.746	39.06428	NM_009250	serine (or cysteine) peptidase inhibitor, clade I, member 1
Klra23	2.936769	P	346.514	117.9914	NM_024470	killer cell lectin-like receptor subfamily A, member 23
Parm1	2.9361246	P	3666.29	1248.683	NM_145562	prostate androgen-regulated mucin-like protein 1
B130021B11Rik	2.928057	P	38.1438	13.02699	AK085239	RIKEN cDNA B130021B11 gene
Foxf2	2.920213	P	84.7056	29.00665	NM_010225	forkhead box F2
4930548F15Rik	2.9189825	P	62.5356	21.42378	XM_984432	RIKEN cDNA 4930548F15 gene
Slc2a13	2.9170065	P	900.044	308.5506	NM_001033633	solute carrier family 2 (facilitated glucose transporter), member 13
Gvin1	2.9145298	P	1474.02	505.7488	NM_029000	GTPase, very large interferon inducible 1
9530008L14Rik	2.9118187	P	118.472	40.68646	NM_175417	RIKEN cDNA 9530008L14 gene
Klra23	2.910223	P	489.262	168.1183	NM_024470	killer cell lectin-like receptor subfamily A, member 23
Ooep	2.908148	P	88.4631	30.41905	NM_026480	oocyte expressed protein homolog (dog)
Calr4	2.9013865	P	25.5638	8.810881	NM_001033226	calreticulin 4
Bves	2.8927705	P	693.234	239.6435	NM_024285	blood vessel epicardial substance
LOC100045186	2.8856742	P	42.7828	14.82594	XM_001473816	hypothetical protein LOC100045186
Wdr17	2.8808692	P	49.1264	17.05262	NM_028220	WD repeat domain 17
Slx	2.8717296	P	63.9083	22.2543	NM_001136476	Sycp3 like X-linked
Sprr2a2	2.8710594	P	48.2212	16.79562	NM_001164787	small proline-rich protein 2A2
Megf10	2.8538232	P	1051.78	368.5512	NM_001001979	multiple EGF-like-domains 10
Gm2045	2.8447862	P	1046.16	367.7465	XM_001472254	predicted gene 2045
Klra7	2.8406596	P	970.47	341.6355	NM_001110323	killer cell lectin-like receptor, subfamily A, member 7
	2.8379693	P	27.1657	9.572212	AK015842	

Jam2	2.826669	P	1039.22	367.649	NM_023844	junction adhesion molecule 2
	2.8206716	P	115.12	40.8129	XM_001471969	
Tmem179	2.8193736	P	180.607	64.05916	NM_178915	transmembrane protein 179
Dhh	2.8134227	P	111.381	39.58897	NM_007857	desert hedgehog
Plac8	2.8121378	P	13820.7	4914.646	NM_139198	placenta-specific 8
Pi15	2.8088503	P	39.9749	14.23176	NM_053191	peptidase inhibitor 15
Bche	2.8063047	P	47.8345	17.04536	NM_009738	butyrylcholinesterase
Klra4	2.8055716	P	3680.47	1311.842	NM_010649	killer cell lectin-like receptor, subfamily A, member 4
Akap7	2.794368	P	939.561	336.2338	NM_018747	A kinase (PRKA) anchor protein 7
Fam69c	2.7937174	P	47.0137	16.82838	NM_173770	family with sequence similarity 69, member C
Lce1g	2.7868984	P	1571.98	564.0593	NM_025413	late cornified envelope 1G
4933408N05Rik	2.783679	P	38.4193	13.80164	AK016745	RIKEN cDNA 4933408N05 gene
Scnn1a	2.777286	P	72.5956	26.13905	NM_011324	sodium channel, nonvoltage-gated 1 alpha
Sobp	2.775149	P	68.3708	24.63681	NM_175407	sine oculis-binding protein homolog (Drosophila)
Klra15	2.7688515	P	2540.93	917.6844	NM_013793	killer cell lectin-like receptor, subfamily A, member 15
Gm2012	2.7684996	P	73.9643	26.71639	NM_001104946	predicted gene 2012
Gm4844	2.7622993	P	31.7894	11.50831	XM_140592	predicted gene 4844
Cntf	2.7622106	P	1685.63	610.246	NM_170786	ciliary neurotrophic factor
Gm567	2.7598917	P	250.836	90.88628	XM_923728	predicted gene 567
Nudt10	2.7579029	P	53.6935	19.46894	NM_001031664	nudix (nucleoside diphosphate linked moiety X)-type motif 10
Tgm2	2.7554996	P	17374.3	6305.322	NM_009373	transglutaminase 2, C polypeptide
Cd200	2.7458236	P	35.7503	13.01988	NM_010818	CD200 antigen
Tmem179	2.7458217	P	141.8	51.64203	NM_178915	transmembrane protein 179
Gm9298	2.744165	P	44.9816	16.39172	XR_030681	predicted gene 9298
Hmox1	2.7439084	P	11142.7	4060.898	NM_010442	heme oxygenase (decycling) 1
Akr1c19	2.7432654	P	151.667	55.28706	NM_001013785	aldo-keto reductase family 1, member C19
Mtap7d2	2.7428036	P	33.8366	12.3365	NM_001081124	MAP7 domain containing 2
Dmd	2.7315705	P	87.0793	31.87885	NM_007868	dystrophin, muscular dystrophy
Gm13105	2.7294164	P	214.795	78.69635	XM_001475876	predicted gene 13105

	2.7093904	P	29.0863	10.73537	AK036542	
Sh3gl3	2.702541	P	295.952	109.5086	NM_017400	SH3-domain GRB2-like 3
	2.6965032	P	571.297	211.866	AK149398	
Dner	2.6945765	P	741.25	275.0895	NM_152915	delta/notch-like EGF-related receptor
Cobl	2.6918533	P	171.558	63.73241	NM_172496	cordon-bleu
4930521A18Rik	2.6875143	P	100.999	37.58078	NM_029062	RIKEN cDNA 4930521A18 gene
Tbx2	2.6871264	P	91.6716	34.11509	NM_009324	T-box 2
Nr2f1	2.6825554	P	762.166	284.1193	NM_010151	nuclear receptor subfamily 2, group F, member 1
Fhit	2.6666262	P	65.2374	24.4644	NM_010210	fragile histidine triad gene
Gata6	2.6633525	P	301.361	113.1511	NM_010258	GATA binding protein 6
Lce1l	2.6627781	P	135.277	50.80292	NM_028628	late cornified envelope 1L
Gm5935	2.6524677	P	93.2576	35.15882	NM_001081657	predicted gene 5935
Gata2	2.6494823	P	236.069	89.1002	NM_008090	GATA binding protein 2
Fads6	2.6483479	P	55.196	20.84167	NM_178035	fatty acid desaturase domain family, member 6
Prl2c1	2.63673	P	1714.53	650.2474	NM_001045532	Prolactin family 2, subfamily c, member 1
Ubd	2.6342812	P	90.2212	34.24888	NM_023137	ubiquitin D
Tinagl1	2.6330984	P	4169.01	1583.308	NM_023476	tubulointerstitial nephritis antigen-like 1
	2.6330607	P	101.897	38.69915	AK077802	
Nbeal1	2.6324718	P	34.9594	13.28006	NM_173444	neurobeachin like 1
Klra15	2.6317232	P	358.483	136.216	NM_013793	killer cell lectin-like receptor, subfamily A, member 15
Alcam	2.6315794	P	1135.97	431.6693	NM_009655	activated leukocyte cell adhesion molecule
Klra33	2.630162	P	403.092	153.2573	NM_001039118	killer cell lectin-like receptor subfamily A member 33
4930486L24Rik	2.6236374	P	1126.59	429.3981	NM_178098	RIKEN cDNA 4930486L24 gene
Prss12	2.623437	P	457.3	174.3133	NM_008939	protease, serine, 12 neurotrypsin (motopsin)
Gria4	2.6222665	P	45.4624	17.33705	NM_001113180	glutamate receptor, ionotropic, AMPA4 (alpha 4)
Nup62cl	2.6111271	P	129.862	49.73393	NM_001081668	nucleoporin 62 C-terminal like
A230103O09Rik	2.604944	P	41.8739	16.07477	XM_001471948	RIKEN cDNA A230103O09 gene
Rasl10a	2.5982342	P	50.4566	19.41956	NM_145216	RAS-like, family 10, member A
Crct1	2.5969818	P	1478.16	569.183	NM_028798	cysteine-rich C-terminal 1

Galnt14	2.5968199	P	50.113	19.29785	NM_027864	UDP-N-acetyl-alpha-D-galactosamine:polypeptide N-acetylgalactosaminyltransferase 14
Olfr2	2.593103	P	29.8183	11.49907	NM_010983	olfactory receptor 2
Ccdc81	2.5922277	P	611.104	235.7446	NM_001162979	coiled-coil domain containing 81
Pmfbp1	2.586779	P	39.5499	15.28926	NM_019938	polyamine modulated factor 1 binding protein 1
Calml4	2.5803196	P	35.7633	13.86001	NM_138304	calmodulin-like 4
	2.5771322	P	30.9532	12.01071	AK080825	
Gm11202	2.568462	P	47.8083	18.6136	NR_003283	predicted gene 11202
LOC677487	2.5624607	P	70.6359	27.56564	XM_001004010	similar to ubiquitin A-52 residue ribosomal protein fusion product 1
BC032203	2.5604334	P	32.8454	12.82807	NM_001100452	cDNA sequence BC032203
Nr0b2	2.558115	P	23.5135	9.191721	NM_011850	nuclear receptor subfamily 0, group B, member 2
Ces5	2.5561016	P	90.3779	35.3577	NM_172759	carboxylesterase 5
Gm3064	2.554421	P	329.664	129.0563	XM_001475563	predicted gene 3064
Tinagl1	2.5517452	P	1416.74	555.2043	NM_023476	tubulointerstitial nephritis antigen-like 1
Rarb	2.5505662	P	24.0537	9.430711	NM_011243	retinoic acid receptor, beta
AW493563	2.5472732	P	58.3347	22.90084	DV072245	expressed sequence AW493563
Kcnk15	2.5419023	P	204.99	80.64433	NM_001030292	potassium channel, subfamily K, member 15
Prss46	2.5391822	P	302.805	119.2529	NM_183103	protease, serine, 46
Zfp13	2.5351002	P	295.165	116.4313	NM_011747	zinc finger protein 13
Gsg1	2.5321748	P	286.405	113.1063	NM_010352	germ cell-specific gene 1
Msln	2.5280101	P	63.03	24.93265	NM_018857	mesothelin
Glipr1	2.527772	P	3184.15	1259.666	NM_028608	GLI pathogenesis-related 1 (glioma)
A730032A03Rik	2.526839	P	30.7575	12.17231	AK029486	RIKEN cDNA A730032A03 gene
LOC100045389	2.5191162	P	80.7945	32.07255	XR_031631	similar to mitochondrial ribosomal protein S16
Rln1	2.515052	P	49.4334	19.65501	NM_011272	relaxin 1
Fgr	2.5078838	P	32.2017	12.8402	NM_010208	Gardner-Rasheed feline sarcoma viral (Fgr) oncogene homolog
Gm5144	2.5045557	P	103.987	41.51894	XR_035213	predicted gene 5144
Wnt2	2.5018556	P	29.6681	11.85844	NM_023653	wingless-related MMTV integration site 2
Clstn2	2.500657	P	72.8717	29.14101	NM_022319	calsyntenin 2
Sema3c	2.4972432	P	3218.05	1288.642	NM_013657	sema, immunoglobulin domain (Ig), short basic domain, secreted, (semaphorin) 3C

Scx	2.4968135	P	764.987	306.3853	NM_198885	scleraxis
Cadm2	2.4961379	P	62.8254	25.16906	NM_178721	cell adhesion molecule 2
Fgf18	2.491404	P	164.156	65.88883	NM_008005	fibroblast growth factor 18
Dync1i1	2.4899611	P	28.399	11.40542	NM_010063	dynein cytoplasmic 1 intermediate chain 1
Fam84a	2.4859405	P	24.7974	9.975053	NM_029007	family with sequence similarity 84, member A
Ogn	2.483417	P	3358.45	1352.351	NM_008760	osteoglycin
Ager	2.4809089	P	21.6198	8.714447	NM_007425	advanced glycosylation end product-specific receptor
Neurog2	2.4798682	P	23.3813	9.428437	NM_009718	neurogenin 2
Vmn1r53	2.4754972	P	22.6288	9.141128	NM_053226	vomeronasal 1 receptor 53
B230117O15Rik	2.4729128	P	196.099	79.29868	AK020978	RIKEN cDNA B230117O15 gene
Gbe1	2.4717846	P	1289.71	521.7708	NM_028803	glucan (1,4-alpha-), branching enzyme 1
Chd7	2.4700496	P	647.747	262.2406	NM_001081417	chromodomain helicase DNA binding protein 7
Zfp536	2.4635327	P	34.2882	13.91831	NM_172385	zinc finger protein 536
Shank3	2.460151	P	25.7829	10.48019	NM_021423	SH3/ankyrin domain gene 3
Mst1	2.4591265	P	35.2888	14.35014	NM_008243	macrophage stimulating 1 (hepatocyte growth factor-like)
Ccbe1	2.4582741	P	138.339	56.27472	NM_178793	collagen and calcium binding EGF domains 1
Cdh13	2.4511483	P	802.167	327.2617	NM_019707	cadherin 13
1700109H08Rik	2.4499924	P	37.2812	15.21686	NM_029843	RIKEN cDNA 1700109H08 gene
Aox1	2.4469209	P	325.486	133.0187	NM_009676	aldehyde oxidase 1
Gpr82	2.4412506	P	36.579	14.98371	NM_175669	G protein-coupled receptor 82
Klra16	2.4388032	P	1597.75	655.1353	NM_013794	killer cell lectin-like receptor, subfamily A, member 16
Mt2	2.4371006	P	14226.1	5837.289	NM_008630	metallothionein 2
A2bp1	2.435011	P	140.335	57.63215	NM_021477	ataxin 2 binding protein 1
D930032P07Rik	2.4327765	P	24.5883	10.1071	XM_001477595	RIKEN cDNA D930032P07 gene
Adcy5	2.4293997	P	92.132	37.92378	NM_001012765	adenylate cyclase 5
Dnahc7b	2.4279077	P	21.9348	9.03445	NM_001160386	dynein, axonemal, heavy chain 7B
E230025N22Rik	2.4244893	P	34.4109	14.19304	NM_172831	Riken cDNA E230025N22 gene
Plcb1	2.424403	P	72.6193	29.95347	NM_019677	phospholipase C, beta 1
Unc93a	2.4202948	P	25.2811	10.44548	NM_199252	unc-93 homolog A (C. elegans)

Hoxa3	2.4189987	P	748.712	309.5133	NM_010452	homeobox A3
Bace2	2.4169145	P	547.888	226.6892	NM_019517	beta-site APP-cleaving enzyme 2
Plau	2.4121516	P	2339.53	969.8934	NM_008873	plasminogen activator, urokinase
Klra12	2.4081643	P	1122.76	466.2307	NM_010646	killer cell lectin-like receptor subfamily A, member 12
Klra22	2.4067068	P	1209.46	502.5369	NM_053152	killer cell lectin-like receptor subfamily A, member 22
F11r	2.401762	P	1072.47	446.5351	NM_172647	F11 receptor
Sytl1	2.399409	P	61.8429	25.7742	NM_031393	synaptotagmin-like 1
	2.3985624	P	58.6371	24.44676	XR_035617	
Gm9112	2.3967881	P	85.809	35.80167	NM_001177365	predicted gene 9112
Fam19a5	2.3963103	P	112.879	47.10547	NM_134096	family with sequence similarity 19, member A5
Lama1	2.391425	P	69.9936	29.26857	NM_008480	laminin, alpha 1
Tgm2	2.3858197	P	6677.23	2798.714	NM_009373	transglutaminase 2, C polypeptide
Mtus2	2.3756359	P	702.938	295.8948	NM_029920	microtubule associated tumor suppressor candidate 2
Olfr1519	2.3735304	P	21.716	9.149222	NM_146399	olfactory receptor 1519
F8	2.3728006	P	51.3794	21.65348	NM_007977	coagulation factor VIII
Lhx9	2.3723528	P	214.387	90.36876	NM_001025565	LIM homeobox protein 9
Tpd52l1	2.3721316	P	1130.66	476.6418	NM_009413	tumor protein D52-like 1
Unc13b	2.3718991	P	185.478	78.19793	NM_001081413	unc-13 homolog B (C. elegans)
Slc6a15	2.368014	P	32.5641	13.75167	NM_175328	solute carrier family 6 (neurotransmitter transporter), member 15
Maoa	2.3665888	P	1741.36	735.8118	NM_173740	monoamine oxidase A
Dgkk	2.3647296	P	914.187	386.5926	NM_177914	diacylglycerol kinase kappa
Cmbl	2.3621817	P	103.531	43.82847	NM_181588	carboxymethylenebutenolidase-like (Pseudomonas)
Fggy	2.3616755	P	116.651	49.39336	NM_029347	FGGY carbohydrate kinase domain containing
Gvin1	2.3603995	P	1193.36	505.5771	NM_029000	GTPase, very large interferon inducible 1
Wnt10b	2.3589401	P	79.1161	33.53882	NM_011718	wingless related MMTV integration site 10b
Unc5c	2.3589354	P	98.4742	41.74517	NM_009472	unc-5 homolog C (C. elegans)
Prl3d1	2.3570273	P	444.466	188.5704	NM_008864	prolactin family 3, subfamily d, member 1
Sytl4	2.3537216	P	88.4882	37.59503	NM_013757	synaptotagmin-like 4
LOC100047504	2.3510125	P	47.15	20.05519	XM_001478297	similar to MTH2 protein

4930513E20Rik	2.3503666	P	109.6	46.63081	AK019679	RIKEN cDNA 4930513E20 gene
Aqp5	2.3480735	P	131.63	56.05877	NM_009701	aquaporin 5
Fgf5	2.3442903	P	67.9497	28.98519	NM_010203	fibroblast growth factor 5
LOC630737	2.3432834	P	80.9452	34.54348	XR_031251	similar to Histone H3.4 (Embryonic)
Tnni3k	2.3429785	P	313.696	133.8876	NM_177066	TNNI3 interacting kinase
Pax3	2.3400216	P	38.8964	16.62222	NM_008781	paired box gene 3
LOC100048018	2.3394008	P	41.0696	17.55559	XM_001479650	similar to complement component factor H
Rcan2	2.3387923	P	875.18	374.2018	NM_207649	regulator of calcineurin 2
Cfh	2.3384216	P	845.417	361.5333	NM_009888	complement component factor h
Xkr6	2.3379576	P	22.7213	9.71843	NM_173393	X Kell blood group precursor related family member 6 homolog
Trim6	2.3283782	P	28.2638	12.13885	NM_001013616	tripartite motif-containing 6
Pdx1	2.3280847	P	34.9877	15.02855	NM_008814	pancreatic and duodenal homeobox 1
	2.327742	P	45.3239	19.47118	AK030497	
Gm1966	2.3248181	P	133.805	57.55521	XM_001000891	predicted gene 1966
Klra22	2.3240333	P	23.1434	9.958295	NM_053152	killer cell lectin-like receptor subfamily A, member 22
Mme	2.322362	P	27.8159	11.97744	NM_008604	membrane metallo endopeptidase
Gm3925	2.3127227	P	49.3395	21.33395	XM_001478596	predicted gene 3925
Col8a2	2.30811	P	577.47	250.1916	NM_199473	collagen, type VIII, alpha 2
Gm13646	2.3080354	P	129.717	56.20234	NM_001097979	predicted gene 13646
Cpne8	2.3010256	P	688.621	299.2668	NM_001033851	copine VIII
Svop	2.300861	P	35.7781	15.54986	NM_026805	SV2 related protein
Zfp697	2.300509	P	597.435	259.697	NM_172863	zinc finger protein 697
Spnb4	2.2992187	P	199.06	86.57737	NM_032610	spectrin beta 4
Cxcl12	2.2991467	P	7331.69	3188.873	NM_021704	chemokine (C-X-C motif) ligand 12
Ctla2a	2.2971866	P	39975.2	17401.81	NM_007796	cytotoxic T lymphocyte-associated protein 2 alpha
Zdhhc17	2.2957184	P	206.254	89.84281	NM_172554	zinc finger, DHHC domain containing 17
Smad6	2.294661	P	669.466	291.7493	NM_008542	MAD homolog 6 (Drosophila)
Syn2	2.2943187	P	45.4558	19.81232	NM_013681	synapsin II
Slco1a5	2.2938812	P	60.5439	26.39366	NM_130861	solute carrier organic anion transporter family, member 1a5

Sim2	2.2936876	P	66.6383	29.0529	NM_011377	single-minded homolog 2 (Drosophila)
Tceal5	2.2900481	P	328.144	143.2914	NM_177919	transcription elongation factor A (SII)-like 5
9930013L23Rik	2.288655	P	63.7033	27.83437	NM_030728	RIKEN cDNA 9930013L23 gene
Hist2h3c2-ps	2.2863185	P	1596.39	698.2365	NM_054045	histone cluster 2, H3c2, pseudogene
	2.2822948	P	56.7609	24.87009	AK004111	
Nipal3	2.278201	P	263.333	115.5881	NM_028995	NIPA-like domain containing 3
Ppm1h	2.2779684	P	409.406	179.7243	NM_001110218	protein phosphatase 1H (PP2C domain containing)
	2.2779121	P	233.812	102.6432	XR_035461	
Klra16	2.2770813	P	338.937	148.8473	NM_013794	killer cell lectin-like receptor, subfamily A, member 16
Olfr1344	2.2759633	P	20.4895	9.002575	NM_177061	olfactory receptor 1344
Papln	2.274558	P	29.4305	12.93901	NM_130887	papilin, proteoglycan-like sulfated glycoprotein
Sema3c	2.265675	P	419.484	185.1474	NM_013657	sema, immunoglobulin domain (Ig), short basic domain, secreted, (semaphorin) 3C
Bach2	2.2647934	P	20.05	8.852904	NM_001109661	BTB and CNC homology 2
Gjb3	2.2631958	P	1040.44	459.7211	NM_001160012	gap junction protein, beta 3
Cntf	2.2620134	P	378.579	167.3637	NM_170786	ciliary neurotrophic factor
Htr2b	2.2617545	P	79.5069	35.15274	NM_008311	5-hydroxytryptamine (serotonin) receptor 2B
Gdnf	2.2581484	P	72.5552	32.1304	NM_010275	glial cell line derived neurotrophic factor
Txlnb	2.2580898	P	40.945	18.1326	NM_138628	taxilin beta
Pax7	2.2572842	P	25.3216	11.21774	NM_011039	paired box gene 7
5033411D12Rik	2.256775	P	59.3661	26.30573	NM_138654	RIKEN cDNA 5033411D12 gene
Mdga1	2.256472	P	346.431	153.5278	NM_001081160	MAM domain containing glycosylphosphatidylinositol anchor 1
	2.2535803	P	29.2195	12.96582	AK028130	
Pappa	2.2534225	P	4142.19	1838.177	NM_021362	pregnancy-associated plasma protein A
LOC635513	2.2528236	P	160.277	71.14489	XM_910588	Ly49p/d
Dnahc10	2.2500112	P	48.119	21.3861	NM_019536	dynein, axonemal, heavy chain 10
Anxa8	2.2484043	P	8428.25	3748.547	NM_013473	annexin A8
Tnfsf18	2.2470858	P	851.647	379.0008	NM_183391	tumor necrosis factor (ligand) superfamily, member 18
Lhx9	2.2420774	P	197.078	87.89986	NM_001042577	LIM homeobox protein 9
2310043J07Rik	2.2418802	P	82.9628	37.00588	NM_027158	RIKEN cDNA 2310043J07 gene

Ttyh2	2.2412305	P	175.579	78.34044	NM_053273	tweety homolog 2 (Drosophila)
Mmp1b	2.2402716	P	17.114	7.639235	NM_032007	matrix metallopeptidase 1b (interstitial collagenase)
	2.2326868	P	21.236	9.511393	AY672066	
2610528A11Rik	2.2317824	P	173.242	77.625	XM_980662	RIKEN cDNA 2610528A11 gene
Glyctk	2.2290528	P	56.7346	25.45234	NM_174846	glycerate kinase
9530078M16Rik	2.227189	P	31.732	14.24756	AK035630	RIKEN cDNA 9530078M16 gene
Gm5168	2.2271829	P	44.0541	19.78017	NM_001025607	predicted gene 5168
Gm6664	2.2237914	P	49.7725	22.38182	XR_035618	predicted gene 6664
Foxc1	2.2223544	P	580.87	261.3761	NM_008592	forkhead box C1
Timp2	2.221992	P	136408	61390.09	NM_011594	tissue inhibitor of metalloproteinase 2
Bean	2.2217302	P	84.7667	38.15346	NM_001141922	brain expressed, associated with Nedd4
Cd24a	2.220066	P	3545.6	1597.071	NM_009846	CD24a antigen
Stbd1	2.2185476	P	6329.88	2853.163	NM_175096	starch binding domain 1
Cdkn1c	2.2178779	P	1746.05	787.2611	NM_009876	cyclin-dependent kinase inhibitor 1C (P57)
Fabp3	2.217571	P	3219.26	1451.707	NM_010174	fatty acid binding protein 3, muscle and heart
Hist1h2be	2.2174203	P	465.353	209.8622	NM_178194	histone cluster 1, H2be
Mst1	2.2153447	P	28.7604	12.98235	NM_008243	macrophage stimulating 1 (hepatocyte growth factor-like)
Gsta3	2.2137156	P	116.85	52.78474	NM_001077353	glutathione S-transferase, alpha 3
9930013L23Rik	2.2104557	P	70.7301	31.99797	NM_030728	RIKEN cDNA 9930013L23 gene
Nox4	2.2097173	P	781.301	353.575	NM_015760	NADPH oxidase 4
Plagl1	2.209045	P	1753.6	793.8268	NM_009538	pleiomorphic adenoma gene-like 1
D5Ertd605e	2.208279	P	247.282	111.9796	NR_033625	DNA segment, Chr 5, ERATO Doi 605, expressed
Rasgrp3	2.208128	P	802.963	363.6399	NM_207246	RAS, guanyl releasing protein 3
Macc1	2.2060437	P	327.255	148.3449	NM_001163136	metastasis associated in colon cancer 1
Bst1	2.2042854	P	38.1159	17.29172	NM_009763	bone marrow stromal cell antigen 1
Nms	2.202608	P	17.8869	8.120772	NM_001011684	neuromedin S
Unc5c	2.20236	P	43.3077	19.66424	NM_009472	unc-5 homolog C (C. elegans)
C430002N11Rik	2.2022018	P	155.421	70.57537	AK049386	RIKEN cDNA C430002N11 gene
Ppp1r3a	2.1987486	P	40.4179	18.38221	NM_080464	protein phosphatase 1, regulatory (inhibitor) subunit 3A

Dync1i1	2.1959715	P	67.4759	30.72713	NM_010063	dynein cytoplasmic 1 intermediate chain 1
Prrt4	2.19131	P	80.1115	36.55874	NM_001101443	proline-rich transmembrane protein 4
Pla2g12b	2.1901245	P	85.3644	38.97694	NM_023530	phospholipase A2, group XIIB
Plagl1	2.1894655	P	1708	780.0972	NM_009538	pleiomorphic adenoma gene-like 1
Sqrdl	2.1874185	P	3369.03	1540.183	NM_021507	sulfide quinone reductase-like (yeast)
Pramef12	2.185524	P	16.9403	7.751147	NM_029948	PRAME family member 12
Hist1h2bg	2.1845336	P	243.021	111.2464	NM_178196	histone cluster 1, H2bg
Ctnnd2	2.1834717	P	295.814	135.4788	NM_008729	catenin (cadherin associated protein), delta 2
Gm9804	2.182495	P	41.8935	19.19524	XM_914998	predicted gene 9804
LOC100046778	2.1814756	P	92.5402	42.42093	XM_001476791	hypothetical protein LOC100046778
1810062O18Rik	2.178545	P	215.495	98.91698	NR_033571	RIKEN cDNA 1810062O18 gene
Anxa8	2.177512	P	1330.04	610.8082	NM_013473	annexin A8
Cd59a	2.1769233	P	943.767	433.5325	NM_007652	CD59a antigen
E130118H10Rik	2.1728468	P	17.745	8.166715	AK165039	RIKEN cDNA E130118H10 gene
Mocos	2.1727407	P	937.642	431.5478	NM_026779	molybdenum cofactor sulfurase
Xk	2.1726813	P	209.255	96.31173	NM_023500	Kell blood group precursor (McLeod phenotype) homolog
Gata3	2.1682265	P	381.422	175.9141	NM_008091	GATA binding protein 3
2610019F03Rik	2.168222	P	142.98	65.94361	NM_173744	RIKEN cDNA 2610019F03 gene
4933439N14Rik	2.1670713	P	20.8101	9.602877	AK019859	RIKEN cDNA 4933439N14 gene
Asb5	2.1659622	P	30.9681	14.29762	NM_029569	ankyrin repeat and SOCs box-containing 5
BC026782	2.1616035	P	187.566	86.77146	NM_001025575	cDNA sequence BC026782
Tmem37	2.1599064	P	9154.73	4238.487	NM_019432	transmembrane protein 37
Scrn1	2.159689	P	935.411	433.123	AK129084	secernin 1
Nhedc2	2.1583815	P	44.8841	20.79528	NM_178877	Na+/H+ exchanger domain containing 2
Hist2h2aa1	2.1581376	P	9444.33	4376.148	NM_013549	histone cluster 2, H2aa1
Xlr	2.156138	P	51.2245	23.75751	NM_011725	X-linked lymphocyte-regulated complex
Nqo1	2.1487932	P	975.384	453.9219	NM_008706	NAD(P)H dehydrogenase, quinone 1
2310057B04Rik	2.147614	P	36.9611	17.21033	XM_001476219	RIKEN cDNA 2310057B04 gene
Pnma2	2.1446173	P	81.1553	37.84137	NM_175498	paraneoplastic antigen MA2

Sparcl1	2.142515	P	171.23	79.91986	NM_010097	SPARC-like 1
Sec14l2	2.1417763	P	72.4874	33.84451	NM_144520	SEC14-like 2 (S. cerevisiae)
LOC676755	2.138043	P	155.057	72.52306	XM_992608	similar to U2-associated SR140 protein
Adamts17	2.1323252	P	195.107	91.49965	NM_001033877	a disintegrin-like and metallopeptidase with thrombospondin type 1 motif, 17
Nup62cl	2.1316993	P	130.4	61.17167	NM_001081668	nucleoporin 62 C-terminal like
Npas3	2.1297538	P	114.431	53.72973	NM_013780	neuronal PAS domain protein 3
Fam46c	2.1295962	P	126.496	59.39909	NM_001142952	family with sequence similarity 46, member C
Suv420h1	2.1294634	P	290.147	136.2534	NM_001167885	suppressor of variegation 4-20 homolog 1 (Drosophila)
Fabp3	2.1290114	P	3836.11	1801.825	NM_010174	fatty acid binding protein 3, muscle and heart
Ctnnal1	2.1281152	P	696.955	327.4985	NM_018761	catenin (cadherin associated protein), alpha-like 1
LOC676300	2.125966	P	86.3102	40.59813	XM_988868	similar to leucine-rich repeat-containing G protein-coupled receptor 6 isoform 1
Ankrd44	2.1246214	P	870.282	409.6175	NM_001081433	ankyrin repeat domain 44
Cd101	2.1236336	P	99.2283	46.72571	NM_001167906	CD101 antigen
Adamtsl4	2.1234062	P	665.661	313.4873	NM_144899	ADAMTS-like 4
Fggy	2.1180868	P	303.349	143.2184	NM_001113412	FGGY carbohydrate kinase domain containing
Dach2	2.1171064	P	235.178	111.0847	NM_033605	dachshund 2 (Drosophila)
Oosp1	2.114223	P	46.7601	22.11693	NM_133353	oocyte secreted protein 1
Gpr56	2.1131368	P	43.9538	20.80024	NM_018882	G protein-coupled receptor 56
Chst13	2.1115582	P	22.3375	10.57867	NM_027928	carbohydrate (chondroitin 4) sulfotransferase 13
Ephx2	2.1111703	P	1129.97	535.2315	NM_007940	epoxide hydrolase 2, cytoplasmic
Ctnnd2	2.11094	P	332.987	157.7435	NM_008729	catenin (cadherin associated protein), delta 2
Chrnb4	2.1108973	P	181.391	85.93079	NM_148944	cholinergic receptor, nicotinic, beta polypeptide 4
Upk2	2.1086485	P	18.8119	8.921287	NM_009476	uroplakin 2
Fgf12	2.1077838	P	34.9246	16.56935	NM_183064	fibroblast growth factor 12
H2-Ab1	2.1070924	P	51.0366	24.22134	NM_207105	histocompatibility 2, class II antigen A, beta 1
Tcfec	2.107041	P	94.0982	44.65893	NM_031198	transcription factor EC
Lhx6	2.1046124	P	55.6742	26.45344	NM_008500	LIM homeobox protein 6
Col4a6	2.1032374	P	369.646	175.751	NM_053185	collagen, type IV, alpha 6
Bex1	2.101776	P	101.154	48.12787	NM_009052	brain expressed gene 1

Cnih2	2.0987113	P	583.226	277.897	NM_009920	cornichon homolog 2 (Drosophila)
Dhrs3	2.098507	P	6113.58	2913.298	NM_011303	dehydrogenase/reductase (SDR family) member 3
	2.097444	P	34.9879	16.68119	XM_001478361	
	2.096222	P	91.9462	43.86283	XM_001477292	
Ptchd1	2.0958238	P	75.7798	36.15752	NM_001093750	patched domain containing 1
2310043J07Rik	2.095571	P	73.8246	35.22886	NM_027158	RIKEN cDNA 2310043J07 gene
AA415398	2.0954194	P	284.693	135.8645	NM_001004178	expressed sequence AA415398
Eaf2	2.0943675	P	97.6374	46.61902	NM_001113401	ELL associated factor 2
Adm	2.0941665	P	3898.08	1861.399	NM_009627	adrenomedullin
Olfr449	2.093949	P	20.0457	9.573151	NM_147064	olfactory receptor 449
Fgf18	2.092834	P	411.429	196.5895	NM_008005	fibroblast growth factor 18
Nudt11	2.0926838	P	70.5264	33.7014	NM_021431	nudix (nucleoside diphosphate linked moiety X)-type motif 11
4933427G17Rik	2.091613	P	50.7806	24.27817	XM_133717	RIKEN cDNA 4933427G17 gene
Prickle2	2.0908453	P	72.8708	34.85231	NM_001134460	prickle homolog 2 (Drosophila)
C030027H14Rik	2.0902214	P	1565.69	749.056	AK021106	RIKEN cDNA C030027H14 gene
Moxd1	2.087808	P	725.59	347.5367	NM_021509	monooxygenase, DBH-like 1
Gm4005	2.086878	P	25.2493	12.09906	XM_001476146	predicted gene 4005
Mamdc2	2.0868108	P	701.387	336.1046	NM_174857	MAM domain containing 2
Gimap6	2.0862634	P	66.7499	31.99497	NM_153175	GTPase, IMAP family member 6
H2-M1	2.0853372	P	32.9956	15.82266	NM_177636	histocompatibility 2, M region locus 1
9530077C14Rik	2.0850015	P	197.099	94.53202	XM_001479413	RIKEN cDNA 9530077C14 gene
Me3	2.0821028	P	332.044	159.4751	NM_181407	malic enzyme 3, NADP(+)-dependent, mitochondrial
Dpyd	2.0813239	P	26.5168	12.74034	NM_170778	dihydropyrimidine dehydrogenase
Rnf207	2.0797632	P	78.0832	37.54427	NM_001033489	ring finger protein 207
Ndrg2	2.076809	P	513.251	247.1346	NM_013864	N-myc downstream regulated gene 2
Mtf1	2.0753126	P	98.1333	47.28601	NM_008636	metal response element binding transcription factor 1
Ddc8	2.074995	P	736.038	354.7177	NM_021440	differential display clone 8
Pclo	2.0726252	P	47.6408	22.98575	NM_001110796	piccolo (presynaptic cytomatrix protein)
Fam59b	2.0699723	P	831.073	401.4901	NM_001167879	family with sequence similarity 59, member B

Tgtp2	2.0675669	P	238.068	115.1438	NM_001145164	T-cell specific GTPase 2
	2.0666728	P	79.2504	38.34687	AK172359	
C230060E24	2.0665236	P	2242.28	1085.047	AK048775	hypothetical protein C230060E24
AU021092	2.0658622	P	24.7176	11.96481	NM_001033220	expressed sequence AU021092
Serpinb1a	2.0650976	P	359.53	174.0981	NM_025429	serine (or cysteine) peptidase inhibitor, clade B, member 1a
Gm7921	2.0639765	P	20.159	9.767074	XM_981456	predicted gene 7921
	2.0615673	P	26.1438	12.68152	XM_001472477	
Hoxc13	2.0590887	P	45.2177	21.96005	NM_010464	homeobox C13
AI646023	2.0589564	P	39.6459	19.25534	NM_198860	expressed sequence AI646023
2610019F03Rik	2.058662	P	792.053	384.7416	NM_173744	RIKEN cDNA 2610019F03 gene
Myo1e	2.055806	P	2467.81	1200.409	NM_181072	myosin IE
LOC552901	2.0550208	P	47.175	22.95598	AK035826	hypothetical LOC552901
Pdzk1	2.0522785	P	33.0383	16.09835	NM_021517	PDZ domain containing 1
Mkx	2.0502908	P	119.034	58.05694	NM_177595	mohawk homeobox
LOC634585	2.0501204	P	747.671	364.6963	XM_909351	similar to Killer cell lectin-like receptor 4 (Ly49-D antigen) (Lymphocyte antigen 49D)
Grip1	2.0499718	P	66.5126	32.44563	NM_028736	glutamate receptor interacting protein 1
	2.049539	P	610.341	297.7944		
Cdkn2b	2.0494118	P	16116.7	7864.037	NM_007670	cyclin-dependent kinase inhibitor 2B (p15, inhibits CDK4)
Gsta4	2.0452619	P	313.094	153.0825	NM_010357	glutathione S-transferase, alpha 4
6330407I18Rik	2.0443935	P	1812.06	886.3568	AK018137	RIKEN cDNA 6330407I18 gene
Fxyd3	2.0425687	P	34.8954	17.0841	NM_008557	FXYD domain-containing ion transport regulator 3
Olfr90	2.042006	P	21.6301	10.59257	NM_146477	olfactory receptor 90
Prl	2.0401793	P	296.246	145.206	NM_011164	prolactin
EG665756	2.0397298	P	31.0035	15.19979	XM_979235	predicted gene, EG665756
Hist1h2bc	2.0377731	P	53736.2	26370.08	NM_023422	histone cluster 1, H2bc
Cdkn2a	2.0368927	P	39408.1	19347.15	NM_009877	cyclin-dependent kinase inhibitor 2A
Hspb7	2.033879	P	33.4289	16.43602	NM_013868	heat shock protein family, member 7 (cardiovascular)
	2.0335848	P	219.457	107.9165	XM_001479396	
Afap1l2	2.0317702	P	177.894	87.55602	NM_146102	actin filament associated protein 1-like 2

Car13	2.0284011	P	265.541	130.9115	NM_024495	carbonic anhydrase 13
Nkain3	2.0240996	P	127.133	62.80975	NM_172987	Na+/K+ transporting ATPase interacting 3
Sprr1a	2.0224717	P	565.211	279.4657	NM_009264	small proline-rich protein 1A
Kcna7	2.0215356	P	202.323	100.0838	NM_010596	potassium voltage-gated channel, shaker-related subfamily, member 7
Ttll11	2.0202801	P	1522.17	753.445	NM_029774	tubulin tyrosine ligase-like family, member 11
Als2cl	2.0189936	P	289.529	143.4028	NM_001146060	ALS2 C-terminal like
Cmya5	2.0188036	P	19.0979	9.460029	NM_023821	cardiomyopathy associated 5
Nat8b	2.0179389	P	45.6573	22.62573	XM_485799	N-acetyltransferase 8B
1700020G03Rik	2.0174348	P	24.315	12.05245	AK006158	RIKEN cDNA 1700020G03 gene
Fbxl21	2.0162644	P	27.9797	13.87702	NM_178674	F-box and leucine-rich repeat protein 21
Slc16a4	2.0146015	P	255.295	126.7222	NM_146136	solute carrier family 16 (monocarboxylic acid transporters), member 4
A630026N12Rik	2.014018	P	84.306	41.85958	AK035805	RIKEN cDNA A630026N12 gene
Frmd3	2.0133173	P	235.978	117.2085	NM_172869	FERM domain containing 3
Chgb	2.0127823	P	51.8136	25.74226	NM_007694	chromogranin B
Adcy5	2.0097487	P	35.0105	17.42033	NM_001012765	adenylate cyclase 5
	2.0085483	P	44.3039	22.05768	XM_001471461	
LOC100047092	2.0085423	P	20.9731	10.44197	XM_001477369	similar to Heat shock protein 1 (chaperonin)
Kcnj4	2.0080335	P	274.005	136.4543	NM_008427	potassium inwardly-rectifying channel, subfamily J, member 4
Neto1	2.0070145	P	17.7006	8.819359	NM_144946	neuropilin (NRP) and tolloid (TLL)-like 1
Gm13140	2.0041285	P	27.5528	13.748	XR_032815	predicted gene 13140
Ppm1h	2.0008023	P	300.691	150.2854	NM_001110218	protein phosphatase 1H (PP2C domain containing)
Itgbl1	2.00075	P	91.0499	45.50788	NM_145467	integrin, beta-like 1
Dtl	0.49972245	P	321.278	642.9135	NM_029766	denticleless homolog (Drosophila)
Cdc6	0.4993686	P	64.1596	128.4814	NM_011799	cell division cycle 6 homolog (S. cerevisiae)
Gm5841	0.4979548	P	704.559	1414.905	XR_030927	predicted gene 5841
Hist2h4	0.49793872	P	11790.8	23679.26	NM_033596	histone cluster 2, H4
Shcbp1	0.49730983	P	723.736	1455.301	NM_011369	Shc SH2-domain binding protein 1
Incenp	0.4971604	P	3783.81	7610.849	NM_016692	inner centromere protein
Pcdhb17	0.49672756	P	137.088	275.9815	NM_053142	protocadherin beta 17

	0.4966828	P	14.2428	28.67575		
Hist1h4k	0.49663603	P	12007.8	24178.27	NM_178211	histone cluster 1, H4k
Adcy4	0.49591085	P	82.1981	165.7518	NM_080435	adenylate cyclase 4
Tmem90b	0.4958307	P	19.1504	38.6229	NM_001085521	transmembrane protein 90B
Slc16a10	0.49572888	P	86.4824	174.455	NM_001114332	solute carrier family 16 (monocarboxylic acid transporters), member 10
Gm14008	0.49509957	P	1285.56	2596.569	XR_034868	predicted gene 14008
Aspm	0.49428004	P	400.525	810.3198	NM_009791	asp (abnormal spindle)-like, microcephaly associated (Drosophila)
Il1rl1	0.49322978	P	1888.55	3828.941	NM_001025602	interleukin 1 receptor-like 1
Hmx1	0.4931395	P	21.2157	43.02172	NM_010445	H6 homeobox 1
Thbs2	0.49293908	P	2792.71	5665.428	NM_011581	thrombospondin 2
Hist1h2af	0.49288434	P	51800.3	105096.3	NM_175661	histone cluster 1, H2af
Engase	0.49233502	P	223.114	453.1747	NM_172573	endo-beta-N-acetylglucosaminidase
	0.4919613	P	18.4116	37.42496	XR_035139	
A730089K16Rik	0.49193168	P	88.4714	179.8449	XR_035181	RIKEN cDNA A730089K16 gene
A430033K04Rik	0.49166065	P	23.9727	48.75867	NM_183025	RIKEN cDNA A430033K04 gene
Pcsk9	0.49161324	P	138.045	280.8004	NM_153565	proprotein convertase subtilisin/kexin type 9
Zfp101	0.49161306	P	68.3878	139.109	NM_009542	zinc finger protein 101
Tpx2	0.49147272	P	2253.72	4585.652	NM_028109	TPX2, microtubule-associated protein homolog (Xenopus laevis)
Dlgap5	0.49132037	P	145.235	295.6012	NM_144553	discs, large (Drosophila) homolog-associated protein 5
Ccnb2	0.49076512	P	3726.33	7592.889	NM_007630	cyclin B2
1190002F15Rik	0.49068838	P	476.546	971.178	XM_001481164	RIKEN cDNA 1190002F15 gene
2410075B13Rik	0.4903011	P	131.25	267.6935	NM_001163518	RIKEN cDNA 2410075B13 gene
Herc5	0.4902201	P	28.1072	57.33579	NM_025992	hect domain and RLD 5
	0.49007714	P	132.218	269.7896	BC036719	
B930036G03Rik	0.48969647	P	258.086	527.0316	AK047211	RIKEN cDNA B930036G03 gene
Gm6439	0.48925155	P	13.1349	26.84686	XR_032293	predicted gene 6439
Padi2	0.48906296	P	27.3655	55.95486	NM_008812	peptidyl arginine deiminase, type II
Rad51ap1	0.48866037	P	683.689	1399.109	NM_009013	RAD51 associated protein 1
Dck	0.48788133	P	184.916	379.018	NM_007832	deoxycytidine kinase

Tst	0.48765182	P	124.93	256.1871	NM_009437	thiosulfate sulfurtransferase, mitochondrial
Tmtc1	0.48701298	P	17.2915	35.50522	NM_198967	transmembrane and tetratricopeptide repeat containing 1
Cdca8	0.48695883	P	3777.78	7757.906	NM_026560	cell division cycle associated 8
LOC641235	0.4869275	P	31.1561	63.98509	XM_918592	similar to H-2 class I histocompatibility antigen, D-37 alpha chain precursor
Utp14b	0.48684704	P	21.9526	45.09133	NM_001001981	UTP14, U3 small nucleolar ribonucleoprotein, homolog B (yeast)
Dlgap5	0.4867912	P	148.829	305.7346	NM_144553	discs, large (Drosophila) homolog-associated protein 5
LOC100048743	0.4865527	P	108.039	222.0506	XM_001481106	similar to FK506 binding protein 4
Lmnb1	0.48611605	P	1270.96	2614.519	NM_010721	lamin B1
Cit	0.48603314	P	42.7907	88.04079	NM_007708	citron
Areg	0.48583707	P	24.8438	51.13615	NM_009704	amphiregulin
Dtx3l	0.485627	P	119.983	247.0686	NM_001013371	deltex 3-like (Drosophila)
Fam54a	0.48533884	P	16.3202	33.6263	NM_027930	family with sequence similarity 54, member A
Tgfbr3	0.4852287	P	249.557	514.308	NM_011578	transforming growth factor, beta receptor III
Idh2	0.48491618	P	8468.09	17462.99	NM_173011	isocitrate dehydrogenase 2 (NADP+), mitochondrial
Gm8288	0.48467097	P	26.3036	54.2711	XR_034140	predicted gene 8288
Igfbp5	0.4846145	P	1241.8	2562.455	NM_010518	insulin-like growth factor binding protein 5
Nsl1	0.48460764	P	399.112	823.5775	NM_198654	NSL1, MIND kinetochore complex component, homolog (S. cerevisiae)
2700099C18Rik	0.48436597	P	113.609	234.5524	NR_024720	NDC80 homolog, kinetochore complex component pseudogene
Iqgap3	0.48389894	P	297.322	614.4306	NM_001033484	IQ motif containing GTPase activating protein 3
Lgals3bp	0.4828892	P	1569.71	3250.659	NM_011150	lectin, galactoside-binding, soluble, 3 binding protein
Gm16039	0.48280656	P	18.7623	38.86088	NR_033518	predicted gene 16039
Polq	0.48267952	P	102.383	212.1145	NM_029977	polymerase (DNA directed), theta
Orc1l	0.48251736	P	82.3597	170.6875	NM_011015	origin recognition complex, subunit 1-like (S.cereviaiae)
4930452B06Rik	0.48231107	P	45.1931	93.70121	NM_028934	RIKEN cDNA 4930452B06 gene
Tmem204	0.48182708	P	55.1218	114.4017	NM_001001183	transmembrane protein 204
Crygn	0.4816385	P	12.7248	26.41979	NM_153076	crystallin, gamma N
Gm4248	0.48157063	P	1644.71	3415.3	XM_001479553	high mobility group nucleosomal binding domain 2 pseudogene
Nsg1	0.4809481	P	2321.81	4827.565	NM_010942	neuron specific gene family member 1
Fignl1	0.48084643	P	869.712	1808.71	NM_001163359	fidgetin-like 1

Zfp395	0.47972265	P	39.0331	81.366	NM_199029	zinc finger protein 395
Gm8681	0.47968957	P	1462.11	3048.025	XR_032130	predicted gene 8681
Arc	0.47956118	P	262.907	548.2247	NM_018790	activity regulated cytoskeletal-associated protein
4921501E09Rik	0.47834906	P	13.6393	28.5133	NM_001009544	RIKEN cDNA 4921501E09 gene
Pola2	0.4779929	P	33.5347	70.15734	NM_008893	polymerase (DNA directed), alpha 2
Hmgb2	0.47788644	P	3109.43	6506.619	NM_008252	high mobility group box 2
Tarsl2	0.47753054	P	188.988	395.76	NM_172310	threonyl-tRNA synthetase-like 2
Nap1l5	0.47738943	P	42.3596	88.73183	NM_021432	nucleosome assembly protein 1-like 5
Cass4	0.47650072	P	13.7963	28.95333	NM_001033538	Cas scaffolding protein family member 4
Kif23	0.47645277	P	541.326	1136.158	NM_024245	kinesin family member 23
	0.4761326	P	17.421	36.58846	AK145208	
Gm3970	0.47505537	P	43.4524	91.46813	XM_001479374	predicted gene 3970
Slc1a5	0.47497112	P	490.665	1033.043	NM_009201	solute carrier family 1 (neutral amino acid transporter), member 5
Rbl1	0.47495517	P	250.131	526.6419	NM_011249	retinoblastoma-like 1 (p107)
Fat3	0.47484028	P	14.3405	30.2007	NM_001080814	FAT tumor suppressor homolog 3 (Drosophila)
Fbxl16	0.47477123	P	112.17	236.2614	NM_001164225	F-box and leucine-rich repeat protein 16
2700094K13Rik	0.47469217	P	4313.53	9087.002	NM_001037279	RIKEN cDNA 2700094K13 gene
Ncaph	0.4735427	P	1696.81	3583.223	NM_144818	non-SMC condensin I complex, subunit H
Hist1h4j	0.47307417	P	598.2	1264.496	NM_178210	histone cluster 1, H4j
Nav2	0.47273752	P	15.0954	31.93191	AK129480	neuron navigator 2
Fndc1	0.47267526	P	37.2875	78.8861	NM_001081416	fibronectin type III domain containing 1
Kntc1	0.4723867	P	404.925	857.1888	NM_001042421	kinetochore associated 1
Apitd1	0.47227302	P	91.4387	193.6142	NM_027263	apoptosis-inducing, TAF9-like domain 1
Rab40b	0.4719714	P	82.8888	175.6225	NM_139147	Rab40b, member RAS oncogene family
Zc3hav1	0.47176003	P	189.436	401.5522	NM_028864	zinc finger CCCH type, antiviral 1
Kcnab3	0.471481	P	42.1185	89.33231	NM_010599	potassium voltage-gated channel, shaker-related subfamily, beta member 3
Atp10a	0.4711684	P	82.3226	174.7201	NM_009728	ATPase, class V, type 10A
Gas1	0.4709717	P	193.268	410.3593	NM_008086	growth arrest specific 1
Lrrn3	0.4707392	P	164.367	349.1679	NM_010733	leucine rich repeat protein 3, neuronal

Heyl	0.47015253	P	64.65	137.5085	NM_013905	hairy/enhancer-of-split related with YRPW motif-like
Arhgap19	0.47006813	P	31.4051	66.80965	NM_027667	Rho GTPase activating protein 19
Prc1	0.46995077	P	3931.56	8365.896	NM_145150	protein regulator of cytokinesis 1
Gas2l3	0.4696138	P	88.2641	187.9505	NM_001079876	growth arrest-specific 2 like 3
Stmn1-rs2	0.46944517	P	1149.72	2449.097	XR_034760	stathmin 1, related sequence 2
Brip1	0.4692454	P	41.1552	87.70503	NM_178309	BRCA1 interacting protein C-terminal helicase 1
Nt5dc2	0.4690144	P	241.349	514.5867	NM_027289	5'-nucleotidase domain containing 2
	0.4684129	P	12.1972	26.03936	BC051161	
Obsl1	0.4676715	P	102.738	219.6794	NM_178884	obscurin-like 1
Gm9430	0.4674049	P	80.4546	172.1304	XR_035696	predicted gene 9430
Racgap1	0.4673257	P	1349.15	2886.961	NM_012025	Rac GTPase-activating protein 1
LOC547349	0.46722642	P	27.0638	57.92442	NM_001025208	similar to MHC class I antigen precursor
Hist2h2ac	0.46633378	P	49191.3	105485.3	NM_175662	histone cluster 2, H2ac
Wdr31	0.46608666	P	21.9629	47.12182	NM_023597	WD repeat domain 31
Npdc1	0.46589395	P	5030.04	10796.54	NM_008721	neural proliferation, differentiation and control gene 1
Ube2c	0.46576387	P	2231.79	4791.679	NM_026785	ubiquitin-conjugating enzyme E2C
	0.465657	P	18.9527	40.70107	AK076869	
Mapk12	0.46542066	P	269.495	579.0344	NM_013871	mitogen-activated protein kinase 12
Tmpo	0.46535915	P	231.608	497.6982	NM_001080129	thymopoietin
Slc6a17	0.4652994	P	42.7821	91.94525	NM_172271	solute carrier family 6 (neurotransmitter transporter), member 17
Gm2973	0.46521136	P	18.5856	39.95087	XM_001475294	predicted gene 2973
Tox	0.4650599	P	57.3157	123.2438	NM_145711	thymocyte selection-associated high mobility group box
Nlrc5	0.4647163	P	29.1688	62.76679	NM_001033207	NLR family, CARD domain containing 5
Sgol1	0.4645085	P	867.304	1867.144	NM_028232	shugoshin-like 1 (S. pombe)
Adam12	0.46398282	P	25.8719	55.76039	NM_007400	a disintegrin and metallopeptidase domain 12 (meltrin alpha)
Ifi27l1	0.4635289	P	6057.31	13067.82	NM_026790	interferon, alpha-inducible protein 27 like 1
Slc27a6	0.463089	P	13.4769	29.10212	NM_001081072	solute carrier family 27 (fatty acid transporter), member 6
F630043A04Rik	0.46307236	P	107.132	231.3505	NM_198605	RIKEN cDNA F630043A04 gene
Chaf1b	0.46298847	P	2145.63	4634.305	NM_028083	chromatin assembly factor 1, subunit B (p60)

Agrn	0.46261263	P	1882.08	4068.374	NM_021604	agrin
Nmral1	0.4625752	P	127.777	276.2293	NM_026393	NmrA-like family domain containing 1
Gprc5c	0.46225908	P	30.0943	65.10271	NM_001110337	G protein-coupled receptor, family C, group 5, member C
LOC675032	0.46217883	P	33.6748	72.86102	XM_983011	similar to Exosome complex exonuclease RRP45
Acot4	0.46199426	P	15.0568	32.59079	NM_134247	acyl-CoA thioesterase 4
4933402N22Rik	0.4617811	P	15.0451	32.58048	NM_001177510	RIKEN cDNA 4933402N22 gene
Rbm14	0.46141115	P	66.8704	144.9259	NM_019869	RNA binding motif protein 14
Ppil5	0.46087155	P	110.527	239.8224	NM_001081406	peptidylprolyl isomerase (cyclophilin) like 5
Hdac1	0.46065068	P	3284.36	7129.836	NM_008228	histone deacetylase 1
2210411K11Rik	0.46039665	P	159.396	346.2143	NM_029384	RIKEN cDNA 2210411K11 gene
Fam83e	0.4597781	P	19.0832	41.50528	NM_001033170	family with sequence similarity 83, member E
Tube1	0.4597731	P	91.339	198.661	NM_028006	epsilon-tubulin 1
Cdca3	0.45976642	P	2185.86	4754.29	NM_013538	cell division cycle associated 3
Has1	0.45947307	P	14.2076	30.92153	NM_008215	hyaluronan synthase1
Mki67	0.4591325	P	1856.79	4044.12	NM_001081117	antigen identified by monoclonal antibody Ki 67
Ttk	0.45894602	P	28.4651	62.02283	NM_009445	Ttk protein kinase
Cdca5	0.45881757	P	726.31	1583.004	NM_026410	cell division cycle associated 5
Hist1h2ak	0.45873538	P	48152.9	104968.7	NM_178183	histone cluster 1, H2ak
Ifi203	0.4586008	P	15.2468	33.24632	NM_001045481	interferon activated gene 203
Gm8850	0.45768806	P	235.324	514.1583	XR_002254	predicted gene 8850
Tnfsf11	0.4574774	P	24.1293	52.74429	NM_011613	tumor necrosis factor (ligand) superfamily, member 11
Trip13	0.45711368	P	1238.51	2709.406	NM_027182	thyroid hormone receptor interactor 13
4930427A07Rik	0.4567689	P	401.56	879.1312	NM_134041	RIKEN cDNA 4930427A07 gene
Ccna2	0.45638034	P	1357.14	2973.713	NM_009828	cyclin A2
Mlf1ip	0.45622918	P	178.425	391.0861	NM_027973	myeloid leukemia factor 1 interacting protein
Flt1	0.45617583	P	1655.54	3629.169	NM_010228	FMS-like tyrosine kinase 1
Gm11545	0.45567408	P	28.0494	61.55584	NM_001105561	predicted gene 11545
Hmgn2l6	0.45548207	P	3280.91	7203.158	XM_001472085	high-mobility group nucleosomal binding domain 2-like 6
Rfc5	0.4550176	P	1278.61	2810.019	NM_028128	replication factor C (activator 1) 5

Traip	0.454836	P	345.693	760.0381	NM_011634	TRAF-interacting protein
Slc2a6	0.4547584	P	174.222	383.1082	NM_172659	solute carrier family 2 (facilitated glucose transporter), member 6
Cdt1	0.4537437	P	2134.57	4704.35	NM_026014	chromatin licensing and DNA replication factor 1
Gsg2	0.45310393	P	130.525	288.0695	NM_010353	germ cell-specific gene 2
Mcm10	0.45251307	P	525.475	1161.238	NM_027290	minichromosome maintenance deficient 10 (S. cerevisiae)
Ccnb1	0.4524118	P	249.279	551.0011	NM_172301	cyclin B1
Gm8956	0.4521483	P	2414.18	5339.35	XR_030853	predicted gene 8956
Padi2	0.45200574	P	28.4513	62.94449	NM_008812	peptidyl arginine deiminase, type II
Cenph	0.4519262	P	444.754	984.1291	NM_021886	centromere protein H
Flywch2	0.45147333	P	144.013	318.9845	NM_029798	FLYWCH family member 2
S1pr3	0.4513705	P	4508.93	9989.421	NM_010101	sphingosine-1-phosphate receptor 3
Cdca8	0.45068038	P	290.753	645.1426	NM_026560	cell division cycle associated 8
Xkr5	0.45054692	P	20.1499	44.72318	NM_176951	X Kell blood group precursor-related family, member 5
Poc1a	0.44977888	P	1013.73	2253.832	NM_027354	POC1 centriolar protein homolog A (Chlamydomonas)
A530064D06Rik	0.44891396	P	20.3129	45.24898	NM_178796	RIKEN cDNA A530064D06 gene
Hist1h2ah	0.44837826	P	54810	122240.5	NM_175659	histone cluster 1, H2ah
Gm6594	0.4468498	P	8918.84	19959.37	XM_890094	predicted pseudogene 6594
Pdzd4	0.4463961	P	134.057	300.3089	NM_001029868	PDZ domain containing 4
D030062O11Rik	0.4463192	P	20.6844	46.34443	AK141776	RIKEN cDNA D030062O11 gene
Ect2	0.44570217	P	704.657	1581.005	NM_007900	ect2 oncogene
Hist1h4d	0.4456519	P	5577.56	12515.51	NM_175654	histone cluster 1, H4d
Mycn	0.4456322	P	27.9947	62.82017	NM_008709	v-myc myelocytomatosis viral related oncogene, neuroblastoma derived (avian)
Bub1	0.44531968	P	314.056	705.2365	NM_009772	budding uninhibited by benzimidazoles 1 homolog (S. cerevisiae)
Lama4	0.44530264	P	605.169	1359.007	NM_010681	laminin, alpha 4
	0.44503623	P	22.637	50.86548	XM_001473004	
Kcne4	0.44475865	P	76.7737	172.6187	NM_021342	potassium voltage-gated channel, Isk-related subfamily, gene 4
Hmgn2	0.44438723	P	478.666	1077.138	NM_016957	high mobility group nucleosomal binding domain 2
Pole2	0.44396266	P	196.807	443.2951	NM_011133	polymerase (DNA directed), epsilon 2 (p59 subunit)
AI414108	0.44388595	P	65.5684	147.7144	NR_027907	expressed sequence AI414108

Stmn1	0.4433423	P	9693.32	21864.19	NM_019641	stathmin 1
5730590G19Rik	0.44332653	P	198.075	446.7925	NM_029835	RIKEN cDNA 5730590G19 gene
Lin9	0.44312724	P	307.168	693.1815	NM_001103182	lin-9 homolog (C. elegans)
Fam109b	0.44288194	P	49.2023	111.0958	NM_177391	family with sequence similarity 109, member B
Fbxo5	0.44256443	P	682.469	1542.078	NM_025995	F-box protein 5
	0.4425433	P	23.269	52.58008	XM_001000510	
Spag5	0.44216937	P	621.269	1405.048	NM_017407	sperm associated antigen 5
Nuf2	0.44142845	P	324.085	734.1729	NM_023284	NUF2, NDC80 kinetochore complex component, homolog (S. cerevisiae)
Sgol2	0.44094688	P	81.4891	184.8047	NM_199007	shugoshin-like 2 (S. pombe)
Plk1	0.44086808	P	4638.79	10521.94	NM_011121	polo-like kinase 1 (Drosophila)
Slc1a5	0.4408405	P	100.543	228.0709	NM_009201	solute carrier family 1 (neutral amino acid transporter), member 5
Fanci	0.4408077	P	165.275	374.9361	NM_145946	Fanconi anemia, complementation group I
Cdca2	0.4402583	P	202.397	459.7224	NM_175384	cell division cycle associated 2
Mamdc4	0.4402066	P	20.182	45.84663	NM_001081199	MAM domain containing 4
Chadl	0.43989643	P	258.59	587.8431	NM_001164320	chondroadherin-like
Ndc80	0.43962938	P	179.284	407.8071	NM_023294	NDC80 homolog, kinetochore complex component (S. cerevisiae)
Cep55	0.4395659	P	31.4638	71.57916	NM_028760	centrosomal protein 55
Nmral1	0.43895248	P	388.685	885.484	NM_026393	NmrA-like family domain containing 1
Cenpp	0.43881404	P	287.794	655.8452	NM_025495	centromere protein P
Dynlt1d	0.43858564	P	19.5224	44.51213	XM_001475631	dynein light chain Tctex-type 1D
Xrcc2	0.43842867	P	24.8515	56.68313	NM_020570	X-ray repair complementing defective repair in Chinese hamster cells 2
	0.4382792	P	47.8009	109.0649	AK014764	
Rtp4	0.4382689	P	36.8243	84.02226	NM_023386	receptor transporter protein 4
Prodh	0.43752393	P	55.5754	127.0226	NM_011172	proline dehydrogenase
Lass4	0.43722346	P	816.869	1868.31	NM_026058	LAG1 homolog, ceramide synthase 4
	0.43700394	P	13.8397	31.66939	XM_001472339	
Hmgb3	0.43677217	P	844.62	1933.777	NM_008253	high mobility group box 3
Tacc3	0.4367059	P	4123.88	9443.146	NM_001040435	transforming, acidic coiled-coil containing protein 3
Ramp3	0.4366696	P	2423.31	5549.537	NM_019511	receptor (calcitonin) activity modifying protein 3

Kazald1	0.4366025	P	332.175	760.8176	NM_178929	Kazal-type serine peptidase inhibitor domain 1
Gm11223	0.4363997	P	8549.05	19589.94	XM_001474074	stathmin 1 pseudogene
Cdkn2c	0.4363479	P	193.544	443.555	NM_007671	cyclin-dependent kinase inhibitor 2C (p18, inhibits CDK4)
Egr2	0.43576416	P	323.146	741.5613	NM_010118	early growth response 2
Metrn	0.43561506	P	601.023	1379.711	NM_133719	meteorin, glial cell differentiation regulator
Pde4b	0.43531272	P	317.829	730.1172	NM_019840	phosphodiesterase 4B, cAMP specific
4930427A07Rik	0.4346546	P	734.802	1690.543	NM_134041	RIKEN cDNA 4930427A07 gene
Ccdc99	0.43421072	P	91.2123	210.0646	NM_027411	coiled-coil domain containing 99
Mki67	0.43410644	P	2732.49	6294.516	NM_001081117	antigen identified by monoclonal antibody Ki 67
Gm5771	0.43406904	P	22.5696	51.99532	NM_001038997	predicted gene 5771
Ldb2	0.43394154	P	406.673	937.1606	NM_001077398	LIM domain binding 2
Kif4	0.4334481	P	274.947	634.3242	NM_008446	kinesin family member 4
Rfx3	0.4329309	P	13.495	31.17126	NM_011265	regulatory factor X, 3 (influences HLA class II expression)
Uhrf1	0.43242666	P	43.8168	101.3278	NM_001111079	ubiquitin-like, containing PHD and RING finger domains, 1
Ccnb1	0.43194354	P	223.091	516.481	NM_172301	cyclin B1
Timeless	0.4313497	P	53.9643	125.1056	NM_001164081	timeless homolog (Drosophila)
Ncapg	0.43120283	P	20.4606	47.44999	NM_019438	non-SMC condensin I complex, subunit G
Zcchc3	0.43118653	P	95.9082	222.4286	NM_175126	zinc finger, CCHC domain containing 3
Depdc1a	0.4308744	P	95.2186	220.9891	NM_001172092	DEP domain containing 1a
6230427J02Rik	0.43085155	P	97.3531	225.9551	NM_026597	RIKEN cDNA 6230427J02 gene
AI429363	0.43034133	P	32.4791	75.47276	CF532098	expressed sequence AI429363
Gm4870	0.43022707	P	189.627	440.7605	XR_033857	predicted gene 4870
Dhfr	0.42979404	P	44.3053	103.085	NM_010049	dihydrofolate reductase
Bub1b	0.4294328	P	275.619	641.8215	NM_009773	budding uninhibited by benzimidazoles 1 homolog, beta (S. cerevisiae)
Ccnf	0.42927566	P	1147.26	2672.539	NM_007634	cyclin F
1520402A15Rik	0.42921206	P	79.2258	184.5842	NM_026547	RIKEN cDNA 1520402A15 gene
Herc5	0.42879486	P	21.0613	49.11731	NM_025992	hect domain and RLD 5
Kif20a	0.42861834	P	2264.47	5283.195	NM_009004	kinesin family member 20A
Lilra6	0.42836502	P	54.4709	127.16	NM_011090	leukocyte immunoglobulin-like receptor, subfamily A (with TM domain), member 6

Ube2c	0.42730278	P	2612.05	6112.869	NM_026785	ubiquitin-conjugating enzyme E2C
Foxm1	0.4271506	P	34.2756	80.24245	NM_008021	forkhead box M1
	0.42698786	P	20658	48380.68		
Ercc6l	0.42670947	P	127.33	298.4002	NM_146235	excision repair cross-complementing group 6 - like
Igsf10	0.42576817	P	37.3537	87.73258	NM_001162884	immunoglobulin superfamily, member 10
Adamts2	0.42537674	P	1274.11	2995.244	NM_175643	a disintegrin-like and metallopeptidase with thrombospondin type 1 motif, 2
Dscc1	0.42519876	P	91.0499	214.135	NM_183089	defective in sister chromatid cohesion 1 homolog (S. cerevisiae)
	0.42431313	P	16.4387	38.74181	AK034565	
Oas1e	0.4243004	P	14.8017	34.88502	NM_145210	2'-5' oligoadenylate synthetase 1E
Fam64a	0.42427373	P	773.261	1822.553	NM_144526	family with sequence similarity 64, member A
Cdo1	0.42344025	P	842.796	1990.353	NM_033037	cysteine dioxygenase 1, cytosolic
Oip5	0.42307237	P	150.539	355.8231	NM_001042653	Opa interacting protein 5
LOC100046186	0.42270666	P	1836.73	4345.16	XM_001475752	similar to receptor activity modifying protein 3
Mmp13	0.4226668	P	34.9353	82.65443	NM_008607	matrix metallopeptidase 13
Aurka	0.42219508	P	2858.36	6770.23	NM_011497	aurora kinase A
Flt3l	0.4212682	P	54.0529	128.31	NM_013520	FMS-like tyrosine kinase 3 ligand
Mybl2	0.42081523	P	252.124	599.1328	NM_008652	myeloblastosis oncogene-like 2
Pbk	0.42078012	P	457.29	1086.767	NM_023209	PDZ binding kinase
Gp6	0.4206527	P	16.017	38.07657	NM_001163014	glycoprotein 6 (platelet)
Pcdhb18	0.4199611	P	17.3035	41.20262	NM_053143	protocadherin beta 18
Cdc25b	0.4194921	P	557.337	1328.6	NM_023117	cell division cycle 25 homolog B (S. pombe)
D2Ertd750e	0.41937906	P	301.106	717.9808	NM_026412	DNA segment, Chr 2, ERATO Doi 750, expressed
Cenpi	0.4190777	P	419.667	1001.407	NM_145924	centromere protein I
B3gnt3	0.4190259	P	16.9591	40.47278	NM_028189	UDP-GlcNAc:betaGal beta-1,3-N-acetylglucosaminyltransferase 3
Cdkn1b	0.4184845	P	25.6665	61.33192	AK050240	cyclin-dependent kinase inhibitor 1B
Dhx58	0.41816235	P	22.1487	52.96675	NM_030150	DEXH (Asp-Glu-X-His) box polypeptide 58
Fbxo32	0.4181162	P	193.741	463.367	NM_026346	F-box protein 32
Cdk5rap1	0.41769654	P	90.6743	217.0819	NM_025876	CDK5 regulatory subunit associated protein 1
Exo1	0.4176557	P	583.001	1395.89	NM_012012	exonuclease 1

Spn	0.41728434	P	78.5252	188.1815	NM_009259	sialophorin
Tmpo	0.41717556	P	1260.19	3020.769	NM_001080129	thymopoietin
	0.41697	P	199.127	477.5569	U07661	
Gm4870	0.41654506	P	433.227	1040.048	XR_033857	predicted gene 4870
Olfr220	0.41646522	P	28.4981	68.42844	NM_207694	olfactory receptor 220
Ube2t	0.4160433	P	58.6363	140.9379	NM_026024	ubiquitin-conjugating enzyme E2T (putative)
Crabp1	0.4146929	P	4999.53	12055.97	NM_013496	cellular retinoic acid binding protein I
Traip	0.41465506	P	38.7791	93.52127	NM_011634	TRAF-interacting protein
Zfp185	0.41460782	P	16.7982	40.51588	NM_009549	zinc finger protein 185
Gm8284	0.41458133	P	289.389	698.0266	XR_030646	predicted gene 8284
Hmgn2	0.41436446	P	1737.46	4193.065	NM_016957	high mobility group nucleosomal binding domain 2
Gm5593	0.4142781	P	1846.2	4456.417	XM_485921	predicted gene 5593
Tmem22	0.4142267	P	50.3639	121.5853	NM_001101483	transmembrane protein 22
Gpm6b	0.41383633	P	133.09	321.6006	NM_001177956	glycoprotein m6b
Slc25a35	0.41379014	P	10.9216	26.39406	NM_028048	solute carrier family 25, member 35
Melk	0.41340607	P	552.901	1337.429	NM_010790	maternal embryonic leucine zipper kinase
Gm16510	0.41318482	P	956.611	2315.213	XM_001473460	predicted pseudogene 16510
LOC631505	0.41302836	P	70.0216	169.5322	XM_905344	similar to High mobility group protein 4 (HMG-4)
Rad54l	0.41237652	P	92.1097	223.3632	NM_009015	RAD54 like (S. cerevisiae)
Tshz3	0.4118239	P	832.64	2021.836	NM_172298	teashirt zinc finger family member 3
Pkp1	0.41176784	P	47.2741	114.8076	NM_019645	plakophilin 1
Gm10105	0.41155544	P	302.641	735.3593	XM_001477902	high mobility group nucleosomal binding domain 2 pseudogene
Tmod2	0.41151208	P	13.505	32.81794	NM_001038710	tropomodulin 2
Gm5899	0.4111551	P	959.366	2333.343	XM_620527	predicted pseudogene 5899
Chaf1b	0.4107854	P	948.798	2309.717	NM_028083	chromatin assembly factor 1, subunit B (p60)
Vdr	0.41073942	P	194.352	473.1766	NM_009504	vitamin D receptor
Sfrp1	0.41051498	P	46.2784	112.7325	NM_013834	secreted frizzled-related protein 1
H2afy3	0.41009343	P	1024.7	2498.687	NR_003523	H2A histone family, member Y3
Rfx7	0.4100544	P	22.8389	55.69722	NM_001033536	regulatory factor X, 7

Ikzf4	0.40972868	P	37.3987	91.2767	NM_011772	IKAROS family zinc finger 4
Kif11	0.40969062	P	134.675	328.7239	NM_010615	kinesin family member 11
4930547N16Rik	0.40958494	P	172.432	420.9928	NM_029249	RIKEN cDNA 4930547N16 gene
Clspn	0.40949637	P	74.237	181.2885	NM_175554	claspin homolog (Xenopus laevis)
Prss1	0.4091965	P	18.567	45.37432	NM_053243	protease, serine, 1 (trypsin 1)
Enox1	0.40915155	P	31.1754	76.19529	NM_172813	ecto-NOX disulfide-thiol exchanger 1
Ppil5	0.4084273	P	96.0531	235.1779	NM_001081406	peptidylprolyl isomerase (cyclophilin) like 5
Apol10b	0.40756747	P	12.6198	30.96358	NM_177820	apolipoprotein L 10b
Ccl12	0.40704298	P	115.076	282.7114	NM_011331	chemokine (C-C motif) ligand 12
Cdc25c	0.4068412	P	69.8314	171.6429	NM_009860	cell division cycle 25 homolog C (S. pombe)
Mex3b	0.40589884	P	27.5556	67.88783	NM_175366	mex3 homolog B (C. elegans)
Gm12387	0.40584975	P	234.506	577.814	XR_032698	predicted gene 12387
Lrrc15	0.4057469	P	4103.22	10112.75	NM_028973	leucine rich repeat containing 15
LOC638183	0.40562436	P	308.302	760.0685	XR_034120	similar to High mobility group protein 2 (HMG-2)
Gm10270	0.4050981	P	373.607	922.2618	XM_001474128	predicted pseudogene 10270
Sept4	0.40503544	P	26.2565	64.82514	NM_011129	septin 4
Prim1	0.40501207	P	1195.83	2952.589	NM_008921	DNA primase, p49 subunit
Crip1	0.40470853	P	4801.96	11865.22	NM_007763	cysteine-rich protein 1 (intestinal)
Gcat	0.4045735	P	772.982	1910.609	NM_013847	glycine C-acetyltransferase (2-amino-3-ketobutyrate-coenzyme A ligase)
2810405K02Rik	0.40399957	P	1011.54	2503.805	NM_025582	RIKEN cDNA 2810405K02 gene
LOC100046632	0.40388656	P	14.3758	35.59373	XM_001476227	similar to tripartite motif protein TRIM30
Ndst3	0.40326077	P	41.5104	102.9369	NM_031186	N-deacetylase/N-sulfotransferase (heparan glucosaminyl) 3
Il1b	0.40233412	P	24.7642	61.55143	NM_008361	interleukin 1 beta
LOC100044832	0.40208116	P	33.6243	83.62563	XM_001472830	similar to RIKEN cDNA 1810030J14 gene
Gm10105	0.40043795	P	132.026	329.704	XM_001477902	high mobility group nucleosomal binding domain 2 pseudogene
	0.39968434	P	12317.7	30818.59	AF064749	
Lrp8	0.39922625	P	26.839	67.22746	NM_001080926	low density lipoprotein receptor-related protein 8, apolipoprotein e receptor
Tmem176a	0.3989814	P	196.219	491.8006	NM_025326	transmembrane protein 176A
Egr1	0.3974166	P	3035.82	7638.883	NM_007913	early growth response 1

Igsf10	0.39736304	P	95.3232	239.8893	NM_001162884	immunoglobulin superfamily, member 10
2610318N02Rik	0.39732856	P	177.97	447.9152	NM_183287	RIKEN cDNA 2610318N02 gene
Sgsm1	0.3964008	P	16.5937	41.86089	NM_172718	small G protein signaling modulator 1
Adarb1	0.3963654	P	305.512	770.7835	NM_001024837	adenosine deaminase, RNA-specific, B1
Pira11	0.3961627	P	40.3329	101.809	NM_011088	paired-Ig-like receptor A11
Tmem176b	0.3959107	P	133.408	336.9651	NM_023056	transmembrane protein 176B
Ccnb1	0.39530966	P	1604.87	4059.782	NM_172301	cyclin B1
Mrgprf	0.39510146	P	103.695	262.4506	NM_145379	MAS-related GPR, member F
Prr11	0.3946984	P	136.391	345.5565	NM_175563	proline rich 11
Ccdc69	0.39443952	P	24.0726	61.02981	NM_177471	coiled-coil domain containing 69
Cdkn3	0.39434528	P	283.494	718.8979	NM_028222	cyclin-dependent kinase inhibitor 3
Ror2	0.3940472	P	772.663	1960.838	NM_013846	receptor tyrosine kinase-like orphan receptor 2
Gm3247	0.39379892	P	34.5969	87.85415	XM_001476088	predicted gene 3247
Recql4	0.3936404	P	139.051	353.2445	NM_058214	RecQ protein-like 4
Tcfap4	0.39342865	P	46.0637	117.0826	NM_031182	transcription factor AP4
Spc24	0.3931743	P	238.445	606.4606	NM_026282	SPC24, NDC80 kinetochore complex component, homolog (S. cerevisiae)
Bzrap1	0.39299256	P	27.7636	70.64661	NM_172449	benzodiazapine receptor associated protein 1
Asf1b	0.3929669	P	1146.31	2917.053	NM_024184	ASF1 anti-silencing function 1 homolog B (S. cerevisiae)
Fancd2	0.3921506	P	21.5097	54.85061	NM_001033244	Fanconi anemia, complementation group D2
Sh2d3c	0.38983375	P	234.912	602.5949	NM_013781	SH2 domain containing 3C
Ppfia3	0.38981605	P	99.4374	255.0881	NM_029741	protein tyrosine phosphatase, receptor type, f polypeptide, interacting protein, alpha 3
Top2a	0.3895629	P	718.493	1844.356	NM_011623	topoisomerase (DNA) II alpha
4833424O15Rik	0.38954815	P	13.5248	34.71928	NM_029425	RIKEN cDNA 4833424O15 gene
Ccne2	0.38948348	P	35.5451	91.26223	NM_001037134	cyclin E2
Gm5465	0.38869295	P	48.1067	123.7652	NM_001034882	predicted gene 5465
Nsl1	0.38691434	P	18.0789	46.72577	NM_198654	NSL1, MIND kinetochore complex component, homolog (S. cerevisiae)
Gpc2	0.38684434	P	13.9216	35.98763	NM_172412	glypican 2 (cerebroglycan)
Casc5	0.3867768	P	34.6267	89.52631	NM_029617	cancer susceptibility candidate 5
C330027C09Rik	0.3861406	P	18.604	48.17944	NM_172616	RIKEN cDNA C330027C09 gene

Mlf1ip	0.38606194	P	16.8764	43.71431	NM_027973	myeloid leukemia factor 1 interacting protein
BC053393	0.3845588	P	47.31	123.024	NM_001025435	cDNA sequence BC053393
Acot1	0.38319927	P	126.502	330.1194	NM_012006	acyl-CoA thioesterase 1
4930534B04Rik	0.38309494	P	36.1832	94.44969	NM_181815	RIKEN cDNA 4930534B04 gene
Hist2h2bb	0.38267145	P	347.865	909.043	NM_175666	histone cluster 2, H2bb
Hist1h2ak	0.3823249	P	4532.37	11854.76	NM_178183	histone cluster 1, H2ak
E2f8	0.38226345	P	274.172	717.2318	NM_001013368	E2F transcription factor 8
Hist1h2ag	0.38216662	P	40089	104899.1	NM_178186	histone cluster 1, H2ag
Col6a2	0.38126695	P	712.259	1868.136	NM_146007	collagen, type VI, alpha 2
Gm14548	0.3808579	P	45.6754	119.9276	NM_001166672	predicted gene 14548
Espl1	0.3807515	P	144.596	379.764	NM_001014976	extra spindle poles-like 1 (S. cerevisiae)
Pdgfra	0.3801102	P	513.577	1351.125	NM_011058	platelet derived growth factor receptor, alpha polypeptide
Dna2	0.37968132	P	41.8855	110.3174	NM_177372	DNA replication helicase 2 homolog (yeast)
Hs3st5	0.37933993	P	15.8804	41.86319	NM_001081208	heparan sulfate (glucosamine) 3-O-sulfotransferase 5
Nusap1	0.37851453	P	341.503	902.22	NM_133851	nucleolar and spindle associated protein 1
Ggt7	0.37752864	P	85.6004	226.7387	NM_144786	gamma-glutamyltransferase 7
Bst2	0.37750134	P	679.899	1801.052	NM_198095	bone marrow stromal cell antigen 2
Cdc6	0.37604216	P	146.201	388.7886	NM_011799	cell division cycle 6 homolog (S. cerevisiae)
Apcdd1	0.3737153	P	61.7299	165.1789	NM_133237	adenomatosis polyposis coli down-regulated 1
Gsdmd	0.37338936	P	219.545	587.9785	NM_026960	gasdermin D
Ptpru	0.3730085	P	53.8507	144.3686	NM_001083119	protein tyrosine phosphatase, receptor type, U
	0.37261257	P	299.242	803.0902		
Pcdhb21	0.3712713	P	66.153	178.1797	NM_053146	protocadherin beta 21
Birc5	0.37119082	P	775.787	2089.995	NM_009689	baculoviral IAP repeat-containing 5
Pde4b	0.3694214	P	85.1576	230.5162	NM_019840	phosphodiesterase 4B, cAMP specific
Fbln1	0.3691293	P	46.6048	126.2561	NM_010180	fibulin 1
Gm2862	0.36650756	P	261.129	712.4792	XR_031785	predicted gene 2862
Phf19	0.36634463	P	136.738	373.2502	NM_028716	PHD finger protein 19
Uhrf1	0.36593804	P	1128.36	3083.47	NM_010931	ubiquitin-like, containing PHD and RING finger domains, 1

Map4k1	0.36570963	P	43.459	118.8346	NM_008279	mitogen-activated protein kinase kinase kinase kinase 1
Zfp454	0.36530006	P	60.1454	164.6466	NM_172794	zinc finger protein 454
Fbln1	0.36527705	P	347.905	952.4425	NM_010180	fibulin 1
A230050P20Rik	0.3643708	P	91.3092	250.5941	NM_175687	RIKEN cDNA A230050P20 gene
Neil3	0.36425957	P	37.4312	102.7596	NM_146208	nei like 3 (E. coli)
Birc5	0.36417323	P	537.048	1474.704	NM_001012273	baculoviral IAP repeat-containing 5
Ptgfr	0.3640631	P	13.136	36.08171	NM_008966	prostaglandin F receptor
Cxcl10	0.36371222	P	64.2986	176.7843	NM_021274	chemokine (C-X-C motif) ligand 10
Ckap2l	0.36357445	P	197.752	543.9106	NM_181589	cytoskeleton associated protein 2-like
Gm6985	0.36245224	P	153.14	422.5103	NM_001177390	predicted pseudogene 6985
Kif2c	0.36157092	P	109.1	301.738	NM_134471	kinesin family member 2C
Klk5	0.3610609	P	11.1966	31.01037	NM_026806	kallikrein related-peptidase 5
Birc5	0.35944545	P	7700.6	21423.56	NM_001012273	baculoviral IAP repeat-containing 5
Igtp	0.35896033	P	104.506	291.135	NM_018738	interferon gamma induced GTPase
Gm5666	0.35846642	P	20.7485	57.88137	XR_034009	predicted gene 5666
Lrrc17	0.3580725	P	2068.88	5777.819	NM_028977	leucine rich repeat containing 17
Mxd3	0.3564356	P	77.9277	218.6304	NM_016662	Max dimerization protein 3
Esco2	0.35553515	P	31.0789	87.41451	NM_028039	establishment of cohesion 1 homolog 2 (S. cerevisiae)
Troap	0.35551617	P	34.476	96.97439	NM_030159	trophinin associated protein
Plk4	0.35526642	P	27.7177	78.01946	NM_011495	polo-like kinase 4 (Drosophila)
Gli2	0.3519407	P	37.195	105.6855	NM_001081125	GLI-Kruppel family member GLI2
Hist1h2an	0.3498761	P	32880	93976.14	NM_178184	histone cluster 1, H2an
	0.34966752	P	34689	99205.52	BC147161	
Nek2	0.3495931	P	137.777	394.1079	NM_010892	NIMA (never in mitosis gene a)-related expressed kinase 2
	0.3492712	P	45.1427	129.2482	AK149403	
Ctrc	0.34617567	P	17.7352	51.23185	NM_001033875	chymotrypsin C (caldecrin)
Kif2c	0.34607282	P	121.238	350.3248	NM_134471	kinesin family member 2C
Mfap4	0.34487948	P	2288.07	6634.414	NM_029568	microfibrillar-associated protein 4
Gstt1	0.34362227	P	66.9185	194.7444	NM_008185	glutathione S-transferase, theta 1

	0.34240204	P	141.776	414.0638	BC064680	
2810417H13Rik	0.34192827	P	89.2015	260.8779	NM_026515	RIKEN cDNA 2810417H13 gene
AI413759	0.34111115	P	25.3693	74.37244	BI134269	expressed sequence AI413759
Gm4961	0.3400524	P	15.0003	44.11158	XR_001540	predicted pseudogene 4961
	0.3396839	P	20.6462	60.7806	AK030813	
Depdc1b	0.33850327	P	149.082	440.4141	NM_178683	DEP domain containing 1B
	0.33848464	P	42.6227	125.9221	XR_032476	
Amigo1	0.33822832	P	23.7443	70.20198	NM_146137	adhesion molecule with Ig like domain 1
1810014F10Rik	0.33818558	P	163.012	482.02	NM_026928	RIKEN cDNA 1810014F10 gene
Hnf1a	0.3380637	P	14.3662	42.4956	M57966	HNF1 homeobox A
Fsd1	0.33727664	P	53.3502	158.1794	NM_183178	fibronectin type 3 and SPRY domain-containing protein
5830448L01Rik	0.33390725	P	14.0859	42.18504	XM_979896	RIKEN cDNA 5830448L01 gene
Slc43a1	0.33261967	P	58.3225	175.343	NM_001081349	solute carrier family 43, member 1
Oasl2	0.3317631	P	14.0973	42.49219	NM_011854	2'-5' oligoadenylate synthetase-like 2
Nkd2	0.33098164	P	91.6412	276.877	NM_028186	naked cuticle 2 homolog (Drosophila)
Gng7	0.3297834	P	38.4039	116.4518	NM_001038655	guanine nucleotide binding protein (G protein), gamma 7
1810014F10Rik	0.32905278	P	148.412	451.0288	NM_026928	RIKEN cDNA 1810014F10 gene
	0.3281105	P	24.2674	73.96109	BC025462	
Rbl1	0.32666945	P	25.602	78.37289	NM_001139516	retinoblastoma-like 1 (p107)
Tmpo	0.32539013	P	11.4495	35.18705	NM_001080134	thymopoietin
Shmt1	0.32346213	P	56.6698	175.1978	NM_009171	serine hydroxymethyltransferase 1 (soluble)
Rarres2	0.32265508	P	59.7747	185.2587	NM_027852	retinoic acid receptor responder (tazarotene induced) 2
Hist1h1e	0.31876075	P	2902.94	9106.955	NM_015787	histone cluster 1, H1e
Efna2	0.31830552	P	47.3673	148.8107	NM_007909	ephrin A2
Il1rl1	0.31739187	P	4065.58	12809.35	NM_010743	interleukin 1 receptor-like 1
Creg2	0.31738076	P	13.0504	41.1191	NM_170597	cellular repressor of E1A-stimulated genes 2
Kif15	0.31686118	P	36.3348	114.671	NM_010620	kinesin family member 15
Isg15	0.31567195	P	307.662	974.626	NM_015783	ISG15 ubiquitin-like modifier
Hpdl	0.31358275	P	43.7534	139.5275	NM_146256	4-hydroxyphenylpyruvate dioxygenase-like

Dcn	0.3131174	P	1704.82	5444.661	NM_007833	decorin
	0.31179053	P	1342.44	4305.589	XM_001478394	
Dmrt2	0.31099573	P	52.5615	169.0102	NM_145831	doublesex and mab-3 related transcription factor 2
Tnfaip6	0.3090259	P	20.3243	65.76904	NM_009398	tumor necrosis factor alpha induced protein 6
Gm6970	0.30727836	P	580.327	1888.603	XM_894271	predicted gene 6970
Pira2	0.3063984	P	14.5255	47.40733	NM_011089	paired-Ig-like receptor A2
Rab6b	0.30107412	P	80.907	268.7278	NM_173781	RAB6B, member RAS oncogene family
Gm2356	0.30069777	P	21.718	72.2254	XM_001473265	predicted gene 2356
	0.29940474	P	18.6964	62.44513	AK081001	
Ptgir	0.29816943	P	44.6263	149.6675	NM_008967	prostaglandin I receptor (IP)
Ska1	0.2981577	P	303.028	1016.335	NM_025581	spindle and kinetochore associated complex subunit 1
Grik5	0.29777563	P	92.062	309.1658	NM_008168	glutamate receptor, ionotropic, kainate 5 (gamma 2)
Oas1c	0.2976212	P	29.5363	99.24108	NM_033541	2'-5' oligoadenylate synthetase 1C
Aurkb	0.29660147	P	44.4488	149.8602	NM_011496	aurora kinase B
Usp18	0.29266334	P	20.5536	70.22953	NM_011909	ubiquitin specific peptidase 18
Gabrd	0.2926123	P	15.6859	53.60656	NM_008072	gamma-aminobutyric acid (GABA) A receptor, subunit delta
Zcchc13	0.29061842	P	16.9771	58.41708	NM_029158	zinc finger, CCHC domain containing 13
Kif9	0.28896594	P	12.4574	43.11037	NM_001163569	kinesin family member 9
4930571K23Rik	0.28863773	P	19.8041	68.6122	NM_001145759	RIKEN cDNA 4930571K23 gene
Mlf1	0.28791043	P	109.467	380.213	NM_010801	myeloid leukemia factor 1
Hist1h1b	0.28534627	P	1129.83	3959.512	NM_020034	histone cluster 1, H1b
Pira7	0.28317425	P	38.508	135.9869	NM_011094	paired-Ig-like receptor A7
Dchs1	0.27934897	P	88.765	317.7565	NM_001162943	dachsous 1 (Drosophila)
Prss16	0.27804947	P	15.2332	54.78575	NM_019429	protease, serine, 16 (thymus)
C330016O10Rik	0.27295542	P	56.2807	206.1902	NM_145974	RIKEN cDNA C330016O10 gene
Col24a1	0.27039227	P	23.8894	88.35104	NM_027770	collagen, type XXIV, alpha 1
Ap1s3	0.269494	P	65.5536	243.2469	NM_183027	adaptor-related protein complex AP-1, sigma 3
Nov	0.2687629	P	119.359	444.1059	NM_010930	nephroblastoma overexpressed gene
Gm6970	0.2646978	P	211.89	800.4959	XM_894271	predicted gene 6970

Gm9706	0.2637536	P	75.7706	287.278	XR_005074	predicted gene 9706
Fndc4	0.2625907	P	353.124	1344.769	NM_022424	fibronectin type III domain containing 4
2610002D18Rik	0.2609028	P	187.493	718.6331	NM_001081099	RIKEN cDNA 2610002D18 gene
Runx3	0.25908285	P	24.2878	93.74534	NM_019732	runt related transcription factor 3
Npnt	0.25784904	P	30.0745	116.6362	NM_033525	nephronectin
Pcsk4	0.25738412	P	33.7179	131.0021	NM_008793	proprotein convertase subtilisin/kexin type 4
	0.25435984	P	16.0501	63.10005	AK031951	
Agtr2	0.25297487	P	34.9821	138.2828	NM_007429	angiotensin II receptor, type 2
LOC100048763	0.25261217	P	26.5725	105.1907	XM_001481198	similar to ycf27 gene product
2010317E24Rik	0.24993527	P	180.243	721.1595	NM_001081085	RIKEN cDNA 2010317E24 gene
Lum	0.24317889	P	52.8735	217.4265	NM_008524	lumican
D430020J02Rik	0.23967148	P	11.8789	49.56305	NR_028421	RIKEN cDNA D430020J02 gene
Tnfaip8l1	0.2306665	P	72.3013	313.4453	NM_025566	tumor necrosis factor, alpha-induced protein 8-like 1
Ifi27l2a	0.23001792	P	301.699	1311.63	NM_029803	interferon, alpha-inducible protein 27 like 2A
Tnfaip6	0.22864796	P	65.8425	287.9645	NM_009398	tumor necrosis factor alpha induced protein 6
Tnfaip6	0.2235157	P	171.299	766.3851	NM_009398	tumor necrosis factor alpha induced protein 6
Lgr5	0.22083804	P	525.864	2381.219	NM_010195	leucine rich repeat containing G protein coupled receptor 5
Cxcl5	0.21848367	P	24.2823	111.1402	NM_009141	chemokine (C-X-C motif) ligand 5
Cyp4b1	0.2162263	P	16.1028	74.47189	NM_007823	cytochrome P450, family 4, subfamily b, polypeptide 1
Nipsnap1	0.1999804	P	152.13	760.723	NM_008698	4-nitrophenylphosphatase domain and non-neuronal SNAP25-like protein homolog 1
	0.19773169	P	27.7924	140.5561	AF031663	
Fndc1	0.19001609	P	222.503	1170.969	NM_001081416	fibronectin type III domain containing 1
Tmem121	0.17078203	P	202.528	1185.887	NM_153776	transmembrane protein 121
Tmem121	0.1676332	P	168.737	1006.586	NM_153776	transmembrane protein 121
Isg15	0.15776932	P	16.6773	105.7067	NM_015783	ISG15 ubiquitin-like modifier
Lrrc8e	0.135511	P	21.0422	155.2805	NM_028175	leucine rich repeat containing 8 family, member E
Gjb2	0.12474206	P	286.125	2293.729	NM_008125	gap junction protein, beta 2
Tnn	0.11664425	P	24.1796	207.2934	NM_177839	tenascin N
Asxl1	0.06179906	P	257.264	4162.917	NM_001039939	additional sex combs like 1 (Drosophila)

Supplementary Table 2. List of E2F target genes down-regulated more than 2-fold in *Asxl1*-null MEFs.

Gene	Accession number	Fold down	Description
Cell cycle			
Ccna2	NM_009828	2.2	cyclin A2
Ccnb1	NM_172301	2.4	cyclin B1
Ccnb2	NM_007630	2.0	cyclin B2
Ccne2	NM_001037134	2.6	cyclin E2
Cdk1	NM_007659	2.0	cdc2
Cdc25b	NM_023117	2.4	cdk1 activating phosphatase: G1 to S
Cdc25c	NM_009860	2.5	cdk1 activating phosphatase: G1 to S
Cdkn2c	NM_007671	2.3	cyclin-dependent kinase inhibitor 2C (p18Ink4c)
Rbl1	NM_001139516	3.1	retinoblastoma-like 1 (p107)
Mycn	NM_008709	2.2	neuroblastoma derived myc
Mybl2	NM_008652	2.4	myeloblastosis oncogene-like 2 (B-Myb)
E2F2	NM_177733	2.0	E2F transcription factor 2
Aurkb	NM_011496	3.4	aurora kinase B
DNA synthesis			
Cdc6	NM_011799	2.7	cell division cycle 6 homolog
Mcm3	NM_008563	2.0	minichromosome maintenance complex
Pola2	NM_008893	2.1	polymerase (DNA directed), alpha 2
Dhfr	NM_010049	2.3	dihydrofolate reductase
Tk1	NM_009387	2.0	thymidine kinase 1
Orc1l	NM_011015	2.1	origin recognition complex, subunit 1-like

Supplementary Table 3. Strand sequences used for shRNA expression.

Gene	Sense strand (5’ to 3’)	Antisense strand (5’ to 3’)
ASXL1 (human/ mouse)	GATCCGGATTCAACTTTCACGT ATTTCAAGAGAATACGTGAAAG TTGAATCCGGTTTTTTGGAAA	AGCTTTTCCAAAAAACCGGATTCAA CTTTCACGTATTCTCTTGAAATACG TGAAAGTTGAATCCGCG
ASXL1 (human-specific)	GATCCATGCCTTACCATCACAC TTCTCAAGAGAAAGTGTGATG GTAAGGCATGGTTTTTTGGAAA	GATCCATGCCTTACCATCACACTTC TCAAGAGAAAGTGTGATGGTAAGG CATGGTTTTTTGGAAA
ASXL1 for adenovirus	GCCTGAAAGCCATGATCATGT AAGCTTACATGATCATGGCTTT CAGGCTTTTTGC	GGCCGCAAAAAGCCTGAAAGCCAT GATCATGTAAGCTTACATGATCATG GCTTTCAG GC
Luciferase	GATCCGTTACTCGAGAAATATG ATTCTCAAGAGAAATCATATTT CTCGAGTAATTTTTTGGAAA	AGCTTTTCCAAAAAATTACTCGAGA AATATGATTTCTCTTGAGAATCATA TTTCTCGAGTAACG

Supplementary Table 4. List of antibodies for WB, IP, and ChIP.

Name	Company	Cat #	Mouse/Rabbit	Mono/Poly
Akt1	Santa Cruz	SC-8312	rabbit	poly
p-Akt(S473)	Santa Cruz	SC-7985-R	rabbit	poly
Rb	Santa Cruz	SC-50	rabbit	poly
p-Rb(S807/811)	Santa Cruz	SC-16670-R	rabbit	poly
E2F1	Santa Cruz	SC-193	rabbit	poly
p27Kip1	Santa Cruz	SC-528	rabbit	poly
p-p27Kip1(T157)	Abcam	ab85047	rabbit	poly
p53	Santa Cruz	SC-6243	rabbit	poly
p21Waf1	Santa Cruz	SC-6246	mouse	mono
p16Ink4a	Calbiochem	NA29	mouse	mono
β-actin	Santa Cruz	SC-47778	mouse	mono
Myc	MBL	M192-3	Mouse	mono
His	abm	G020	mouse	mono
HP1γ	Upstate	05-690	mouse	mono
Histone H3	Upstate	06-755	rabbit	poly
H3K9me3	Upstate	07-442	rabbit	poly
H3K27me3	Upstate	07-449	rabbit	poly
H3K4me2	Millipore	07-030	rabbit	poly
Histone H2A	Millipore	07-146	rabbit	poly
H2AUb	Cell signaling	8240	mouse	Mono
LSD1	Abcam	Ab17721	rabbit	poly
ASXL1 (for WB)	GeneTex	GTX127284	rabbit	poly
ASXL1 (for IP)	Our antibody		rabbit	poly
	Epitope covering amino acids 87-240, affinity-purified			

Supplementary Table 5. List of primer pairs for RT-qPCR.

Gene	Forward primer (5' to 3')	Reverse primer (5' to 3')
Mouse genes		
Asxl1	TCAGATGCTCCAATGACACC	AACAGCCCTTCTCCTCCTC
Ccna (Cyclin A)	GTCAGTAAACAGCCTGCCTT	CATGTGGTGATTCAAAACTGCC
Ccnd (Cyclin D)	GTTCGTGGCCTCTAAGATGA	CACTTGAGCTTGTTCACCAG
Ccne (Cyclin E)	GCCATCGACTCTTTAGAATTTCA	TGTCATCCCATTCCAAACCT
Cdc6	GGTCACCCTGGGGAAGTTA	CCTGAAAGGGACAAACACTCA
Orc1	GAGGAAGCAACCTTTCAACAGA	GGTATGGCAGTCCCTCCA
Gapdh	GACTCCACGACGTACTCA	GTGGATATTGTTGCCATC
Cdkn2a (p16Ink4a)	GCTCTGGCTTTCGTGAACAT	CGAATCTGCACCGTAGTTGA
Cdkn1a (p21Waf1)	CGGTGGAACTTTGACTTCGT	GGAAGTACTGGGCCTCTT
Cdkn1b (p27Kip1)	CGGTGCCTTTAATTGGGTCT	TGTTCTGTTGGCCCTTTTGT
Tp53	ATGGCCCCTGTCATCTTTTG	ATTGAGGGGAGGAGAGTACG
Cdkn1c (p57Kip2)	GGAGCAGGACGAGAATCAAG	CGAAGAAGTCGTTCGCATTG
Mmp1	GAACTGGGTCACTCCCTTGG	GCTGGATGGGATTTGGGGAA
Pai1	GGCCAATGGAAGACCCCTTT	GCTGGTAGGGCAGTTCCAC
Cdc25c	ACCTAGGACCCCAAGGTTTC	GACTGCCCAGATGCTTCATT
Human gene		
CDKN2A	AGCAGCATGGAGCCTTCGGC	ATCATGACCTGGATCGGCCT
GAPDH	GACTCCACGACGTACTCA	GTGGATATTGTTGCCATC

Supplementary Table 6. Lists of primer pairs for ChIP analysis.

Gene	Forward primer (5' to 3')	Reverse primer (5' to 3')
mCcna2 (Cyclin A2)	GTAAGATTCCCGTCGGGCC	AGGCGGAGGAGCGTAGA
mCdkn2a (p16Ink4a)	CTTCGGAGGGCCTTTCCTAC	GTCGCAGGTTCTTGGTCACT

Original uncropped immunoblots

- Main figures (2, 4)

Fig. 2b, lane 1

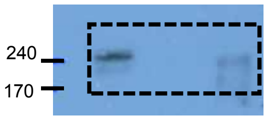

Fig. 2b, lane 2

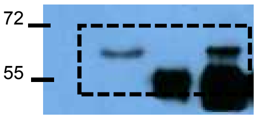

Fig. 2c, lane 1 and 3

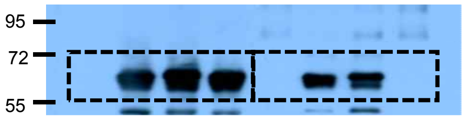

Fig. 2c, lane 2

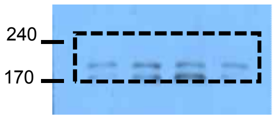

Fig. 2d

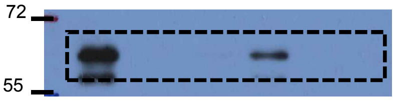

Fig. 2e, lane 1

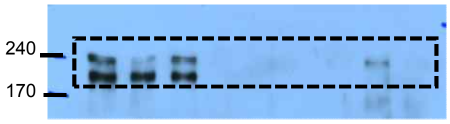

Fig. 2e, lane 2, right

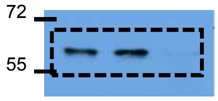

Fig. 2f, lane 1

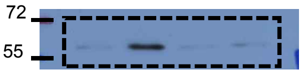

Fig. 2e, left

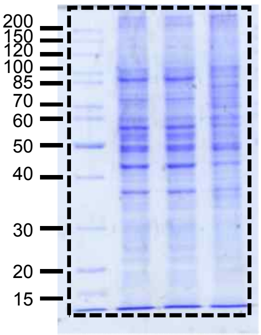

Fig. 2e, lane 3, right

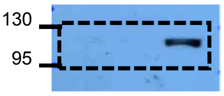

Fig. 2f, lane 2

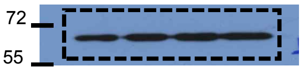

Fig. 2e, lane 4, right

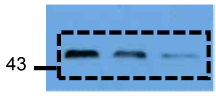

Fig. 2f, lane 3

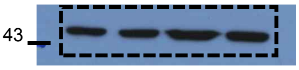

Fig. 4a, lane 1

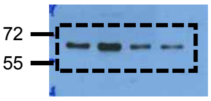

Fig. 4a, lane 4

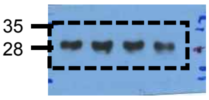

Fig. 4b, lane 1, left

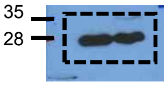

Fig. 4b, lane 1, right

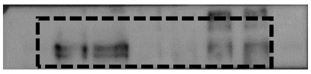

Fig. 4a, lane 2

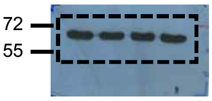

Fig. 4a, lane 5

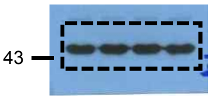

Fig. 4b, lane 2, left

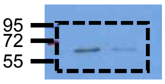

Fig. 4b, lane 2, right

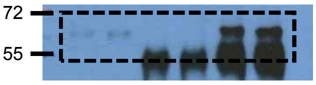

Fig. 4a, lane 3

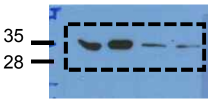

Fig. 4d, lane 1

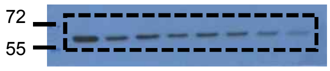

Fig. 4d, lane 4

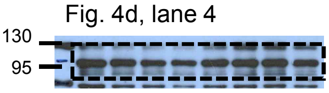

Fig. 4d, lane 2

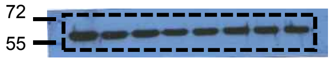

Fig. 4d, lane 5

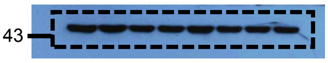

Fig. 4d, lane 3

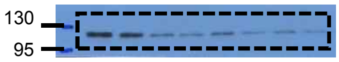

- Main figures (5, 7)

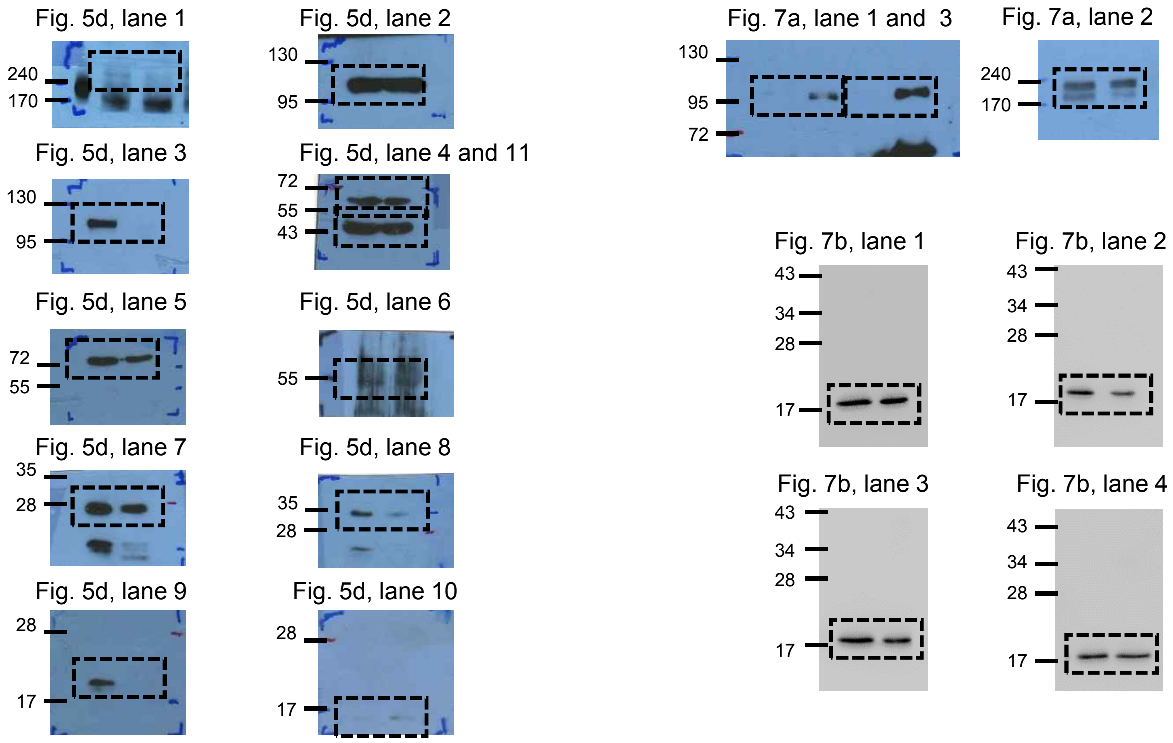

- Supplementary figures (2, 6, 7)

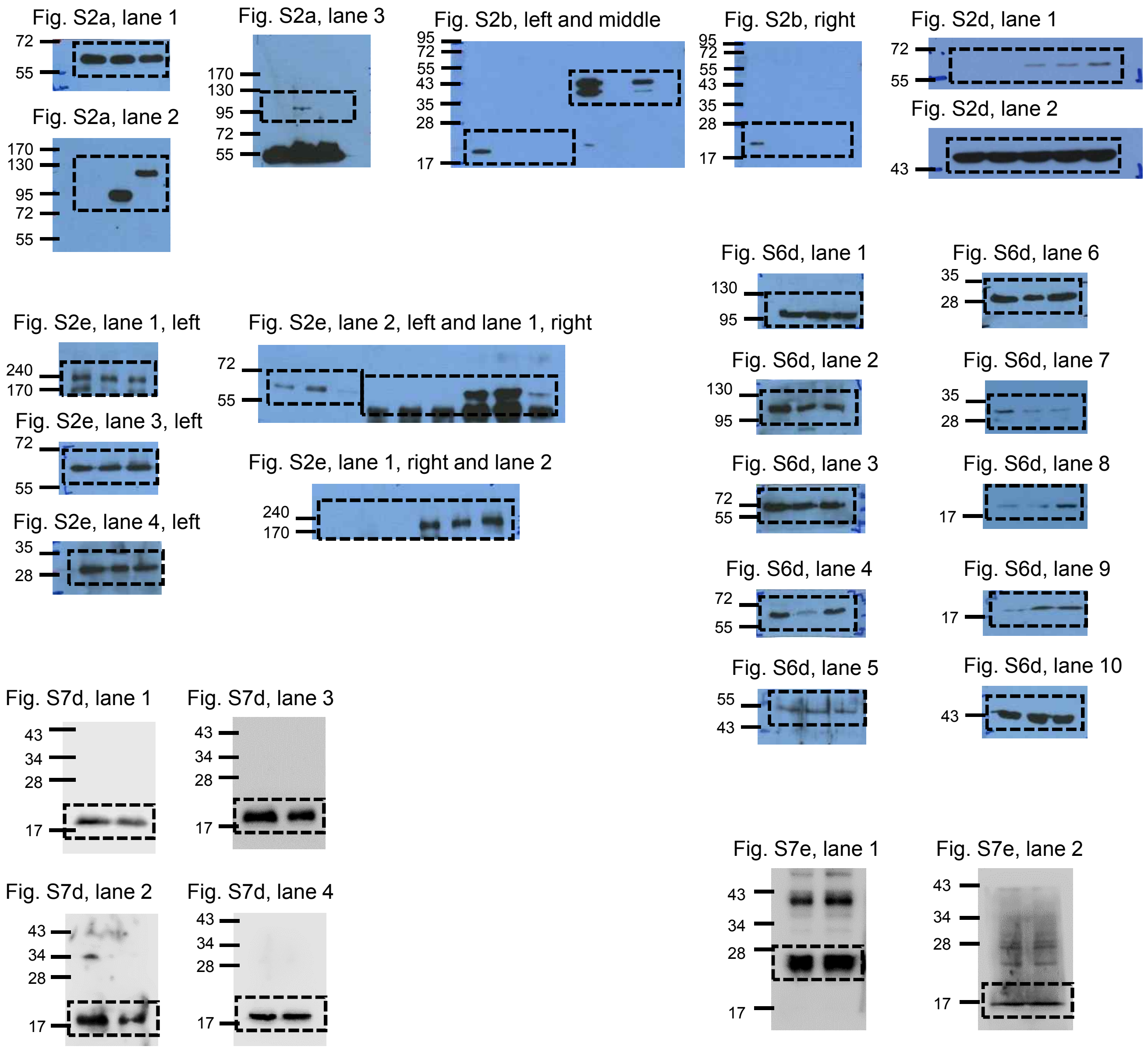

Supplement: Supplementary file 1 — Supplementary data [file 41598_2017_5564_MOESM1_ESM.pdf]
